# The economic linkages of Covid-19 across sectors and regions in Europe

**DOI:** 10.1007/s13209-025-00305-0

**Published:** 2025-03-15

**Authors:** Fidel Pérez-Sebastián, Rafael Serrano-Quintero

**Affiliations:** 1 Department of Economics (FAE), University of Alicante, Alicante, Spain; 2 Department of Economics, BEAT and CREB, University of Barcelona, Barcelona, Spain

**Keywords:** Spatial economics, Covid-19, First wave, Policy response, Quantitative models, E10, I10, R10

## Abstract

This paper builds a spatial model of trade with supply-chain links to examine the effect of economic links and anti-COVID policies on the spread of the COVID-19 pandemic during the first wave across NUTS2 regions of the European Union (EU) and the UK. We find that the effort to reduce infection rates was more successful in the UK than in the EU, and that the deaths due to the trade vector were 10% on average across Europe. Our results imply that without the policy response in Europe, the number of deaths during the first wave would have been about 4,520,000 higher in the EU and around 1,240,000 greater in the UK, with significant variations across regions. Oberbayern in Germany and South Yorkshire in the UK appear as the most effective in reducing the death burden of COVID-19 at different points during the first wave. Moreover, 42% and 37% of the total deaths in the UK and the EU, respectively, could have been prevented if the policy implemented in these two regions had prevailed throughout Europe.

## Introduction

The COVID-19 pandemic ended 4.55 million lives (as of October 1, 2021), forced quarantines all over the world, stopped global value chains for a significant amount of time, and created one of the largest global recessions in recent years. However, as with the spread of other infectious diseases, its impact in terms of lives and economic activity varied greatly across regions and industries (see, e.g., Villani et al. (2020) and de Vet et al. (2021)). In this paper, we build on the idea that diffusion of infectious diseases depends on human interactions (e.g., see Fogli and Veldkamp (2021)), and in particular, on how dense is the economic network of a given area. We consider endogenously determined economic interactions and analyze the effect of the policies adopted to fight the first wave of the pandemic across different regions in the United Kingdom (UK) and the European Union (EU). More specifically, the paper asks the following questions. What is the contribution of economic linkages to the expansion of the disease? How many lives have the polices implemented saved?

The model we develop embeds a spatial economic model in the spirit of Allen and Arkolakis (2014), Caliendo and Parro (2015), and Caliendo et al. (2017) into the canonical susceptible, infected, and recovered (SIR) model by Kermack et al. (1927). The purpose of the proposed framework is to analyze the two-way causation between the spatial dynamics of an epidemic and the spatial distribution of economic activity. More specifically, the setup incorporates Ricardian trade *á la* Eaton and Kortum (2002), and extends the SIR model in two ways. First, similar to Fernández-Villaverde and Jones (2022), we consider five population groups composed of susceptible, vaccinated, infected, resolving, and recovered individuals, and also account for deaths. Second, we allow for spatial connections that are endogenously determined by the structure of our economic geography model. The assumption is that when regions trade, people enter in contact with one another so they put themselves at risk of getting infected or that the virus is itself transported through the imported goods. As a result of the economic geography model, denser regions will experience more rapid increase in infections for two reasons. First, within the region, there are more interactions across individuals and thus, a higher probability of transmission. Second, the larger a region is, the more it will trade with other regions, and thus, the higher the probability of transmitting the disease across regions.

In our framework, the economy is composed of a set of locations that produce goods in different sectors. Each sector produces three goods: a final product, an intermediate good, and a composite intermediate or material. The first two can be traded but trade is costly. The third one is only sold domestically within the region. In addition, following Caliendo and Parro (2015), whereas the domestic movement of materials is inter-industry, cross-regional trade of intermediate goods is purely intra-industry.^1^ This feature captures that the latter type of trade represents the largest component of the trade flows of intermediates. For example, World Bank (2009) finds that, from 1962 to 2006, worldwide intra-industry trade in intermediate goods increased approximately from $30\%$ to $60\%$ of total trade. This share equals $42\%$ in our European Union 28-country group (EU28) dataset, which include the current European Union plus the UK for the year 2013. What is most important is that these inter- and intra-industry links across sectors mean that policies and changes that affect a given industry can potentially affect all other sectors and regions. Our main contribution is to assess how the heterogeneity in production structures and regional connections affect the spread of the disease and its economic impact.

The model proceeds in two phases. For the population composition in a given day, the first phase obtains the distribution of economic activity and bilateral trade shares. In the second phase, we take as given the bilateral trade shares and the spatial distribution of economic activity along with the disease ecology to determine how the population composition changes from one day to the next. This creates a loop in which disease dynamics and economic activity affect each other. In particular, disease prevalence can reduce the labor force in a region through either mortality, morbidity or policy actions. These shocks affect the level of economic activity and reduce international trade. The modification of the trade patterns, in turn, has an impact on the spread of the disease by decreasing the amount of infection *“exported”* to other regions. These general equilibrium forces resemble a behavioral response in which agents protect themselves from the infection.

The explicit modeling of the geography is important to understand the disease dynamics.^2^ In general, those regions that are more isolated will receive and transmit less the infection. As an example, take the evolution of the pandemic in Spain versus Italy and the UK. The spread of the infection in Spain was faster in Madrid (a region in the center of the country) and then expanded throughout the nation. In Italy, the infection started in the north and then moved slowly toward the south. In the UK, in turn, the disease was more concentrated in the south but, at the same time, more widespread than in other parts of Europe. Our model addresses these singularities through the explicit modeling of the geography of trade in Europe.

We calibrate the model to match the distribution of workers and wages across 230 regions from 28 countries in Europe for 10 sectors of production comprising the whole economy and use our framework to assess through a set of counterfactuals, how policies adopted during the coronavirus pandemic, which include social distancing and regional lockdowns, have affected the impact of the disease. We focus on the first wave that goes from February 25 to July 15, 2020. For each of these regions, we use data on fatalities by COVID-19 to back out an estimate of the infection rate using the structure of the model. Essentially, this estimated infection rate is a residual that makes sure the model can track the evolution of the disease for each region, which is the same approach as the one followed by Fernández-Villaverde and Jones (2022) at a country level. We interpret changes in this infection rate as policies against COVID-19 and validate this interpretation in Sect. 6.1 by relating our recovered infection rate on several indices that track government responses against COVID-19. We find a strong relationship with containment and health-related policies, and for the government response index. These results suggest that our recovered infection rate in fact reflects anti-COVID policy responses.

The data indicates that despite a higher incidence of the disease in the UK compared to the European Union, the fight to reduce the infection rates was more successful in the UK than in the European Union. Our assessment reveals that the trade vector contributed to $10\%$ of the fatalities caused by COVID-19. We also conduct policy counterfactuals, where policy interventions are captured by a residual-like parameter. We simulate two types of scenarios. In the first one, policy responses are shut down over time in different geographical areas. In the second type, we allow all regions to enjoy the disease-transmission probabilities of the most successful areas regarding COVID-19 containment.

The results imply that without the policy reaction in Europe, the number of deaths during the first wave of the pandemic would have been about 4,520,000 larger in the European Union and about 1,240,000 higher in the UK, with significant variation across regions. Comparing the effects of the policies implemented in the EU27 and in the UK, we estimate that in the absence of European Union’s anti-COVID measures, the number of deaths in the UK would have been an 83% higher, equivalent to an additional 36 deaths per 100,000 inhabitants. Conversely, the UK’s anti-COVID measures saved 51,706 lives in the European Union and about 1,200,188 lives in the UK.

Our calculations indicate that the policies implemented by the region of Oberbayern in Germany were the most effective in reducing the death burden of COVID-19 across Europe during the first two months (or 61 days) after the onset of the disease. Subsequently, South Yorkshire in the UK was the area that managed to maintain control over the infection more effectively. Utilizing the daily decrease in transmission probabilities implied by the combination of these two areas, we find that 12,752 and 38,883 lives, amounting to 42% and 37% of the total deaths, could have been saved in the UK and the European Union, respectively, if the disease ecology and policy implemented in the two aforementioned regions had prevailed throughout Europe.

The paper proceeds as follows. Section 2 describes the related literature. Section 3 provides evidence in support of the mechanisms highlighted by our model. Section 4 introduces the model. The calibration of its exogenous variables and parameters is discussed in Sect. 5. Section 6 presents the results. Section 7 concludes.

## Related literature

Our paper contributes to a large and growing literature on the link between economic activity and infectious diseases.^3^ We contribute by constructing and calibrating a model that features a set of European regions at different stages of development and assesses the importance of trade on the spread of the COVID-19 pandemic. In this model, trade serves as a vector of disease transmission through the transportation of goods and people.

It is well documented that goods transportation across regions can help spread infectious diseases.^4^ In the context of the HIV infection in Africa, Oster (2012) shows that the movement of truckers engaged in exports leads to a significant increase in new infections. Specifically, she estimates that doubling exports increases HIV infections by 10–70% through truckers. In a similar vein, Bajunirwe et al. (2020) analyze the spread of COVID-19 in Uganda through trucks drivers. They find that the very first cases arrived through international arrivals from Asia and Europe. However, by 29 April, out of the total amount of travellers with a tested confirmed case, $71.8\%$ were long-distance trucks drivers, while only $11.3\%$ were international arrivals. Furthermore, the majority of community cases were associated with contact with truck drivers. Similarly, Martini et al. (2022) find that the infection was significantly common among truck drivers in Uganda, Kenya, Rwanda, and South Sudan.

In a Latin American context, Calatayud et al. (2022) focus on the spread of COVID-19 in Colombia through the trucking network. They demonstrate that the number of confirmed COVID-19 cases in a municipality is positively linked to its level of trucking network centrality. Bernardes-Souza et al. (2021) perform a household survey and a case–control study in two towns in Brazil between May and June 2020, and find that logistics workers were the main source of COVID-19 contagion among households.

In Asia and Europe, similar patterns emerge. For example, Lan et al. (2020) study the progression of COVID-19 in Hong Kong, Japan, Singapore, Taiwan, Thailand, and Vietnam during the first 40 days after the initial locally transmitted case. They discard all imported cases to identify the occupation groups with most work-related cases. They find healthcare workers $(22\%)$ and driver and transport workers $(18\%)$ to be the two main occupations affected by work-related cases. Adda (2016) provides evidence, based on microdata, that the expansion of transportation networks and inter-regional trade had a significant impact on virus spreading in France. Focusing on France, Italy, and Spain, Bontempi et al. (2021) find that there is a strong statistical relationship between international trade intensity and severity of the COVID-19 pandemic across their regions.

Human mobility, in general, and tourism, in particular, emerge as significant vectors of COVID-19 transmission. Iacus et al. (2020) use variation in mobility restrictions across countries of the European Union and find that Human mobility alone explains $92\%$ of France and Italy initial spread of COVID-19. Farzanegan et al. (2021) analyze the relationship between the COVID-19 spread and international tourism across countries. They find that, up until April 30, 2020, those countries receiving larger inflows of international tourists experienced a higher level of confirmed COVID-19 cases and deaths even when normalizing these COVID-19 outcomes by the country’s population. Domestic tourism acted as well as a vector of the COVID-19 virus spread. For instance, Robin Nunkoo and Gholipour (2022) find that countries with a higher level of domestic travel related to tourism experienced higher levels of cases and deaths during the first six months of the pandemic.

Based on the empirical evidence above and the one we provide in Sect. 3, we argue that trade and tourism, when people mobility was not restricted, are important vectors of COVID-19 transmission. Therefore, our paper offers an alternative, complementary channel to the business travel one proposed by Antràs et al. (2023). Antràs et al. (2023) construct a two-country framework of human interactions through business travel, combining a gravity equation structure and an epidemiological model of disease evolution. In their model, the disease spreads as agents travel between countries. We depart from them by building a multi-country and multi-sector setup with an input–output structure rich enough to capture the transmission of the disease through bilateral trade across all network nodes. Furthermore, a crucial ingredient in our model is that it incorporates a time-variant local infection rate that tracks local policies against COVID-19. Then, our simulations are able to compare the effectiveness of regional policies at the NUTS2 level against COVID in the UK and the EU.

We are not the first in introducing spatial connections in epidemiological models. Lloyd and May (1996) and Keeling (1999) are early examples of spatial models of epidemics. Paeng and Lee (2017) extend the canonical SIR model by including spatial infections assuming that the infection can be spread in a given radius. In the epidemiological literature, the connection between trade and the spread of infectious diseases is also known, and Mayer (2000) notes that vectors of transmission of dengue fever or cholera were introduced in the USA through imported tires and through dumping bilge water into the ocean. We depart from this literature by endogenizing the spatial connections within a quantitative economic geography model, instead of assuming a given radius of infection or stochastic encounters.

Spatial frameworks in which the spread of the disease can occur through the movement of goods and people are also considered by Cuñat and Zymek (2022) and Giannone et al. (2022). Cuñat and Zymek (2022) combine a simple epidemiological framework with a dynamic model of individual location choice to study the impact of quarantines and other mobility restrictions on the spread of COVID-19 in the UK. In turn, Giannone et al. (2022) studies optimal containment policies in an economy with connected regions focusing on the USA. Unlike them, we consider a richer model of Ricardian trade and take advantage of a data set on sectoral bilateral trade flows between European Regions, the Rhomolo-MRIO Tables for 2013 published by the European commission (Thiessen 2020).

Other recent papers have studied the role of specific policies. For example, focusing on optimal lockdown policies, Acemoglu et al. (2021) emphasize differences across population groups, Alvarez et al. (2021) discuss the intensity and duration of the policy, and Glover et al. (2023) analyze the distributional consequences of policies that shut down sectors. More closely to our context, Fajgelbaum et al. (2021) find that regional-specific lockdowns result in better outcomes than uniform lockdowns. We depart from them by analyzing the policy effects at a higher regional level. We also depart from them by considering a compounded policy measure that captures broader policies against COVID-19.^5^

Our article also talks to another branch of recent papers focused on consumer behavior and output responses when faced with an infectious disease. Reductions in production during COVID have been explained with lockdowns (Bonadio et al. 2021; Sforza and Steininger 2020), supply shocks (Eppinger et al. 2021; Guerrieri et al. 2022; Kejžar et al. 2022), short-term demand reductions (Krueger et al. 2022; Eichenbaum et al. 2021; Liu et al. 2022), or risk aversion of forward-looking producers (Baker et al. 2020), to mention a few. Crucially, we depart from them by looking at the effects on the spread of COVID-19 within a multiple-region setting and a rich input–output structure.

Finally, Çakmaklı et al. (2021) study how demand and supply shocks affect global vaccinations and how vaccinations of other countries can potentially benefit home countries. They do not include, however, endogenous links for the spread of the infection. We also extend the methodology by Fernández-Villaverde and Jones (2022) to recover infection rates based on future deaths and use it to calibrate our model with endogenous links in the disease.

## Empirical motivation

Trade, as a vector of COVID-19 transmission through the transportation of goods and tourism, is a key mechanism in our paper. While the movement of people was restricted in many countries during the first wave of the pandemic, freight transportation was never entirely halted. In contrast, tourism was among the most affected sectors, facing severe disruptions due to travel restrictions, lockdowns, and public health protocols. However, these restrictions were not implemented uniformly across time or nations, a factor our analysis will consider. For example, United Kingdom did not impose restrictions to international traveling until June 8, and Latvia never imposed restrictions to the internal movement of people. Consequently, both freight transportation and tourism may have contributed to the spread of COVID-19 in different ways, depending on the specific circumstances in each location and period.

To provide additional empirical support for this mechanism, we assess whether there is a relationship between deaths by COVID-19 and past tourist visits. In particular, we use variation across the regions in our analysis (see Table 1) to explore this relationship. We use data on arrivals at tourist accommodation establishments by NUTS2 regions from Eurostat and the compiled data on deaths by COVID-19 from different sources (see Table 5).

**Table 1:** NUTS2 regions

Code	Region	Code	Region
AT11	Burgenland (AT)	BE1	Région Bruxelles-Capitale / Brussels H G
AT12	Niederösterreich	BE2	Vlaams Gewest
AT13	Wien	BE3	Région wallonne
AT21	Kärnten	BG	Bulgaria
AT22	Steiermark	CYP	Kypros
AT31	Oberösterreich	CZ01	Praha
AT32	Salzburg	CZ02	Strední Cechy
AT33	Tirol	CZ03	Jihozápad
AT34	Vorarlberg	CZ04	Severozápad
DE11	Stuttgart	CZ05	Severovýchod
DE12	Karlsruhe	CZ06	Jihovýchod
DE13	Freiburg	CZ07	Strední Morava
DE14	Tübingen	CZ08	Moravskoslezsko
DE21	Oberbayern	DE30	Berlin
DE22	Niederbayern	DE40	Brandenburg
DE23	Oberpfalz	DE50	Bremen
DE24	Oberfranken	DE60	Hamburg
DE25	Mittelfranken	DE71	Darmstadt
DE26	Unterfranken	DE72	Gießen
DE27	Schwaben	DE73	Kassel
DE80	Mecklenburg-Vorpommern	DEA1	Düsseldorf
DE91	Braunschweig	DEA2	Köln
DE92	Hannover	DEA3	Münster
DE93	Lüneburg	DEA4	Detmold
DE94	Weser-Ems	DEA5	Arnsberg
DED2	Dresden	DEE0	Sachsen-Anhalt
DED4	Chemnitz	DEF0	Schleswig-Holstein
DED5	Leipzig	DEG0	Thüringen
DK01	Hovedstaden	DK02	Sjælland
DK03	Syddanmark	DK04	Midtjylland
DK05	Nordjylland	EE00	Eesti
EL11	Anatoliki Makedonia, Thraki	EL12	Kentriki Makedonia
EL13	Dytiki Makedonia	EL14	Thessalia
EL21	Ipeiros	EL22	Ionia Nisia
EL23	Dytiki Ellada	EL24	Sterea Ellada
EL25	Peloponnisos	EL30	Attiki
EL41	Voreio Aigaio	EL42	Notio Aigaio
EL43	Kriti	ES11	Galicia
ES12	Principado de Asturias	ES13	Cantabria
ES21	País Vasco	ES22	Comunidad Foral de Navarra
ES23	La Rioja	ES24	Aragón
ES30	Comunidad de Madrid	ES41	Castilla y León
ES42	Castilla-la Mancha	ES43	Extremadura
ES51	Cataluña	ES52	Comunidad Valenciana
ES53	Illes Balears	ES61	Andalucía
ES62	Región de Murcia	ES63	Ciudad Autónoma de Ceuta (ES)
ES64	Ciudad Autónoma de Melilla (ES)	ES70	Canarias (ES)
FI19	Länsi-Suomi	FI1B	Helsinki-Uusimaa
FI1C	Etelä-Suomi	FI1D	Pohjois- ja Itä-Suomi
FI20	Åland	FR10	Île de France
FR21	Champagne-Ardenne	FR22	Picardie
FR23	Haute-Normandie	FR24	Centre (FR)
FR25	Basse-Normandie	FR26	Bourgogne
FR30	Nord-Pas-de-Calais	FR41	Lorraine
FR42	Alsace	FR43	Franche-Comté
FR51	Pays de la Loire	FR52	Bretagne
FR53	Poitou-Charentes	FR61	Aquitaine
FR62	Midi-Pyrénées	FR63	Limousin
FR71	Rhône-Alpes	FR72	Auvergne
FR81	Languedoc-Roussillon	FR82	Provence-Alpes-Côte d’Azur
FR83	Corse	HRV	Croatia
HU	Hungary	IE	Ireland
ITC1	Piemonte	ITC2	Valle d’Aosta/Vallée d’Aoste
ITC3	Liguria	ITC4	Lombardia
ITF1	Abruzzo	ITF2	Molise
ITF3	Campania	ITF4	Puglia
ITF5	Basilicata	ITF6	Calabria
ITG1	Sicilia	ITG2	Sardegna
ITH1	Provincia Autonoma di Bolzano/Bozen	ITH2	Provincia Autonoma di Trento
ITH3	Veneto	ITH4	Friuli-Venezia Giulia
ITH5	Emilia-Romagna	ITI1	Toscana
ITI2	Umbria	ITI3	Marche
ITI4	Lazio	LTU	Lietuva
LUX	Luxembourg	LVA	Latvija
MLT	Malta	NL	Netherlands
PL11	Lódzkie	PL12	Mazowieckie
PL21	Malopolskie	PL22	Slaskie
PL31	Lubelskie	PL32	Podkarpackie
PL33	Swietokrzyskie	PL34	Podlaskie
PL41	Wielkopolskie	PL42	Zachodniopomorskie
PL43	Lubuskie	PL51	Dolnoslaskie
PL52	Opolskie	PL61	Kujawsko-Pomorskie
PL62	Warminsko-Mazurskie	PL63	Pomorskie
PT11	Norte	PT15	Algarve
PT16	Centro (PT)	PT17	Área Metropolitana de Lisboa
PT18	Alentejo	PT20	Região Autónoma dos Açores (PT)
PT30	Região Autónoma da Madeira (PT)	RO	Romania
ROW	Rest of the world	SE11	Stockholm
SE12	Östra Mellansverige	SE21	Småland med öarna
SE22	Sydsverige	SE23	Västsverige
SE31	Norra Mellansverige	SE32	Mellersta Norrland
SE33	Övre Norrland	SI01	Vzhodna Slovenija
SI02	Zahodna Slovenija	SK01	Bratislavský kraj
SK02	Západné Slovensko	SK03	Stredné Slovensko
SK04	Východné Slovensko	UKC1	Tees Valley and Durham
UKC2	Northumberland and Tyne and Wear	UKD1	Cumbria
UKD3	Greater Manchester	UKD4	Lancashire
UKD6	Cheshire	UKD7	Merseyside
UKE1	East Yorkshire and Northern Lincolnshire	UKE2	North Yorkshire
UKE3	South Yorkshire	UKE4	West Yorkshire
UKF1	Derbyshire and Nottinghamshire	UKF2	Leicestershire, Rutland and Northamptonshire
UKF3	Lincolnshire	UKG1	Herefordshire, Worcestershire and Warwickshire
UKG2	Shropshire and Staffordshire	UKG3	West Midlands
UKH1	East Anglia	UKH2	Bedfordshire and Hertfordshire
UKH3	Essex	UKI1	Inner London-West
UKI2	Inner London-East	UKJ1	Berkshire, Buckinghamshire and Oxfordshire
UKJ2	Surrey, East and West Sussex	UKJ3	Hampshire and Isle of Wight
UKJ4	Kent	UKK1	Gloucestershire, Wiltshire and Bristol/Bath area
UKK2	Dorset and Somerset	UKK3	Cornwall and Isles of Scilly
UKK4	Devon	UKL1	West Wales and The Valleys
UKL2	East Wales	UKM2	Eastern Scotland
UKM3	South Western Scotland	UKM5	North Eastern Scotland
UKM6	Highlands and Islands	UKN0	Northern Ireland (UK)

Specifically, we regress the log of cumulative deaths by COVID-19 up to July 15, 2020, on the log of tourist arrivals in 2020 controlling for the log of population density, that is, we estimate

1 $$\begin{aligned} \log (\text {Deaths}_{g,c}) = \alpha + \beta _1 \log (\text {Density}_{g,c}) + \beta _2 \log (\text {Tourists}_{g,c}) + \mu _c + \varepsilon _{g,c}, \end{aligned}$$

where $\text {Deaths}_{g,c}$ is the cumulative number of deaths by COVID-19 in region *g* of country *c*, $\text {Density}_{g,c}$ is the population density in region *g* of country *c*, $\text {Tourists}_{g,c}$ is the number of tourists in region *g* of country *c* (total, domestic, or foreign), $\mu _c$ is a country fixed effect, and $\varepsilon _{g,c}$ is the error term.

We estimate Eq. (1) by OLS and find that tourist arrivals are positively associated with more deaths by COVID-19 (i.e., $\beta _2 > 0$), and this relationship is statistically significant. Panel A in Table 2 presents these results. Although this is purely correlational suggestion, it could be the case that there could be a negative reverse causality bias (i.e., more deaths in a region reduce the number of visits). To mitigate that we estimate the same regression (1) instrumenting by the log of number of tourists in 2018 and 2019. The estimated coefficients for the three tourism variables are positive and economically and statistically significant, thus reinforcing our proposed mechanism. Panel B of Table 2 presents the IV estimates. These are slightly larger than the OLS estimates suggesting that the negative reverse causality bias was there.

**Table 2:** Arrivals of tourists and COVID-19 deaths by region

	(1)	(2)	(3)
*Panel A: OLS*			
Log population density	0.114	0.234**	$-$0.118
	(0.139)	(0.111)	(0.206)
Log total arrivals (2020)	1.240***		
	(0.196)		
Log domestic arrivals (2020)		1.411***	
		(0.293)	
Log foreign arrivals (2020)			0.767***
			(0.075)
Num.Obs	147	146	145
$R^{2}$	0.543	0.575	0.491
*Panel B: IV*			
Log population density	0.120	0.252**	$-$0.121
	(0.134)	(0.101)	(0.202)
Log total arrivals (2020)	1.282***		
	(0.190)		
Log domestic arrivals (2020)		1.505***	
		(0.302)	
Log foreign arrivals (2020)			0.805***
			(0.090)
Num.Obs	145	144	143
$R^{2}$	0.543	0.574	0.491

*$p< 0.1$, **$p < 0.05$, ***$p< 0.01$. The dependent variable is the log of cumulative deaths by COVID-19. Controls are the log of population density, total, domestic, and foreign arrivals in 2020. The IV regressions instrument the log of arrivals with the log of arrivals in 2018 and 2019

To further explore this mechanism, we also regress the log of cumulative deaths on a dummy variable that takes value equal to 1 if the region is in the upper quartile (75th percentile) of the arrivals distribution in the years 2020, 2019, and 2018, and we also distinguish between domestic, foreign, and total arrivals. The results are provided in Table 3. As before, the coefficients are positive and statistically significant reinforcing the evidence of this mechanism.

**Table 3:** Arrivals of tourists and COVID-19 deaths by region

	Total	Domestic	Foreign
Log population density	$-$0.092	$-$0.080	$-$0.212
	(0.327)	(0.315)	(0.340)
Upper quartile in total arrivals	2.036***		
	(0.440)		
Upper quartile in domestic arrivals		1.378**	
		(0.592)	
Upper quartile in foreign arrivals			2.133***
			(0.712)
Num.Obs	147	146	146
$R^{2}$	0.421	0.420	0.446

*$p< 0.1$, **$<p 0.05$, ***$p< 0.01$. The dependent variable is the log of cumulative deaths by COVID-19. Controls are the log of population density, and dummy variables that is equal to 1 if the region is in the upper quartile of the total, domestic, or foreign arrivals in 2020, 2019, and 2018

## The model

We assume the economy is composed of a set of *G* geographical locations or regions that belong to different countries and *J* sectors or industries. Regions are denoted by *g*, *i* and *h* and sectors by *j* and *k*. In each industry, there is production of a composite intermediate or material, an array of different varieties of intermediate goods, and a set of different types of final consumption goods. Households provide labor to the production process. Labor is mobile across sectors and immobile across locations. All markets are perfectly competitive.

The model offers a rich supply-chain structure. Local materials from different sectors are employed along with the labor input to produce intermediate goods. In the next stage, intermediate goods produced by the same industry possibly in different locations are combined to generate final consumption products and a composite intermediate or material. These connections among the different stages of the production chain can provide amplification effects of trade disruptions.

We assume that the intermediate goods and final products can be tradable or not, whereas materials are not tradable. We consider that final consumption products can cross-regional borders, because some of them, like tourism, can be important for the propagation of the virus and are tradable. Trade in intermediate goods is intra-industry, which represents the largest component of the world trade flows of intermediates.

Let us now move to describing the model demographics. For simplicity, we omit time subscripts. The size of the population in region *g* equals $N_{g}$. This population is composed of five groups: susceptible vaccinated and susceptible non-vaccinated people—denoted by $V_{g}$ and $S_{g}$, respectively—who are not infected but can develop the disease; infected individuals, $I_{g}$; resolving cases $R_{g}$ who can pass away with probability $\delta$ or recover with probability $(1-\delta )$^6^; and recovered$\ C_{g}$, who can potentially get reinfected. Hence, it must be satisfied that

2 $$\begin{aligned} N_{g}=S_{g}+V_{g}+I_{g}+R_{g}+C_{g}. \end{aligned}$$

We will consider the possibility that recovered and vaccinated individuals may rejoin the susceptible non-vaccinated population once the partial immunity acquired by being exposed to the virus or the vaccine is lost.

Only a fraction $l_{gH}$ from each group *H* can supply labor services. This fraction $l_{gH}$ will be taken as exogenous, given by morbidity and policy considerations. Then, the available labor force $L_{g}$ equals:

3 $$\begin{aligned} L_{g}=l_{gS}S_{g}+l_{gV}V_{g}+l_{gI}I_{g}+l_{gR}R_{g}+l_{gC}C_{g}. \end{aligned}$$

With these ingredients, the model is numerically solved through a loop that consists of two phases, with each period corresponding to one day. In the first phase, for the population composition in a given day, we obtain the spatial distribution of economic activity. The second phase takes as given the spatial distribution delivered by the first phase, along with the disease ecology to determine how the population composition changes from one day to the next. We consider that the infection can spread within and across locations because of people contact. Finally, the new population composition feeds again the first phase, and this loop continues until predictions for the desired number of days are generated.

### Phase 1: economic allocations across space

The first phase of the model determines the underlying economic geography through which the virus and the economic consequences of policies will potentially spread.

#### Households

Welfare-maximizing consumers in each location have identical preferences given by^7^:

4 $$\begin{aligned} W_{g}=\prod _{j=1}^{J}\left( c_{g}^{j}\right) ^{\alpha _{g}^{j}}; \end{aligned}$$

where

5 $$\begin{aligned} c_{g}^{j}=\left[ \int _0^1 c_{g}^{j}(\Omega ^{j})^{1-1/\varsigma ^{j}}d\Omega ^{j}\right] ^{\varsigma ^{j}/(\varsigma ^{j}-1)}; \end{aligned}$$

the parameter $\alpha _{g}^{j}$ represents the share of sector-*j* products in total consumption expenditure in location *g*, that is, $\sum _{j=1}^{J} \alpha _{g}^{j}=1$; the variable $c_{g}^{j}(\Omega ^{j})$ denotes the units consumed in location *g* of variety $\Omega ^{j}$ from sector-*j* ($\Omega ^{j}$ is one among a mass of size *one* of different varieties); and the parameter $\varsigma ^{j}$ gives the elasticity of substitution between different varieties of sector-*j* consumption products.

In each location, the population size $N_{g}$ is divided between workers $L_{g}$ and non-workers $N_{g}-L_{g}$. Each of the two consumer types has, in principle, a distinct budget constraint, because income may differ depending on whether they work or not. However, we assume that workers pay lump-sum unemployment insurance ($t_{g}$) at the location where they provide labor services, and these taxes are fully redistributed as unemployment benefits ($s_{g}$) to the non-working individuals at the local level, that is, $t_{g}L_{g}=s_{g}(N_{g}-L_{g})$. Furthermore, this redistribution is such that their incomes are equalized, $w_{g}-t_{g}=s_{g}$, where $w_{g}$ is the wage rate, which implies that $t_{g}=(N_{g}-L_{g})w_{g}/N_{g}$, and thus, $w_{g}-t_{g}=L_{g}w_{g}/N_{g}$. That is, if there are more individuals unemployed, income per capita falls, and the opposite occurs if more people work. We also consider that consumers may pay lump-sum taxes $\tau _{g}$ that are directed to provide subsidies to firms. Therefore, letting $l_{g}$ be the fraction of workers in region *g* (i.e., $l_{g}=L_{g}/N_{g}$), the budget constraint—which is the same for all consumers—can be written as:

6 $$\begin{aligned} l_{g}w_{g}+\frac{{\mathcal {F}}_{g}+{\tilde{D}}_{g}}{N_{g}}-\tau _{g}=\sum _{j=1}^{J} \int _0^1 P_{g}^{j}(\Omega ^{j})c_{g}^{j}(\Omega ^{j})\ d\Omega ^{j}; \end{aligned}$$

where $P_{g}^{j}(\Omega ^{j})$ is the price of variety $\Omega ^{j}$ from sector-*j* consumed in *g*. The government of region *g* can also collect revenues from tariffs (${\mathcal {F}}_{g}$) that are redistributed to the whole local population. The term ${\tilde{D}}_{g}$ represents the regional trade deficit. Financing a trade deficit requires the inflow of resources from other locations, and this is why ${\tilde{D}}_{g}$ appears in the consumer’s budget constrain. Notice as well that this variable can be used in the experiments as a fiscal policy tool.

Given these preferences, the optimality conditions imply that the share of variety $\Omega ^{j}$ in consumption expenditure on the goods produced by industry *j* is a function of relative prices and the elasticity of substitution. In particular,

7 $$\begin{aligned} \frac{P_{g}^{j}(\Omega ^{j})c_{g}^{j}(\Omega ^{j})}{P_{g}^{j}c_{g}^{j}}=\left[ \frac{P_{g}^{j}(\Omega ^{j})}{P_{g}^{j}}\right] ^{1-\varsigma ^{j}}; \end{aligned}$$

where $P_{g}^{j}$ represents the ideal price index of the sector-*j* final products, which equals

8 $$\begin{aligned} P_{g}^{j}=\left[ \int _0^1 P_{g}^{j}(\Omega ^{j})^{1-\varsigma ^{j}}d\Omega ^{j}\right] ^{1/(1-\varsigma ^{j})}. \end{aligned}$$

These preferences also imply that consumption expenditure on sector *j* products in a location *g* is a constant fraction of total income given by $\alpha _{g}^{j}$.

Since income is fully spent in consumption goods, as shown by the budget constraint (6), we can express welfare from Eq. (4) through an indirect utility function as:

9 $$\begin{aligned} W_{g}=\frac{y_{g}}{P_{g}}; \end{aligned}$$

where $y_{g}$ is income per capita in region *g* given by

10 $$\begin{aligned} y_{g}=l_{g}w_{g}+\frac{{\mathcal {F}}_{g}+{\tilde{D}}_{g}}{N_{g}}-\tau _{g}; \end{aligned}$$

and $P_{g}$ provides the ideal consumption price index that households face in location *g*,

11 $$\begin{aligned} P_{g}=\prod _{j=1}^{J}\left( \frac{P_{g}^{j}}{\alpha _{g}^{j}}\right) ^{\alpha _{g}^{j}}. \end{aligned}$$

Note that welfare depends on the fraction of workers $l_{g}$, on the per capita trade deficit and tariff revenue. Thus, shocks to a sector affect welfare through the trade deficit, the tariff revenues and the price index. Furthermore, constraining the share of working individuals in a region has *ceteris paribus* first-order effects on welfare.^8^

#### Firms

In each location *g*, a firm that operates in sector *j* produces either an intermediate good variety ($q_{g}^{j}(\omega ^{j})$, $\omega ^{j}\in (0,1)$), a final product variety ($Q_{g}^{j}(\Omega ^{j})$, $\Omega ^{j}\in (0,1)$), or a composite intermediate or material ($Q_{g}^{{\mathcal {M}}j}$). The production of intermediate goods uses labor and materials from other industries, whereas the production process of final goods and materials demand intra-industry intermediates. Intermediate good manufacturers and final good and material producers in sector *j* may benefit from subsidization rates $s_{g}^{j}$ and ${\mathfrak {s}}_{g}^{j}$, respectively, which reduce the costs of the different production inputs in the same proportion. All markets are perfectly competitive and firms maximize profits. We next describe in more detail each of the different stages of the production chain.

#### Intermediate goods

A firm in sector *j* produces a variety $\omega ^{j}$ of intermediate goods using labor ($L_{g}^{j}(\omega ^{j})$) and composite intermediates from every other sector *k* ($m_{g}^{kj}(\omega ^{j})$) according to the production function:

12 $$\begin{aligned} q_{g}^{j}(\omega ^{j})=a_{g}^{j}\ z_{g}^{j}(\omega ^{j})L_{g}^{j}(\omega ^{j})^{\gamma _{g}^{j}}\prod _{k=1}^{J}m_{g}^{kj}(\omega ^{j})^{\gamma _{g}^{kj}}; \end{aligned}$$

where $a_{g}^{j}$ is sector *j*’s fundamental productivity in intermediate goods manufacturing by region *g*; $z_{g}^{j}(\omega ^{j})$ is a random sector-variety-specific productivity shock; and $\gamma _{g}^{j}$ denotes the share of value added on gross output. The term affected by the product operator provides the use of materials from all other sectors, with $\gamma _{g}^{kj}$ representing the expenditure share of the material from sector *k* employed in the input composite of the intermediate good produced by industry *j*. We assume that $\sum _{k=1}^{J}\gamma _{m}^{kj}=1-\gamma _{g}^{j}$. Production functions, then, exhibit constant returns to scale.

Because markets are perfectly competitive and firms are profit maximizers, intermediate good prices must equal marginal costs, $b_{g}^{j}/[a_{g}^{j} z_{g}^{j}(\omega ^{j})]$, where $b_{g}^{j}$ gives the cost of a unitary input bundle once subsidies are taken into account. The cost $b_{g}^{j}$ is common to all varieties and given by

13 $$\begin{aligned} b_{g}^{j}=(1-s_{g}^{j})\Upsilon _{g}^{j}w_{g}^{\gamma _{g}^{j}}\prod _{k=1}^{J}\left( P_{g}^{{\mathcal {M}}k}\right) ^{\gamma _{g}^{kj}}; \end{aligned}$$

where the constant $\Upsilon _{g}^{j}$ equals

$$\begin{aligned} \Upsilon _{g}^{j}=\left( \frac{1}{\gamma _{g}^{j}}\right) ^{\gamma _{g}^{j}}\prod _{k=1}^{J}\left( \gamma _{g}^{kj}\right) ^{-\gamma _{g}^{kj}}; \end{aligned}$$

$P_{g}^{{\mathcal {M}}k}$ is the price of the composite intermediate produced by sector *k* in region *g*; and $w_{g}$ denotes the wage rate in location *g*. Equation (13) says that the subsidy will translate into lower prices because it complements market revenues at paying for the inputs. Notice that the term $1-s_{g}^{j}$ can be written as a common factor because of constant returns to scale and because production subsidies reduce all input costs by the same proportion.

#### Final products

In each sector region (*j*, *g*) pair, a set of final goods indexed by $\Omega ^{j}$ are produced under perfect competition using intermediate goods from the same sector following a Dixit–Stiglitz aggregator with a constant elasticity of substitution $\sigma ^{j}>1$:

14 $$\begin{aligned} Q_{g}^{j}(\Omega ^{j})=A_{g}^{j}Z_{g}^{j}(\Omega ^{j})\left[ \int _0^1 r_{g}^{j}\left( \omega ^{j}\right) ^{1-1/\sigma ^{j}}d\omega ^{j}\right] ^{\frac{\sigma ^{j}}{\sigma ^{j}-1}}; \end{aligned}$$

where $A_{g}^{j}$ is the sector region fundamental productivity in final goods production; $r_{g}^{j}\left( \omega ^{j}\right)$ represents the demand in region *g* for intermediate good $\omega ^{j}$ from the lowest-cost supplier, which can belong to any of the regions.

Profit maximization implies the following demand function for each or the varieties:

15 $$\begin{aligned} r_{g}^{j}\left( \omega ^{j}\right) = \left[ \frac{(1-{\mathfrak {s}}_{g}^{j}) p_{g}^{j} \left( \omega ^{j}\right) }{B_{g}^{j}}\right] ^{-\sigma ^{j}}\frac{Q_{g}^{j}(\Omega ^{j})}{A_{g}^{j}Z_{g}^{j}(\Omega ^{j})}; \end{aligned}$$

where $p_{g}^{j}\left( \omega ^{j}\right)$ is the price of intermediate good $\omega ^{j}$ in location *g*, and $B_{g}^{j}$ gives the cost of the input bundle with subsidies already embedded as

16 $$\begin{aligned} B_{g}^{j}=(1-{\mathfrak {s}}_{g}^{j})\left[ \int _0^1 p_{g}^{j}\left( \omega ^{j}\right) ^{1-\sigma ^{j}}d\omega ^{j}\right] ^{\frac{1}{1-\sigma ^{j}}}. \end{aligned}$$

Equation (15) implies that the demand of intermediate $\omega ^{j}$ per unit of final output depends on the price of $\omega ^{j}$ relative to the price of the other varieties of intermediates. Consequently, as a response to the subsidy, the amount for intermediate products demanded can increase, not because of a change in the price that firms perceived $\left( (1-{\mathfrak {s}}_{g}^{j})\ p_{g}^{j}\left( \omega ^{j}\right) \right)$, but because of the decrease in the price of the final output (given by the marginal cost), which can cause an increase in $Q_{g}^{j}(\Omega ^{j})$.

#### Composite intermediate goods

Production of materials in sector *j* uses a very similar technology to the one of final goods. In particular,

17 $$\begin{aligned} Q_{g}^{{\mathcal {M}}j}=A_{g}^{j}\left[ \int _0^1 r_{g}^{j}(\omega ^{j})^{1-1/\sigma ^{j}}d\omega ^{j}\right] ^{\frac{\sigma ^{j}}{\sigma ^{j}-1}}. \end{aligned}$$

That is, it also combines varieties of intermediate goods coming from the same sector. The difference with Eq. (14) is that productivity in the case of the production of the composite intermediate is fully deterministic. The demand for intermediate inputs is analogous to the one delivered by final goods; in particular,

18 $$\begin{aligned} r_{g}^{j}\left( \omega ^{j}\right) = \left[ \frac{(1-{\mathfrak {s}}_{g}^{j}) p_{g}^{j}\left( \omega ^{j}\right) }{B_{g}^{j}}\right] ^{-\sigma ^{j}}\frac{Q_{g}^{{\mathcal {M}}j}}{A_{g}^{j}}. \end{aligned}$$

Because composite intermediate goods do not engage in inter-regional trade, the price paid for them by intermediate goods manufacturers is directly given by the marginal cost of production in the same location. This implies that

19 $$\begin{aligned} P_{g}^{{\mathcal {M}}j}=\frac{B_{g}^{j}}{A_{g}^{j}} \end{aligned}$$

which is the ratio between the cost of the input bundle accounting for subsidies divided by the productivity of the composite intermediate goods producers.

#### Inter-regional trade and destination prices

Intermediate goods and final products can travel across locations. Inter-regional trade is costly. Trade costs combine tariffs and iceberg transportations costs. We consider that tariff may be different for intermediate and final goods. More specifically, a sector-*j* intermediate imported by region *g* from location *i* involves a trade cost equal to

20 $$\begin{aligned} \kappa _{gi}^{j}=\left( 1 + \tau _{gi}^{j}\right) d_{gi}^{j}; \end{aligned}$$

where $\tau _{gi}^{j}$ is the imposed ad valorem tariff on intermediate goods from sector *j*. The transportation cost $d_{gi}^{j}$ implies that the arrival of one unit of an intermediate product to *g* coming from *i* requires sending $d_{gi}^{j}$ units produced of that product. For the case of final goods, trade costs equal

21 $$\begin{aligned} K_{gi}^{j}=\left( 1+T_{gi}^{j}\right) {\mathfrak {d}}_{gi}^{j}. \end{aligned}$$

Now, $T_{gi}^{j}$ represents the tariff on final goods from industry *j*, and ${\mathfrak {d}}_{gi}^{j}$ the iceberg costs related to trade in final goods. Because we will use changes in iceberg costs as proxies to study the effect of supply-chain disruptions, it is only assumed that $d_{gi}^{j},{\mathfrak {d}}_{gi}^{j}\ge 1$ for all *g* and *i*. For the same reason, the usual triangular inequality $\kappa _{gi}^{j}\le \kappa _{hi}^{j}\kappa _{gh}^{j}$ and $K_{gi}^{j}\le K_{hi}^{j}K_{gh}^{j}$ may not hold for all *g*, *i* and *h*.

Taking into account these trade costs, the prices at destination of the traded products from the lowest-cost supplier are given by:

22 $$\begin{aligned} p_{g}^{j}(\omega ^{j})=\min _{i\in [1,G]}\left\{ \frac{b_{i}^{j}\kappa _{gi}^{j}}{a_{g}^{j}z_{g}^{j}(\omega ^{j})}\right\} \end{aligned}$$

and

23 $$\begin{aligned} P_{g}^{j}(\Omega ^{j})=\min _{i\in [1,G]}\left\{ \frac{B_{i}^{j}K_{gi}^{j}}{A_{g}^{j}Z_{g}^{j}(\Omega ^{j})}\right\} . \end{aligned}$$

Equations (22) and (23) imply that the price at destination will be given by the minimum across locations of the product between the marginal cost and the trade cost. A more expensive input bundle or higher trade costs will push the price up, whereas a larger productivity will push it down.

Following Eaton and Kortum (2002), trade in the model obeys a Ricardian motive generated by a random allocation of productivities across sectors and regions. In particular, the realizations of the productivity variables $z_{g}^{j}$ and $Z_{g}^{j}$ for varieties $\omega ^{j}$ and $\Omega ^{j}$ follow Fréchet distributions with location parameter equal to 1 and sector-specific shape parameters $\theta ^{j}$ and $\Theta ^{j}$, respectively. A smaller value of the shape parameter implies a larger dispersion of the distribution. We assume that the random productivity variables are independently distributed across goods, industries, and regions. Furthermore, we impose that $1+\theta ^{j}>\sigma ^{j}$ and $1+\Theta ^{j}>\varsigma ^{j}$. Following Caliendo and Parro (2015), under these assumptions for the distributions of productivities, we can rewrite Eqs. (16) and (8) as

24 $$\begin{aligned} B_{g}^{j}= &  (1-{\mathfrak {s}}_{g}^{j})\ \Gamma \left( 1+\frac{1-\sigma ^{j}}{\theta ^{j}}\right) ^{1/(1-\sigma ^{j})}\left[ \sum _{i = 1}^G \left( \frac{b_{i}^{j}\kappa _{gi}^{j}}{a_{i}^{j}}\right) ^{-\theta ^{j}}\right] ^{-1/\theta ^{j}}, \end{aligned}$$

25 $$\begin{aligned} P_{g}^{j}= &  \Gamma \left( 1+\frac{1-\varsigma ^{j}}{\Theta ^{j}}\right) ^{1/(1-\varsigma ^{j})}\left[ \sum _{i=1}^{G} \left( \frac{B_{i}^{j}K_{gi}^{j}}{A_{i}^{j}}\right) ^{-\Theta ^{j}}\right] ^{-1/\Theta ^{j}}; \end{aligned}$$

where $\Gamma (\cdot )$ is the gamma function.

In the case that a sector is not tradable, which implies that all the varieties of intermediate goods and consumption products from that sector are bought from domestic producers, Caliendo and Parro (2015) also show that the relevant price indices amount to imposing that $\kappa _{gi}^{j}=K_{gi}^{j}=\infty$ for all $i\ne g$ in Eqs. (24) and (25). Then, we can express $B_g^j$ and $P_g^j$ as

$$\begin{aligned} B_{g}^{j}=(1-{\mathfrak {s}}_{g}^{j})\ \Gamma \left( 1 + \frac{1-\sigma ^{j}}{\theta ^{j}}\right) ^{1/(1-\sigma ^{j})} \frac{b_{g}^{j}}{a_{g}^{j}} \end{aligned}$$

and

$$\begin{aligned} P_{g}^{j}=\Gamma \left( 1 + \frac{1-\varsigma ^{j}}{\Theta ^{j}}\right) ^{1/(1-\varsigma ^{j})} \left( \frac{B_{g}^{j}}{A_{g}^{j}}\right) . \end{aligned}$$

#### Expenditure shares

Let $x_{g}^{j}$ and $X_{g}^{j}$ be region *g*’s total expenditures on intermediate goods and final products from sector *j*, respectively. They are obtained at destination prices, and therefore, include tariff payments. Define $x_{gi}^{j}$ and $X_{gi}^{j}$ as the expenditures in location *g* on sector-*j* intermediate goods and sector-*j* final products, respectively, imported by location *g* from location *i*. Finally, let $\pi _{gi}^{j}$ and $\Pi _{gi}^{j}$ be region *g*’s total expenditure shares of intermediate goods and final products from sector *j* exported by location *i* to location *g*, respectively, that is, $\pi _{gi}^{j}=x_{gi}^{j}/x_{g}^{j}$ and $\Pi _{gi}^{j}=X_{gi}^{j}/X_{g}^{j}$. Following Caliendo and Parro (2015), it can be shown that

26 $$\begin{aligned} \pi _{gi}^{j}=\frac{\left( b_{i}^{j}\kappa _{gi}^{j}/a_{i}^{j}\right) ^{-\theta ^{j}}}{\sum _{h=1}^{G} \left( b_{h}^{j}\kappa _{gh}^{j}/a_{h}^{j}\right) ^{-\theta ^{j}}}, \end{aligned}$$

27 $$\begin{aligned} \Pi _{gi}^{j}=\frac{\left( B_{i}^{j}K_{gi}^{j}/A_{i}^{j}\right) ^{-\Theta ^{j}}}{\sum _{h=1}^{G} \left( B_{h}^{j}K_{gh}^{j}/A_{h}^{j}\right) ^{-\Theta ^{j}}}. \end{aligned}$$

Bilateral trade shares contain important information. First, they are declining on transport costs and increasing in the productivity of the producer (since this productivity reduces the marginal cost directly). Second, they include information on the input–output structure of the whole economy through the prices paid for intermediate inputs. Furthermore, this input–output structure is also affected by the economic geography, since intermediate inputs can be imported from abroad. In terms of the effects of policies regarding the control of COVID-19, this gravity equation is potentially informative for several reasons. It can potentially capture the fact that some sectors might be more affected by social distancing policies, since sectors can differ in their labor input intensities. Dingel and Neiman (2020) estimate that, in the USA, the share of jobs that can be done from home significantly varies across cities and industries and also show that this share is decreasing in the level of development of the countries. Our model could plausibly capture this. Our model could also show the effects of how shutting down a certain sector or region would affect the rest of sectors and locations through the input–output structure. Furthermore, in the second phase of the model, infections can be spread through economic linkages; since some sectors are more interconnected than others, those regions that are more intensive in certain inputs can show significantly faster infection rates.

#### Market clearing and government and regional deficits

Local labor markets require that the sum of labor employed in the different industries equals the total amount of labor available in the region. Formally,

28 $$\begin{aligned} \sum _{j=1}^{J}L_{g}^{j}=L_{g} \end{aligned}$$

Furthermore, because in equilibrium labor costs must equal a constant fraction $\gamma _{g}^{j}$ of the value of the intermediate goods production, the following condition must hold:

29 $$\begin{aligned} w_{g}L_{g}= \sum _{j=1}^{J} \frac{\gamma _{g}^{j}}{1-s_{g}^{j}}\sum _{i=1}^{G} \frac{x_{i}^{j}\pi _{ig}^{j}}{1+\tau _{ig}^{j}}. \end{aligned}$$

Notice that the right-hand side of Eq. (29) adds the expenditures across sectors and regions on intermediate goods manufactured in location *g* that go to pay the labor input. It also implies that payments to labor are in part satisfied using the subsidies, in an amount equivalent to a fraction $\gamma _{g}^{j}s_{g}^{j}/(1-s_{g}^{j})$ of the revenues from sales. We divide by the tariff to convert each expenditure amount into the value of production.

In the same manner, the total value of the production of composite intermediates from sector *j* in a location *g* has to be equal to a subsidy-weighted fraction (determined by all $\gamma _{g}^{jk}$) of the expenditure on region *g*’s intermediate goods across sectors and locations. In particular,

30 $$\begin{aligned} P_{g}^{{\mathcal {M}}j}Q_{g}^{{\mathcal {M}}j}= \sum _{k=1}^{J} \frac{\gamma _{g}^{jk}}{1-s_{g}^{j}} \sum _{i=1}^{G} \frac{x_{i}^{k}\pi _{ig}^{k}}{1+\tau _{ig}^{k}}. \end{aligned}$$

Notice that market clearing conditions (29) and (30) imply as well that the intermediate goods market clears.

Employing again a production expenditure equality, market clearing in the location *g*’s final goods market requires that the value of the sector-*j* final goods produced in *g* equals the consumption expenditure across regions on final products from that location. Taking into account that the revenues from the production activity of the final product sector fully goes to pay for the intermediate goods used as inputs, we can write the market clearing condition as:

31 $$\begin{aligned} x_{g}^{j}-\frac{P_{g}^{{\mathcal {M}}j}Q_{g}^{{\mathcal {M}}j}}{1-{\mathfrak {s}}_{g}^{k}} = \frac{1}{1-{\mathfrak {s}}_{g}^{k}} \sum _{i=1}^{G} \frac{X_{i}^{j}\Pi _{ig}^{j}}{1+T_{ig}^{j}}. \end{aligned}$$

The left-hand side of Eq. (31) subtracts the value of materials to provide just the amount of expenditure in intermediate goods satisfied by final goods producers. The subsidy ${\mathfrak {s}}_{g}^{k}$ is in the equation because the expenditure on inputs, $x_{g}^{j}$, equals the market revenues—given by the terms affected by the sum operator—plus the subsidies received by the industry.

Note that consumers’ expenditure on sector-*j* products in region-*i* is a fixed fraction $\alpha _{i}^{j}$ of their income. Hence,

32 $$\begin{aligned} X_{i}^{j}=\alpha _{i}^{j}y_{i}N_{i}; \end{aligned}$$

where income per capita $y_{i}$, given by Eq. (10), is a function of tariff revenues. We can now express tariff revenues, ${\mathcal {F}}_g$, using the notation introduced previously as:

33 $$\begin{aligned} {\mathcal {F}}_{g} = \sum _{j=1}^{J} \sum _{i=1}^{G} \left( \tau _{gi}^{j}\frac{x_{g}^{j}\pi _{gi}^{j}}{1+\tau _{gi}^{j}}+T_{gi}^{j}\frac{X_{g}^{j}\Pi _{gi}^{j}}{1+T_{gi}^{j}}\right) . \end{aligned}$$

Moving next to the determination of the trade balance, we consider that the regional trade deficit ${\tilde{D}}_{g}$ is given by the sum of the sectoral deficits, ${\tilde{D}}_{g}^{j}$. The sectoral deficit ${\tilde{D}}_{g}^{j}$ equals the value of the region *g*’s imports of industry-*j* goods from all other locations minus the value of exports of sector-*j* products from location *g* to all other locations. This is equivalent to imposing that the deficit is given by the difference between total expenditure by region *g* on sector-*j* intermediate and final products net of tariffs and the total value of production of industry-*j* intermediate and final goods in location *g*. More specifically,

34 $$\begin{aligned} {\tilde{D}}_{g}^{j} = \sum _{i=1}^{G} \left( \frac{x_{g}^{j}\pi _{gi}^{j}}{1+\tau _{gi}^{j}}+\frac{X_{g}^{j}\Pi _{gi}^{j}}{1+T_{gi}^{j}}\right) - \sum _{i=1}^{G} \left( \frac{x_{i}^{j}\pi _{ig}^{j}}{1+\tau _{ig}^{j}}+\frac{X_{i}^{j}\Pi _{ig}^{j}}{1+T_{ig}^{j}}\right) . \end{aligned}$$

The second parenthesis gives the value of production by adding across locations the amount spent on products from the sector region pair (*j*, *g*) net of tariffs.

Therefore, trade balance in location *g* implies the sum of the sectoral trade deficits must equal the regional one, which implies

35 $$\begin{aligned} {\tilde{D}}_{g} = \sum _{j=1}^{J} {\tilde{D}}_{g}^{j}. \end{aligned}$$

It can be shown that the trade balance condition, equation (35), implies that the labor market clears, that is, equation (29).

Finally, we allow for the possibility that the regional budget deficit, denoted by ${\bar{D}}_{g}$, is not zero. Therefore, the following condition must hold:

36 $$\begin{aligned} {\bar{D}}_{g} = \sum _{j=1}^{J} \sum _{i=1}^{G} \left( \frac{s_{g}^{j}}{1-s_{g}^{j}}\frac{x_{i}^{j}\pi _{ig}^{j}}{1 + \tau _{ig}^{j}} + \frac{{\mathfrak {s}}_{g}^{j}}{1 - {\mathfrak {s}}_{g}^{j}} \frac{X_{i}^{j} \Pi _{ig}^{j}}{1 + T_{ig}^{j}}\right) + \sum _{j=1}^{J} \frac{{\mathfrak {s}}_{g}^{j}}{1 - {\mathfrak {s}}_{g}^{j}} P_{g}^{{\mathcal {M}}j}Q_{g}^{{\mathcal {M}}j} - \tau _{g}N_{g}. \nonumber \\ \end{aligned}$$

That is, if the expenditure in subsidies is larger than the taxes collected to finance them, there will be a positive budget deficit.

#### Equilibrium system in relative changes

As in Caliendo and Parro (2015), we solve the model in changes.^9^ Let us denote a proportional change in a variable with a hat (  ) and the value of the variable next period with a prime ($^{\prime }$). Then, for example, ${\hat{\tau }}_{gi}^{j}=\tau _{gi}^{j\prime }/\tau _{gi}^{j}$. The exogenous shocks that we will consider correspond to new tariffs, $\tau _{gi}^{j\prime }$ and $T_{gi}^{j\prime }$, new subsidies to firms, $s_{g}^{j\prime }$ and ${\mathfrak {s}}_{g}^{j\prime }$, supply-chain disruptions proxied by changes in the trade costs, ${\hat{d}}_{gi}^{j}$ and $\mathfrak {{\hat{d}}}_{gi}^{j}$ for $g\ne i$, local production restrictions proxied by ${\hat{d}}_{gg}^{j}$ and $\mathfrak {{\hat{d}}}_{gg}^{j}$, and confinement policies captured by new stocks of available labor in the region, $L_{g}^{\prime }$.

Equations (13) and (19) imply that the gross growth rate in the cost of the intermediate goods input bundle equals

37 $$\begin{aligned} {\hat{b}}_{g}^{j}=\left( \frac{1-s_{g}^{j\prime }}{1-s_{g}^{j}}\right) {\hat{w}}_{g}^{\gamma _{g}^{j}}\prod _{k=1}^{J} \left( {\hat{B}}_{g}^{k}\right) ^{\gamma _{g}^{kj}}. \end{aligned}$$

In turn, combining expressions (24) and (26) obtains the change in the cost of the final goods input bundle and the export shares of intermediate products as

38 $$\begin{aligned} {\hat{B}}_{g}^{j} = \left( \frac{1-{\mathfrak {s}}_{g}^{j\prime }}{1-{\mathfrak {s}}_{g}^{j}}\right) \left[ \sum _{i=1}^{G} \pi _{gi}^{j} \left( {\hat{b}}_{i}^{j} {\hat{\kappa }}_{gi}^{j}\right) ^{-\theta ^{j}}\right] ^{-1/\theta ^{j}} \end{aligned}$$

and

39 $$\begin{aligned} {\hat{\pi }}_{gi}^{j} = \left( \frac{{\hat{b}}_{i}^{j}{\hat{\kappa }}_{gi}^{j}}{{\hat{B}}_{g}^{j}}\right) ^{-\theta ^{j}}, \end{aligned}$$

respectively, where ${\hat{\kappa }}_{gi}^{j} = \left( 1 + \tau _{gi}^{j\prime }\right) {\hat{d}}_{gi}^{j}/\left( 1 + \tau _{gi}^{j}\right)$.The gross growth rate in the sectoral price index and the final good export shares are obtained from Eqs. (25) and (27) as

40 $$\begin{aligned} {\hat{P}}_{g}^{j} = \left[ \sum _{i=1}^{G} \Pi _{gi}^{j} \left( {\hat{B}}_{i}^{j}{\hat{K}}_{gi}^{j}\right) ^{-\Theta ^{j}}\right] ^{-1/\Theta ^{j}} \end{aligned}$$

and

41 $$\begin{aligned} {\hat{\Pi }}_{gi}^{j}=\left( \frac{{\hat{B}}_{i}^{j}{\hat{K}}_{gi}^{j}}{{\hat{P}}_{g}^{j}}\right) ^{-\Theta ^{j}}, \end{aligned}$$

respectively, where $${\hat{K}}_{gi}^{j} = \left( 1 + T_{gi}^{j\prime }\right) \mathfrak {{\hat{d}}}_{gi}^{j}/\left( 1 + T_{gi}^{j}\right) $$.

Market clearing conditions can be employed to obtain the future values of the expenditure variables as a function of the above changes. In particular, market clearing for final goods, equations (30) and (31), implies that region *g*’s next-period expenditure in intermediate goods from sector *j* is given by:

42 $$\begin{aligned} x_{g}^{j\prime }=\frac{1}{1-{\mathfrak {s}}_{g}^{j\prime }}\left( \sum _{k=1}^{J} \sum _{i=1}^{G} \frac{\gamma _{g}^{jk}}{1 - s_{g}^{j\prime }} \frac{x_{i}^{k\prime } \pi _{ig}^{k\prime }}{1 + \tau _{ig}^{k\prime }} + \sum _{i=1}^{G} X_{i}^{j\prime } \frac{\Pi _{ig}^{j\prime }}{1 + T_{ig}^{j\prime }}\right) . \end{aligned}$$

Notice that $\pi _{ig}^{k\prime }$ and $\Pi _{ig}^{j\prime }$ can be written as $\pi _{ig}^{k}{\hat{\pi }}_{ig}^{k}$ and $\Pi _{ig}^{j}{\hat{\Pi }}_{ig}^{j}$, respectively.

From Eqs. (10), (30), (32), (33), and (36), next-period’s expenditure in final goods from sector *j* is given by

43 $$\begin{aligned} X_{g}^{j\prime } = \alpha _{g}^{j} \left[ L_{g}^{\prime }w_{g}^{\prime } + \sum _{k=1}^{J} \sum _{i=1}^{G} \left( \tau _{gi}^{k\prime } \frac{x_{g}^{k\prime } \pi _{gi}^{k\prime }}{1 + \tau _{gi}^{k\prime }} + T_{gi}^{k\prime } \frac{X_{g}^{k\prime } \Pi _{gi}^{k\prime }}{1 + T_{gi}^{k\prime }}\right) + {\tilde{D}}_{g}^{\prime } - \tau _{g}^{\prime } N_{g}\right] ; \end{aligned}$$

where

44 $$\begin{aligned} {\tilde{D}}_{g}^{\prime } = \sum _{j=1}^{J} \sum _{i=1}^{G} \left( \frac{x_{g}^{j\prime } \pi _{gi}^{j\prime }}{1 + \tau _{gi}^{j\prime }} + \frac{X_{g}^{j\prime } \Pi _{gi}^{j\prime }}{1 + T_{gi}^{j\prime }}\right) - \sum _{j = 1}^J \sum _{i = 1}^G \left( \frac{x_{i}^{j\prime } \pi _{ig}^{j\prime }}{1 + \tau _{ig}^{j\prime }} + \frac{X_{i}^{j\prime } \Pi _{ig}^{j\prime }}{1 + T_{ig}^{j\prime }}\right) . \end{aligned}$$

Again, we can write $w_{g}^{\prime }$ as $w_{g}{\hat{w}}_{g}$ so that it becomes a function of the changes determined by previous equations in the system.

The system formed by Eqs. (37)–(44) is underdetermined because the number of unknowns is equal to the number of equations plus one. In order to solve it, Caliendo and Parro (2015) assume that the economy’s trade deficit in each location *g* is exogenous. We, on the other hand, allow for the trade deficit to be determined by the model and, instead, required that the wage rate does not vary. This looks to us more appropriate for the problem that we analyze.

Equations (37)–(44) imply that we do not need to calibrate fundamental productivities and trade costs to solve the system. We simply start from a baseline scenario that consists of initial data on regional wages, labor, and trade and budget deficits $\{w_{g},L_{g},\tilde{D}_{g},{\bar{D}}_{g}\}_{g=1}^{G}$, pairwise regional expenditure shares and tariffs in every sector $\{\pi _{gi}^{j},\Pi _{gi}^{j},\tau _{gi}^{j},T_{gi}^{j}\}_{g=1,i=1,j=1}^{G,G,J}$, and the assumption of no subsidies for firms, $s_{g}^{j} = {\mathfrak {s}}_{g}^{j} = 0$. We also need to assign values to the labor share in gross output ($\gamma _{g}^{j}$), the share of intermediate goods from sector *k* employed in the production of sector *j* ($\gamma _{g}^{jk}$), the share of consumption expenditure on sector-*j* goods ($\alpha _{g}^{j}$), and the shape parameters $\theta ^{j}$ and $\Theta ^{j}$ of the Fréchet distributions. With that information on our hands, we consider shocks on the values $\tau _{gi}^{\prime }$, $T_{gi}^{\prime }$, $s_{g}^{j\prime }$, ${\mathfrak {s}}_{g}^{j\prime }$, ${\hat{d}}_{gi}^{j}$, $\mathfrak {{\hat{d}}}_{gi}^{j}$ and/or $L_{g}^{\prime }$, and solve the system going through the following steps.

1. Assume ${\hat{w}}_{g}=0$ for all *g*.
2. From Eqs. (37) and (38), obtain $\{{\hat{b}}_{g}^{j},{\hat{B}}_{g}^{j}\}_{g=1,j=1}^{G,J}$.
3. Once we know the cost of the unitary input bundles, we can recover from Eqs. (40)–(41) the values of $\{{\hat{P}}_{g}^{j},{\hat{\pi }}_{gi}^{j},{\hat{\Pi }}_{gi}^{j}\}_{g=1,i=1,j=1}^{G,G,J}$.
4. Obtain $\{x_{g}^{j\prime },X_{g}^{j\prime }\}_{g=1,j=1}^{G,J}$ using (42) and (43).

The above implies that in this economy, an equilibrium in relative changes can be defined as follows. Given the new value of the regional labor supply $\{L_{g}\}_{g=1}^{G}$, regional deficits $\{{\tilde{D}}_{g}^{\prime },{\bar{D}}_{g}^{\prime }\}_{g=1}^{G}$, and pairwise regional government tariffs on intermediate goods $\{\tau _{gi}^{j\prime }\}_{g=1,j=1}^{G,J}$ and on final goods $\{T_{gi}^{j\prime }\}_{g=1,j=1}^{G,J}$, a competitive equilibrium is a set of changes in intermediate good and final product price indices in for each sector location pair $\{{\hat{B}}_{g}^{j},{\hat{P}}_{g}^{j}\}_{g=1,j=1}^{G,J}$, and pairwise regional expenditure shares in every sector $\{{\hat{\pi }}_{gi}^{j},{\hat{\Pi }}_{gi}^{j}\}_{g=1,i=1,j=1}^{G,G,J}$, in addition to new values of the total sector location expenditure volumes $\{x_{g}^{j\prime },X_{g}^{j\prime }\}_{g=1,j=1}^{G,J}$, such that the optimizing conditions for households, intermediate product manufacturers, final good firms and material producers—which are reflected in Eqs. (13), (19), (24) to (27), and (32)—hold, and market clearing in all markets is achieved through conditions (30), (31) and (34).

### Phase 2: infection dynamics

The infection dynamics take place at the local level but we allow for possible contagions across locations depending on effective distance. Typically, epidemiology models characterize the transitions from one state to another with exogenously given probabilities that refer to the characteristics of the particular infection. Here, instead, we assume that transition probabilities depend on two factors, one exogenous that captures the characteristics of the infection, and an endogenous geographic component that captures how more economically active locations can be more prone to infections since they have more connections with the rest of locations.

People that work face-to-face, people that work telematically, and people that do not work have different probabilities of catching the disease due to their different number of encounters with other people. Additionally, individuals that have recovered from the disease or have been vaccinated can have a lower probability of becoming infected. We assume that all the infected, regardless of whether they are in hospital or not, are able to pass the disease to workers.

We consider two scenarios where people can become infected. Firstly, infection transmission can occur locally, between residents of a given region, through non-work-related *social* interactions, (e.g., visiting relatives, walking in public spaces, or when final consumers purchase goods). Secondly, the virus can be transmitted through *work-related* activities, which we refer to as the *geographic component*. This includes interactions such as workers in a factory producing output, interactions between workers from different firms or locations, and interactions between workers and final consumers. Within this second component, consistent with the evidence reviewed in Sect. 2, the movement of goods and services within and between regions represents an important vector for the transmission of the disease because some degree of human interaction always occurs during those transactions. For example, this can happen when infected tourists come into contact with service providers or via infected truck drivers who transport goods.^10^

Hence, the dynamics for infected people can be written as:

45 $$\begin{aligned} I_{g}^{\prime }=\underset{\text { Infected not becoming resolving}}{\underbrace{(1-\varphi )I_{g}}} + S_{g}\Phi _{g}; \end{aligned}$$

where the term $\Phi _{g}$ is given by

46 $$\begin{aligned} \Phi _{g} = \underset{\text {Social Component}}{\underbrace{(1 - \kappa )\rho _{g}\frac{I_{g}}{N_{g}}}} + \underset{\text {Geographic Component}}{\underbrace{\kappa \left( \sum _{i=1}^{G} \rho _{i} \frac{I_{i}}{N_{i}} \Lambda _{i} {\tilde{X}}_{ig}\right) }}; \end{aligned}$$

the coefficient $\varphi$ gives the fraction of infected that become resolving every period; $\kappa$ captures the proportion of infections that arise in work-related contexts and is time-invariant. The time-varying probability $\rho _{g}$ provides the likelihood that a susceptible individual contract the disease upon encountering an infected person. We allow this parameter to be affected by local policies and local behaviors. Parameter $\Lambda _{i}$ controls for the degree of telematic work in region *i*, which reduces contact among people at the workplace. Finally, ${\tilde{X}}_{ig}$ represents the relative level of bilateral transactions between *i* and *g*.

We assume that this relative level of bilateral transactions between two regions is given by:

47 $$\begin{aligned} {\tilde{X}}_{ig} = \frac{\sum _{j=1}^{J} \left( x_{ig}^{j} + x_{gi}^{j} + X_{ig} ^{j} + X_{gi}^{j} \right) }{\sum _{h=1}^{G}\sum _{k=1}^{J} \left( x_{hg}^{k} + x_{gh}^{k} + X_{hg}^{k} + X_{gh}^{k}\right) } \end{aligned}$$

Equation (47) aims to capture the level of market-related human interactions between two economies *i* and *g* as a function of bilateral imports and exports when two different locations trade, or as a function of the local expenditure volumes if market activity is fully local.^11^

Therefore, according to motion Eq. (45), the number of infected people tomorrow depends on infected people today net of those that become resolving cases. The equation also considers that the susceptible can catch the disease. As expression (46) specifies, this can occur through the social and the geographic components. The strength of the social component in region *g* depends on the weight of non-work-related interactions $(1-\kappa )$, the relative prevalence of the disease $(I_{g}/N_{g})$, and the contagion probability $(\rho _g)$, all of them referred to region *g*. However, the strength of the geographic component depends on variables related to the trade partner *i*. Here, we are assuming that the probability of disease transmission is primarily determined by the policies that affect the infected individual and her habits. Specifically, the strength of the geographic component depends on the proportion of work-related infections, disease prevalence, the contagion probability, the degree of telematic work ($\Lambda _{i}$) in region *i*, and on the relative level of bilateral transactions between *i* and *g* (${\tilde{X}}_{ig}$).^12^

The following equations, along with Eq. (45), describe the full epidemiological model:

48a $$\begin{aligned} S_{g}^{\prime }&=(1-\lambda _{g}-\Phi _{g})S_{g}+\alpha ^{V}V_{g}+\alpha ^{C}C_{g} \end{aligned}$$

48b $$\begin{aligned} V_{g}^{\prime }&=(1-\alpha ^{V})V_{g}+\lambda _{g}S_{g} \end{aligned}$$

48c $$\begin{aligned} R_{g}^{\prime }&=\varphi I_{g}+(1-\xi )R_{g} \end{aligned}$$

48d $$\begin{aligned} C_{g}^{\prime }&=(1-\alpha ^{C})C_{g}+(1-\delta )\xi R_{g} \end{aligned}$$

48e $$\begin{aligned} F_{g}^{\prime }&=F_{g}+\delta \xi R_{g} \end{aligned}$$

48f $$\begin{aligned} N_{g}^{\prime }&=N_{g}-\delta \xi R_{g} \end{aligned}$$

Parameter $\lambda _{g}$ provides the fraction of the susceptible that are vaccinated during the period in location *g*; $\alpha _{c}$ and $\alpha _{v}$ are the fractions of the recovered and the vaccinated that fully lose immunity, respectively; the parameter $\xi$ reflects the fraction of cases that resolve in a given period, and therefore, its inverse pins down the average number of periods it takes for a case to resolve; and $\varphi$ relates to the average number of days (given by $1/\varphi$) a person is infectious.

Equation (48a) says that the size of the susceptible population decreases with the fraction $\lambda _{g}$ that receives the vaccine and the fraction $\Phi _{g}$ that gets infected by the COVID-19 virus, but rises with the recovered and vaccinated that lose their immunity. The vaccinated population, equation (48b), increases with the fraction of the susceptible that receive the vaccine and decreases with the vaccinated individuals that lose immunity. In Eq. (48c), in turn, a fraction $\varphi$ of infected individuals become resolving, and a fraction $\xi$ of cases is resolved. The number of recovered individuals, as given by Eq. (48d), evolves in a similar way to that of the vaccinated. A fraction $\alpha _{c}$ loses their immunity and some of the resolving, among the fraction $\delta$ that survive, recover during the period. Regarding the evolution of the stock of fatalities ($F_{g}$), according to Eq. (48e), the new deaths come from the fraction $(\delta \xi )$ of resolving that resolve and die. Finally, the evolution of the region’s population is given by Eq. (48f), which implies that a fraction $(\delta \xi )$ of the resolving cases die.

## Calibration

The main source for the calibration of the economic part of the model is Thiessen (2020), which offers the Rhomolo-MRIO Tables for 2013 published by the European commission. The dataset provides input–output tables for a set of 268 regions that include 267 EU28 NUTS2-2010 areas plus the rest of the world (ROW). Nevertheless, due to the lack of sufficiently disaggregated data for the disease variables, we need to aggregate some locations to the NUTS1 and country levels. After doing so, we are left with 230 regions (see Table 1). The numbers are disaggregated into ten main sectors of activity belonging to the NACE Rev2 classification (see Table 4). A summary of the data sources employed for the calibration of both the economic and disease parameters—and in some cases their values—are provided in Table 5.

**Table 4:** NACE Rev2 sectors included in the analysis

Section	Industry
A	Agriculture, forestry and fishing
B_E	Industry (except construction and mining)
C	Mining
F	Construction
G_I	Wholesale and retail trade, transport, accommodation and food service activities
J	Information and communication
K_L	Financial, insurance, and real estate activities
M_N	Professional, scientific, technical, administrative and support service activities
O_Q	Public administration, defense, education, human health and social work activities
R_U	Arts, entertainment and recreation; other service activities; activities of household and extra-territorial organizations and bodies

**Table 5:** Death and infection data sources by country

Country	Country code	Number of regions	Indicator*	Source
Austria	AT	9	Deaths	AGES
Belgium	BE	3	Deaths	Sciensano
Bulgaria	BG	1	Deaths	Our World In Data
Croatia	HR	1	Deaths	Our World In Data
Cyprus	CY	1	Deaths	Our World In Data
Czech Republic	CZ	8	Deaths	Ministry of Health
Denmark	DK	5	Infections	Statens Serum Institut
Estonia	EE	1	Deaths	Our World In Data
Finland	FI	5	Deaths	Helsing Sanomat
France	FR	22	Deaths	Government Statistical Office
Germany	DE	38	Deaths	Robert Koch Institute
Greece	EL	13	Infections	Ministry of Health
Hungary	HU	1	Deaths	Our World In Data
Ireland	IE	1	Deaths	Our World In Data
Italy	IT	21	Deaths	Dipartimento della Protezion Civile
Latvia	LV	1	Deaths	Our World In Data
Lithuania	LT	1	Deaths	Our World In Data
Luxembourg	LU	1	Deaths	Our World In Data
Malta	MT	1	Deaths	Our World In Data
Netherlands	NL	1	Deaths	Our World In Data
Poland	PL	16	Deaths	Government of Poland
Portugal	PT	7	Deaths	Ministry of Health
Rest of the World	ROW	1	Infections	Our World In Data
Romania	RO	2	Deaths	Our World In Data
Slovakia	SK	4	Infections	Radovan Ondas**
Slovenia	SI	2	Deaths	COVID-19 Sledilnik
Spain	ES	19	Deaths	Narrativa Tracking
Sweden	SE	8	Deaths	Public Health Agency of Sweden
United Kingdom	UK	37	Infections	National Health Service

*Population numbers at the time when the pandemic started come from the same sources

**Radovan Ondas independently compiled a machine readable dataset from the reports published by the National Health Information Centre. The data are accessible in his GitHub Repository: https://github.com/radoondas/covid-19-slovakia/

**Table 6:** Calibration summary

Parameter	Source	Value | Description
$\alpha ^j_g$	Thissen et al. (2019)	Share of sector *j* in total consumption expenditure in location *g*
$\gamma ^j_g$	Thissen et al. (2019)	Share of value added in gross output
$\gamma ^{kj}_g$	Thissen et al. (2019)	Input–output coefficients
$\theta ^j, \Theta ^j$	Thissen et al. (2019) and Persyn et al. (2020)	Gravity equation estimation
$\Lambda _g$	Dingel and Neiman (2020)	Estimated using data on who can work from home and trade shares
$\kappa$	Eichenbaum et al. (2021)	0.17 | Average infection rate related to work
$\phi$	Fernández-Villaverde and Jones (2022)	0.125 | Average infections per period. Then $1/\phi = 8$ days
$\xi$	Fernández-Villaverde and Jones (2022)	0.143 | Average number of days to resolve. Then, $1/\xi = 7$ days
$\delta$	Fernández-Villaverde and Jones (2022)	0.01 | Average fatality rate
$\lambda _g$	Direct data on vaccinations	Estimated by regions
$\alpha ^V$	Several sources	0.159 | Evidence on vaccine effectiveness
$\alpha ^C$	Several sources	0.168 | Evidence on reinfection rates
$\rho _g$	Fernández-Villaverde and Jones (2022)	Time varying infection rate calibrated as a residual using the model

From Thiessen (2020), we compute $\alpha _{g}^{j}$, that is, the shares of the different sectors in total consumption expenditure in each location. The same dataset allows deriving estimates of the share of value added on gross output, $\gamma _{g}^{j}$, and the expenditure share of each material employed in the input composite of the intermediate good produced by other industries, $\gamma _{g}^{kj}$.^13^ Table 6 provides a summary of the calibrated parameters.

The sector-specific shape parameters $\theta ^{j}$ and $\Theta ^{j}$ of the Fréchet distributions related to the productivity variables $z_{g}^{j}$ and $Z_{g}^{j}$, respectively, are obtained as follows. Consider two regions, *i* and *g*, and the bilateral trade expenditures between them, $x_{gi}^{j}$, $x_{ig}^{j}$, $X_{gi}^{j},$ and $X_{ig}^{j}$. Recall that expenditure shares $\pi _{gi}^{j}=x_{gi}^{j}/x_{g}^{j}$ and $\Pi _{gi}^{j}=X_{gi}^{j} / X_{g}^{j}$ are given in equilibrium by Eqs. (26) and (27), respectively. These expressions imply that we can write:

49 $$\begin{aligned} \frac{x_{gi}^{j}\ x_{ig}^{j}}{x_{gg}^{j}\ x_{ii}^{j}} = \left( \frac{\kappa _{gi}^{j} \kappa _{ig}^{j}}{\kappa _{gg}^{j} \kappa _{ii}^{j}}\right) ^{-\theta ^{j}} \end{aligned}$$

and

50 $$\begin{aligned} \frac{X_{gi}^{j} X_{ig}^{j}}{X_{gg}^{j}\ X_{ii}^{j}} = \left( \frac{K_{gi}^{j} K_{ig}^{j}}{K_{gg}^{j} K_{ii}^{j}}\right) ^{-\theta ^{j}}. \end{aligned}$$

Equations (49) and (50) provide gravity equations for intermediate and final products, respectively. They present bilateral trade expenditures as a function of bilateral trade costs. Equations (20) and (21) indicate that trade costs are composed of tariffs and iceberg costs. We assume, only for the purpose of estimating the trade shares, that $d_{gi}^{j} = {\mathfrak {d}}_{gi}^{j} = v_{gi} e^{\mu _{g}^{j}+\eta _{i}^{j}+\varepsilon _{gi}^{j}}$, where $v_{gi}=v_{ig}$ represents symmetric bilateral trade costs like distance (geographical, language, etc.) or belonging to a certain trade agreement; $\mu _{g}^{j}$ and $\eta _{i}^{j}$ capture sector-specific fixed effects in the importer and exporter regions, respectively, and $\varepsilon _{gi}^{j}$ is a random disturbance. Substituting those expressions for trade costs into (49) and (50), equalizing tariffs to zero and taking logs, we obtain:

51 $$\begin{aligned} \ln \left( \frac{x_{gi}^{j}\ x_{ig}^{j}}{x_{gg}^{j}\ x_{ii}^{j}}\right) = -\theta ^{j}\ln \left( \frac{v_{gi}v_{ig}}{v_{gg}v_{ii}}\ \right) + {\tilde{\varepsilon }}_{gi}^{j} \end{aligned}$$

and

52 $$\begin{aligned} \ln \left( \frac{X_{gi}^{j}\ X_{ig}^{j}}{X_{gg}^{j}\ X_{ii}^{j}}\right) = -\Theta ^{j}\ln \left( \frac{v_{gi}v_{ig}}{v_{gg}v_{ii}}\ \right) + {\tilde{\varepsilon }}_{gi}^{j}; \end{aligned}$$

where ${\tilde{\varepsilon }}_{gi}^{j}=\varepsilon _{gi}^{j}+\varepsilon _{ig}^{j}-\varepsilon _{gg}^{j}-\varepsilon _{ii}^{j}$. Hence, all asymmetric components of the iceberg costs ($\mu _{g}^{j}$, $\mu _{i}^{j}$, $\eta _{g}^{j}$ and $\eta _{i}^{j}$) have cancelled out. Additionally, we have equalized tariffs to zero because, in the estimation, we use data on export spending for the EU28 in 2013 from Thiessen (2020) but exclude the flows from and to the rest of the world. Since trade among EU members is not subject to tariffs or other trade restrictions, we can get rid of tariffs.

As a proxy for the symmetric component of the bilateral trade costs, we employ distance between regions obtained from Persyn et al. (2020). This dataset gives estimates of different distance measures between EU regions at the NUTS2 level. We choose the distance measure that provides arithmetic averages over the geodesic distance between many centroids for each region pair. Each region has more than one centroid and then $v_{gg}>1$. In the estimation, we use data on expenditure variables ($x_{gi}^{j}$ and $X_{gi}^{j}$) from the original 267 European regions considered in Thiessen (2020) to maximize the amount of information.

To obtain the trade elasticities ($-\theta ^j$ and $-\Theta ^j$), we estimate (51) and (52) for each sector *j* separately. The results of their OLS estimation are presented in Table 7, which provides the estimated coefficients along with their robust standard errors. We observe similar estimates for intermediate and final products, ranging from 1.99 to 3.09 for intermediate goods and from 1.94 to 3.09 for final products. Although small, the difference between coefficients of different sectors are often statistically significant. The smallest elasticity corresponds to construction (sector C), and the largest to public administration, defense, education, human health and social work activities (sectors O_Q). These estimates are smaller than the ones found by Caliendo and Parro (2015), who obtain values between 0.37 and 51.08, with an aggregate trade elasticity of 4.55.

**Table 7:** Sector-specific shape parameters of the Fréchet distributions

Sectors	Intermediates	Finals
A	2.778	2.775
	(0.005)	(0.005)
B_E	2.813	2.804
	(0.004)	(0.004)
C	1.993	1.943
	(0.003)	(0.003)
F	3.082	3.082
	(0.004)	(0.004)
G_I	2.718	2.742
	(0.004)	(0.004)
J	2.724	2.660
	(0.004)	(0.004)
K_L	2.944	2.944
	(0.004)	(0.004)
M_N	2.815	2.830
	(0.004)	(0.004)
O_Q	3.090	3.090
	(0.004)	(0.004)
R_U	3.026	3.023
	(0.004)	(0.004)

Robust standard errors in parentheses

We now turn to the parameters that govern the disease dynamics. We calibrate $\Lambda _{g}$ using the estimates from Dingel and Neiman (2020). In particular, we estimate the percentage of workers in each sector who can work from home $\ell _{j}$, and then we compute $\Lambda _{g}$ for each region as

$$\begin{aligned} \Lambda _{g}=1 - \sum _{j\in J}\ell _{j}\frac{x_{g}^{j}+X_{g}^{j}}{\sum _{k\in J}x_{g}^{k}+X_{g}^{k}} \end{aligned}$$

which is a weighted average where the weights are sectoral expenditure shares. This takes into account the sectoral composition of each region.

Parameter $\kappa$ is obtained from Eichenbaum et al. (2021), where it is estimated that $17\%$ of infections are related to work environments. We take $\varphi$, $\xi$ and $\delta$ from Fernández-Villaverde and Jones (2022). Specifically, the parameter $\varphi$ is set to 0.125, implying that an individual is infectious for 8 days. We assign $\xi$ a value of 0.143 so that the average case takes 15 days to fully resolve (8 days infectious plus 7 of resolving). The mortality rate $\delta$ is fixed at $1\%$.

Next, since we focus on the first wave, we equalize to zero the vaccination rate $\lambda _{g}$ and the immunization loss for vaccinated, $\alpha ^{V}$. The evidence on reinfection rates for COVID-19 is still unclear. Regarding reinfection among those not vaccinated, Sheehan et al. (2021) estimate that the protection from getting infected ranges from 81.8–84.5%. Taking into account this evidence, we fix $\alpha ^{C}=0.168$ which implies a protection from the infection of $83.15\%$.

We recover the time-variant $\rho _{g}$, representing the probability that a susceptible individual contracts the disease.^14^ Because some regions lack data on COVID-19 daily deaths (see Table 5 for details), we must divide our sample in two groups. The first group consists of areas that report daily deaths, while the second group comprises regions that only report confirmed cases. In all cases, we eliminate the geographical component by setting $\kappa =0$ to be able to calibrate $\rho _{g}$ in isolation. The main reason to do this is that our methodology recovers current $\rho _g$ based on information from future deaths. However, the start of the pandemic across regions varies substantially in the data. Then, if the geographic component were active in the calibration and, consequently, the $\{\rho _{g}\}_{g=1}^{G}$ were determined jointly, we would many times encounter a large number of zero deaths, which would render the equation system indeterminate.^15^ As will be shown later, assuming $\kappa =0$ during calibration leads to an underestimation of the number of daily deaths.

For those regions that report deaths, we extend the approach suggested by Fernández-Villaverde and Jones (2022), which essentially boils down to obtaining $\rho _{g}$ as a residual using data on deaths only. Specifically, from the death data and Eq. (48e), we can recover the resolving cases. Subsequently, the evolution of the resolving (Eq. 48c) allows obtaining the infected. Finally, the motion equation for the infected (Eq. 45) delivers $\rho _{g}$. This method is explained in detail in Appendix B.

However, sometimes in a region, we encounter three consecutive days with zero reported deaths and then the method breaks down. When this occurs, we estimate a constant infection rate ${\bar{\rho }}_{g}$, and assign it (i.e., $\rho _{g}={\bar{\rho }}_{g}$) to the region and periods in which it is not possible to recover it due to the consecutive zeros problem. The method to compute ${\bar{\rho }}_{g}$ is the following. Once we make $\kappa =0$, ${\bar{\rho }}_{g}$ can be obtained in isolation from other regions. Then, we estimate ${\bar{\rho }}_{g}$ by NLLS so as to minimize the distance of the predicted deaths from the actual death observations.

For the regions that do not report daily deaths, we assign daily values to $\rho _{g}$ based on the reported number of daily infections. Initially, using Eqs. (45) and (46), we recover a preliminary $\rho _{g}$ for each day and region from the infection data. This preliminary $\rho _{g}$ serves to generate the necessary time series of predicted fatalities $F_{g}$ from the system of Eqs. (45) to (48e). Once we have the estimated deaths, we follow the method described in Appendix B to obtain the final $\rho _{g}$ values to be used during the simulations.

**Fig. 1:**
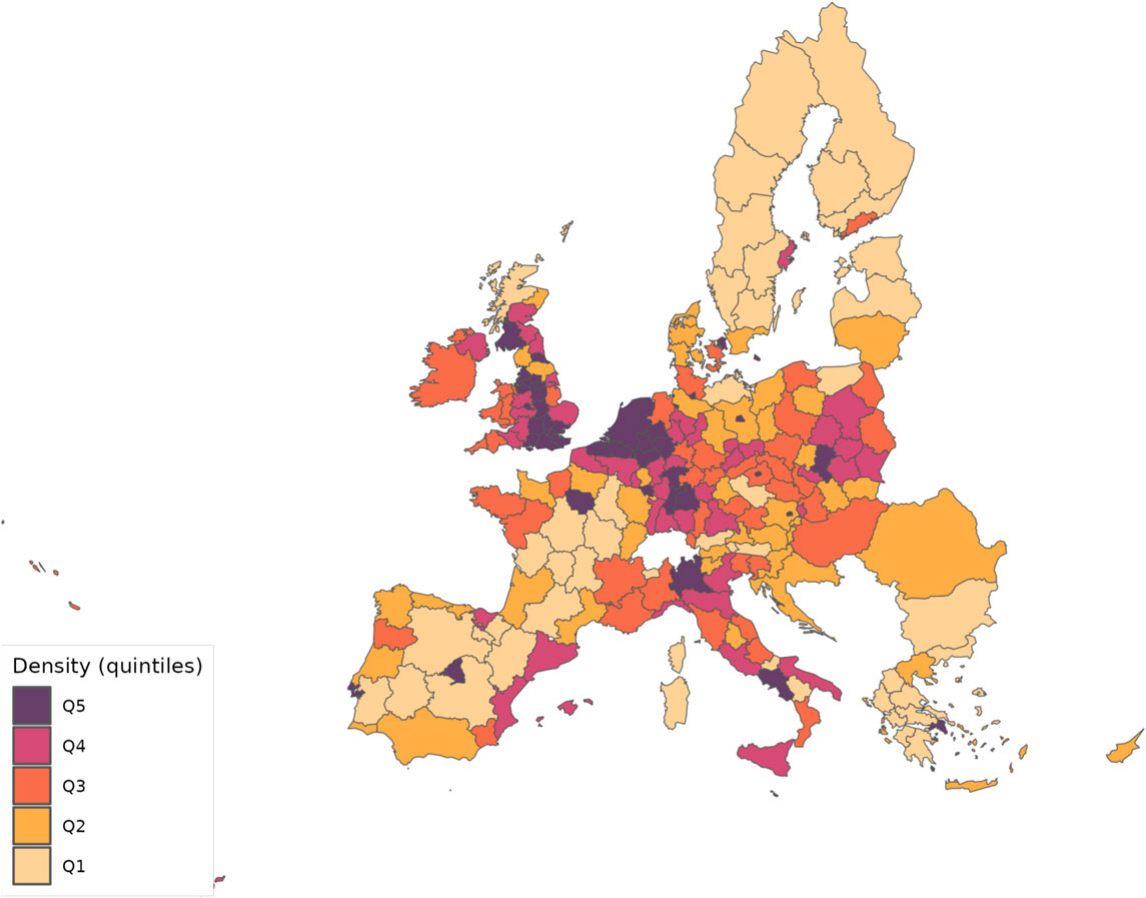
Population density across regions

**Fig. 2:**
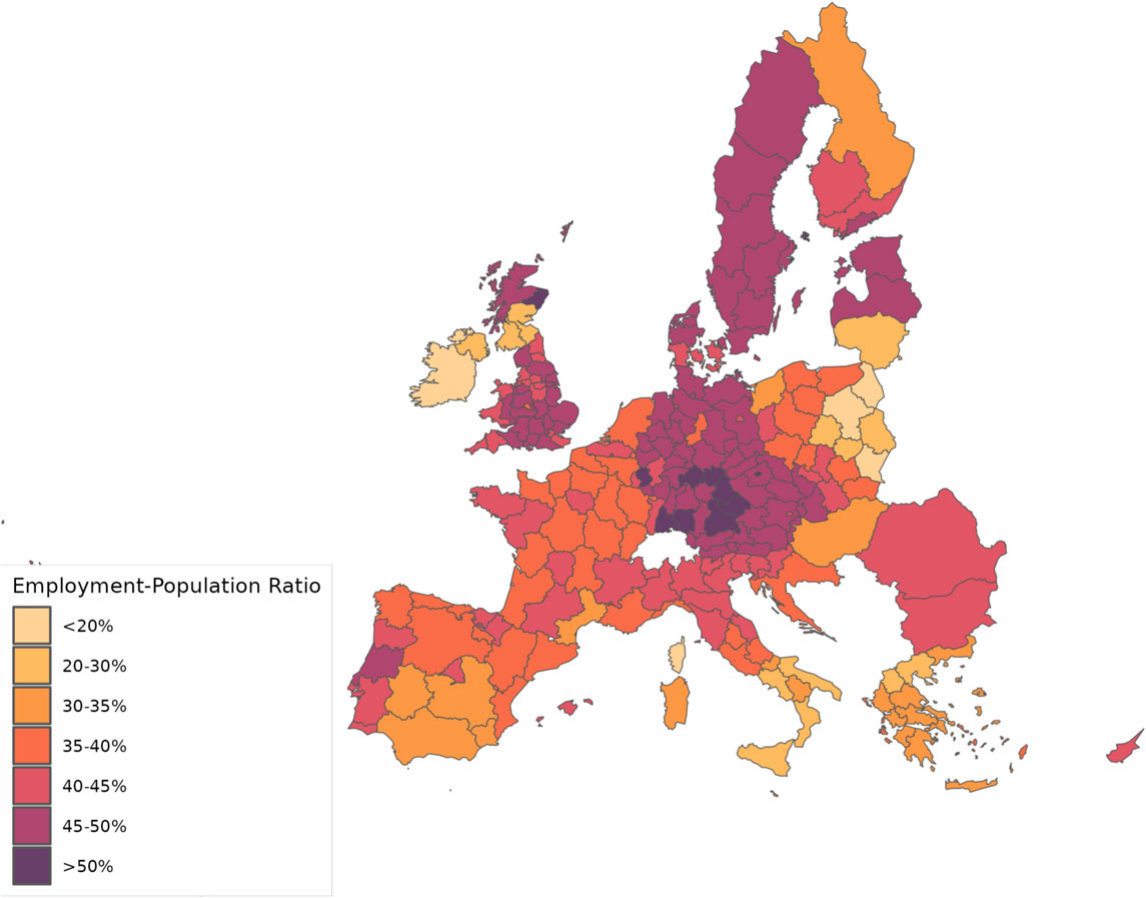
Employment to population ratio

To initiate the simulations, we need initial values. Tables 8 and 9 provides some of these initial values for different economic- and disease-related variables, respectively. The population size $N_{g}$ at the begining of the pandemic in each region comes from the same sources as deaths (see Table 5). However, for consistency with the input–output data, the rest of numbers are extracted from the year 2013. We pick the expenditure shares of intermediate goods ($\pi _{gi}^{j}$) and final products ($\Pi _{gi}^{j}$) by sector, origin and destination from Thiessen (2020). The number of workers, $L_{g}$, is obtained from different sources. In particular, for the EU28, we take employment by NUTS2 regions from regional labor statistics by Eurostat. For ROW, we extract the number of persons engaged from Penn World Tables, 10.0 (Feenstra et al. 2015). To get a better idea of the differences in population size and employment levels, Figs. 1 and 2 show population density and the employment-to-population ratio, respectively, across the regions considered.

**Table 8:** Values for certain economic variables in the initial period

Region	Employment (,000)	Wages (,000)	Tax per capita	Region	Employment (,000)	Wages (,000)	Tax per capita
AT11	134.0	26.582	$-$7.263	FR61	1351.6	36.943	$-$1.070
AT12	782.3	30.316	$-$12.218	FR62	1243.7	38.053	$-$5.194
AT13	796.1	51.764	$-$15.269	FR63	295.9	32.894	0.362
AT21	257.5	33.961	$-$11.470	FR71	2699.9	41.459	$-$6.766
AT22	584.6	34.507	$-$10.782	FR72	537.3	35.294	$-$0.426
AT31	719.2	37.197	$-$17.733	FR81	955.4	36.560	$-$12.551
AT32	273.8	39.259	$-$22.797	FR82	1955.2	40.466	$-$2.172
AT33	369.8	34.640	$-$20.136	FR83	62.2	76.115	5.869
AT34	187.4	36.073	$-$23.458	HRV	1524.0	13.286	$-$4.580
BE10	412.6	96.775	$-$15.889	HU00	3892.8	11.470	$-$4.055
BE20	2774.6	41.152	$-$15.051	IE00	1888.5	37.022	$-$12.201
BE30	1343.2	36.687	$-$2.747	ITC1	1770.7	28.079	2.619
BG00	2934.9	5.662	$-$3.473	ITC2	54.7	29.565	1.156
CYP	365.1	22.851	$-$10.685	ITC3	603.1	28.411	2.506
CZ01	649.4	27.075	$-$22.304	ITC4	4221.5	32.745	3.376
CZ02	626.2	10.431	$-$4.104	ITF1	485.9	24.778	0.550
CZ03	576.1	11.787	$-$4.412	ITF2	98.6	23.228	$-$1.146
CZ04	504.8	10.136	$-$3.759	ITF3	1580.5	25.247	1.002
CZ05	689.5	11.280	$-$2.701	ITF4	1158.4	25.028	$-$1.909
CZ06	792.9	12.658	$-$5.787	ITF5	178.6	23.431	$-$1.743
CZ07	554.2	11.232	$-$2.121	ITF6	518.2	24.357	$-$5.995
CZ08	544.1	12.379	$-$4.676	ITG1	1334.7	26.096	$-$0.919
DE11	2024.8	46.418	$-$19.429	ITG2	546.3	24.201	$-$1.089
DE12	1382.3	40.482	$-$7.598	ITH1	243.0	34.195	$-$149.067
DE13	1141.4	33.852	0.691	ITH2	229.2	31.358	$-$133.874
DE14	943.3	37.081	1.375	ITH3	2043.1	27.872	$-$21.282
DE21	2376.5	44.906	$-$30.306	ITH4	495.5	30.221	$-$64.095
DE22	626.0	31.706	3.137	ITH5	1904.1	29.684	$-$19.811
DE23	566.2	34.709	4.314	ITI1	1534.1	26.074	$-$22.678
DE24	542.5	33.663	6.094	ITI2	349.0	24.186	$-$80.430
DE25	864.6	41.955	3.737	ITI3	615.7	24.433	$-$62.624
DE26	674.6	34.375	3.437	ITI4	2225.5	33.170	$-$6.193
DE27	919.4	34.923	0.761	LTU	1292.8	10.625	$-$3.610
DE30	1604.1	36.535	$-$9.281	LUX	238.7	94.932	$-$29.984
DE40	1200.1	24.884	$-$19.873	LVA	893.9	10.539	$-$9.128
DE50	299.1	51.296	2.623	MLT	181.6	18.957	$-$21.888
DE60	885.6	54.994	$-$18.539	NL00	8285.3	39.156	$-$15.015
DE71	1912.2	45.963	$-$37.108	PL11	1247.7	6.867	$-$1.807
DE72	503.5	33.955	5.715	PL12	1044.0	6.136	$-$10.795
DE73	591.4	37.559	4.617	PL21	1314.9	8.955	$-$3.773
DE80	741.9	26.457	1.978	PL22	1903.3	10.471	$-$6.689
DE91	734.0	40.740	2.966	PL31	957.8	5.988	$-$0.758
DE92	1013.5	35.950	$-$0.458	PL32	800.1	7.504	$-$0.548
DE93	804.8	24.695	2.341	PL33	554.0	6.192	$-$1.144
DE94	1214.6	30.523	$-$5.334	PL34	453.3	7.127	$-$0.924
DEA1	2364.0	40.903	$-$31.247	PL41	1365.6	9.896	$-$6.177
DEA2	2013.5	40.181	$-$29.272	PL42	572.4	9.223	$-$3.354
DEA3	1209.3	32.514	$-$4.406	PL43	404.7	7.845	$-$2.437
DEA4	968.6	35.664	0.693	PL51	1055.6	11.646	$-$6.788
DEA5	1623.0	36.641	$-$3.571	PL52	346.1	9.092	$-$3.167
DEB1	717.0	30.416	2.206	PL61	761.4	8.427	$-$2.292
DEB2	264.5	27.323	5.418	PL62	528.7	7.500	$-$1.866
DEB3	981.0	34.487	$-$1.896	PL63	894.1	9.448	$-$4.990
DEC0	464.8	37.471	$-$16.756	PT11	1543.9	14.458	$-$5.570
DED2	743.8	29.692	$-$2.809	PT15	186.9	14.699	$-$9.925
DED4	688.1	26.945	$-$1.295	PT16	1059.2	12.866	$-$5.925
DED5	474.6	30.726	$-$11.489	PT17	1132.9	26.130	$-$10.925
DEE0	1048.9	26.492	$-$0.673	PT18	298.5	14.760	$-$5.861
DEF0	1336.9	29.431	$-$1.028	PT20	99.2	16.322	$-$6.223
DEG0	1067.1	26.589	0.795	PT30	108.8	16.377	$-$17.914
DK01	858.4	61.770	$-$17.926	RO00	8549.1	5.349	$-$4.747
DK02	367.7	37.375	$-$10.079	ROW	942281.9	6.379	$-$21.974
DK03	538.9	47.297	$-$21.957	SE11	1133.4	57.620	$-$32.283
DK04	606.0	46.647	$-$19.716	SE12	750.4	43.493	$-$10.368
DK05	265.2	45.533	$-$22.082	SE21	394.9	42.934	$-$16.470
EE00	621.3	13.873	$-$8.711	SE22	672.4	43.034	$-$14.361
EL11	187.4	13.975	$-$1.287	SE23	951.4	45.211	$-$19.223
EL12	553.6	15.569	$-$2.522	SE31	387.3	40.281	$-$14.935
EL13	77.1	22.555	$-$10.095	SE32	172.5	41.157	$-$18.188
EL14	235.5	13.886	$-$2.724	SE33	242.2	44.075	$-$19.639
EL21	103.9	13.513	$-$1.575	SI01	473.5	16.270	$-$6.015
EL22	75.2	13.777	$-$6.052	SI02	432.4	23.982	$-$8.198
EL23	202.7	13.852	$-$3.918	SK01	315.2	25.285	$-$37.207
EL24	171.3	15.648	$-$7.450	SK02	824.8	9.903	$-$4.622
EL25	191.3	13.472	$-$3.304	SK03	563.9	10.025	$-$3.793
EL30	1312.0	22.400	$-$11.083	SK04	625.4	8.968	$-$2.845
EL41	65.6	14.652	$-$1.155	UKC1	491.7	17.574	$-$3.107
EL42	122.8	17.225	$-$7.810	UKC2	641.4	18.338	$-$4.316
EL43	214.8	14.179	$-$5.425	UKD1	240.1	17.303	$-$5.735
ES11	1006.4	23.089	$-$9.808	UKD3	1215.3	20.438	$-$5.678
ES12	369.4	25.605	$-$8.770	UKD4	639.2	18.059	$-$5.299
ES13	222.5	23.698	$-$7.971	UKD6	431.8	20.000	$-$482.572
ES21	873.6	33.219	$-$10.681	UKD7	657.2	18.000	$-$192.049
ES22	258.1	30.630	$-$11.029	UKE1	422.9	17.903	$-$5.425
ES23	124.5	24.648	$-$13.305	UKE2	386.9	17.838	$-$6.861
ES24	515.3	27.559	$-$11.060	UKE3	621.8	16.243	$-$4.002
ES30	2718.1	35.590	$-$12.687	UKE4	1006.7	20.347	$-$5.931
ES41	916.4	24.179	$-$7.936	UKF1	973.3	18.111	$-$4.702
ES42	712.3	21.324	$-$8.212	UKF2	816.7	20.108	$-$5.229
ES43	339.7	21.775	$-$4.217	UKF3	342.8	13.848	$-$6.070
ES51	2969.6	29.935	$-$11.567	UKG1	642.1	18.541	$-$7.249
ES52	1771.2	23.223	$-$8.090	UKG2	754.6	15.956	$-$5.027
ES53	475.8	23.426	$-$10.654	UKG3	1136.4	20.761	$-$5.159
ES61	2571.5	23.665	$-$6.053	UKH1	1155.3	19.056	$-$6.970
ES62	514.9	23.302	$-$12.779	UKH2	885.8	23.202	$-$5.794
ES63	25.6	33.891	$-$0.382	UKH3	839.2	16.779	$-$4.313
ES64	24.6	31.541	0.014	UKI1	1524.1	63.399	$-$51.863
ES70	729.7	24.064	$-$7.055	UKI2	2238.5	19.722	$-$4.353
FI19	600.6	38.136	$-$73.287	UKJ1	1184.5	30.080	$-$10.911
FI1B	796.1	48.499	1.898	UKJ2	1360.9	20.605	$-$5.798
FI1C	502.2	37.192	3.059	UKJ3	938.1	21.087	$-$5.775
FI1D	542.9	36.367	1.162	UKJ4	806.6	16.680	$-$4.745
FI20	15.0	47.661	6.337	UKK1	1171.8	20.758	$-$7.030
FR10	5277.6	64.750	$-$17.352	UKK2	615.3	15.670	$-$4.123
FR21	506.9	37.200	$-$5.522	UKK3	238.8	13.092	$-$3.574
FR22	728.0	34.172	$-$1.195	UKK4	512.6	16.107	$-$4.338
FR23	717.5	39.026	$-$0.711	UKL1	851.5	13.342	$-$3.091
FR24	1000.7	36.918	$-$5.498	UKL2	535.7	18.690	$-$6.418
FR25	578.5	35.348	0.511	UKM2	962.8	18.727	$-$3.527
FR26	639.7	36.367	$-$0.802	UKM3	991.2	19.921	$-$2.492
FR30	1492.6	40.037	$-$9.713	UKM5	251.7	39.198	$-$22.072
FR41	904.6	33.876	$-$3.644	UKM6	233.6	14.205	$-$7.639
FR42	809.4	38.182	$-$1.524	UKN0	797.2	15.945	$-$3.035
FR43	468.6	34.315	$-$1.568				
FR51	1509.7	38.074	$-$5.185				
FR52	1336.1	35.452	$-$6.330				
FR53	714.1	33.652	$-$2.779

**Table 9:** Values for certain disease-related variables in the initial period

Region	Start of the pandemic	Non-telematic workers (%)	Infected	Region	Start of the pandemic	Non-telematic workers (%)	Infected
AT11	27-Mar	0.643	103.044	FR61	22-Mar	0.652	4015.271
AT12	19-Mar	0.636	1949.035	FR62	19-Mar	0.637	1854.444
AT13	14-Mar	0.593	1852.674	FR63	22-Mar	0.658	927.643
AT21	30-Mar	0.634	411.027	FR71	18-Mar	0.641	20220.949
AT22	17-Mar	0.645	2239.692	FR72	26-Mar	0.649	1584.200
AT31	23-Mar	0.646	1532.013	FR81	18-Mar	0.681	4746.184
AT32	26-Mar	0.619	1097.628	FR82	19-Mar	0.639	8072.069
AT33	21-Mar	0.619	2603.076	FR83	19-Mar	0.651	1388.133
AT34	29-Mar	0.640	616.282	HRV	27-Mar	0.628	1139.221
BE10	10-Mar	0.606	4803.839	HU00	20-Mar	0.659	1439.566
BE20	14-Mar	0.650	25756.309	IE00	21-Mar	0.611	7687.612
BE30	15-Mar	0.658	23732.681	ITC1	05-Mar	0.653	10748.732
BG00	27-Mar	0.646	1336.648	ITC2	17-Mar	0.657	3390.699
CYP	29-Mar	0.606	327.516	ITC3	04-Mar	0.652	5371.631
CZ01	24-Mar	0.590	2612.614	ITC4	20-Feb	0.650	65345.453
CZ02	30-Mar	0.666	832.420	ITF1	14-Mar	0.658	4934.933
CZ03	06-Apr	0.668	630.218	ITF2	19-Mar	0.661	464.173
CZ04	30-Mar	0.660	912.995	ITF3	13-Mar	0.659	5534.914
CZ05	01-Apr	0.672	425.525	ITF4	07-Mar	0.667	1735.225
CZ06	01-Apr	0.662	734.146	ITF5	26-Mar	0.668	858.063
CZ07	30-Mar	0.666	438.712	ITF6	18-Mar	0.679	2213.232
CZ08	30-Mar	0.662	603.674	ITG1	16-Mar	0.665	4125.970
DE11	06-Mar	0.667	3280.369	ITG2	20-Mar	0.665	2285.404
DE12	12-Mar	0.658	2525.148	ITH1	13-Mar	0.635	3647.874
DE13	08-Mar	0.667	3829.431	ITH2	14-Mar	0.635	6967.356
DE14	15-Mar	0.668	5409.605	ITH3	02-Mar	0.640	4053.239
DE21	13-Mar	0.645	10327.778	ITH4	09-Mar	0.636	3351.826
DE22	16-Mar	0.665	5266.321	ITH5	28-Feb	0.641	12422.694
DE23	15-Mar	0.662	6770.607	ITI1	11-Mar	0.639	8172.510
DE24	15-Mar	0.662	3235.555	ITI2	19-Mar	0.637	2022.638
DE25	19-Mar	0.653	6599.939	ITI3	03-Mar	0.636	5089.702
DE26	11-Mar	0.660	2449.937	ITI4	07-Mar	0.639	3240.823
DE27	13-Mar	0.663	2273.360	LTU	25-Mar	0.681	568.314
DE30	17-Mar	0.620	6112.987	LUX	18-Mar	0.598	1508.562
DE40	24-Mar	0.639	4756.678	LVA	11-Apr	0.649	499.430
DE50	25-Mar	0.642	1018.454	MLT	10-Apr	0.599	98.637
DE60	12-Mar	0.626	2122.158	NL00	09-Mar	0.636	15453.226
DE71	19-Mar	0.626	5456.108	PL11	02-Apr	0.663	466.260
DE72	23-Mar	0.662	1030.016	PL12	25-Mar	0.611	2133.254
DE73	22-Mar	0.661	3268.592	PL21	01-Apr	0.652	730.002
DE80	26-Mar	0.653	847.376	PL22	28-Mar	0.657	2415.361
DE91	17-Mar	0.659	3850.167	PL31	29-Mar	0.673	369.333
DE92	19-Mar	0.656	3142.748	PL32	01-Apr	0.671	629.576
DE93	25-Mar	0.660	1810.941	PL33	15-Apr	0.669	287.577
DE94	14-Mar	0.665	2100.665	PL34	15-Apr	0.675	74.174
DEA1	11-Mar	0.637	3617.624	PL41	31-Mar	0.669	2145.465
DEA2	06-Mar	0.610	4521.161	PL42	18-Apr	0.666	546.586
DEA3	17-Mar	0.660	4106.123	PL43	22-Jul	0.672	497.269
DEA4	17-Mar	0.664	1619.907	PL51	24-Mar	0.659	648.968
DEA5	18-Mar	0.668	4113.175	PL52	06-Apr	0.672	781.619
DEB1	16-Mar	0.659	1420.570	PL61	07-Apr	0.669	979.346
DEB2	25-Mar	0.657	586.702	PL62	10-Aug	0.677	232.002
DEB3	20-Mar	0.665	2279.947	PL63	21-Apr	0.656	1012.212
DEC0	17-Mar	0.635	2913.969	PT11	20-Mar	0.646	7663.833
DED2	19-Mar	0.634	1516.737	PT15	01-Apr	0.641	402.946
DED4	21-Mar	0.641	3703.584	PT16	20-Mar	0.656	3630.319
DED5	26-Mar	0.623	669.239	PT17	20-Mar	0.603	2762.330
DEE0	20-Mar	0.662	1689.044	PT18	24-Jun	0.666	788.249
DEF0	16-Mar	0.656	2470.665	PT20	10-Apr	0.667	377.163
DEG0	21-Mar	0.661	2215.732	PT30	13-Oct	0.652	0.001
DK01	28-Feb	0.599	5.233	RO00	21-Mar	0.651	8706.891
DK02	03-Mar	0.649	4.319	ROW	25-Feb	0.635	339.840
DK03	01-Mar	0.651	3.753	SE11	26-Mar	0.580	25240.461
DK04	15-Jul	0.654	0.867	SE12	26-Mar	0.623	7369.381
DK05	08-Mar	0.659	3.542	SE21	26-Mar	0.635	1349.541
EE00	30-Mar	0.652	1611.450	SE22	26-Mar	0.615	1041.884
EL11	12-Oct	0.632	3.748	SE23	26-Mar	0.616	2741.393
EL12	15-Jul	0.630	3.283	SE31	26-Mar	0.629	2819.716
EL13	10-Aug	0.635	3.339	SE32	07-Apr	0.614	1370.495
EL14	29-Jul	0.637	3.819	SE33	05-Apr	0.623	1578.364
EL21	28-Aug	0.630	3.100	SI01	23-Mar	0.670	1414.387
EL22	09-Nov	0.588	3.039	SI02	28-Mar	0.643	983.944
EL23	15-Aug	0.630	3.074	SK01	18-Aug	0.614	22.267
EL24	08-Sep	0.643	3.163	SK02	15-Mar	0.665	3.454
EL25	09-Aug	0.630	3.274	SK03	25-Aug	0.667	9.379
EL30	09-Nov	0.607	10.546	SK04	10-Aug	0.669	9.062
EL41	14-Aug	0.598	3.531	UKC1	15-Mar	0.627	4.767
EL42	11-Oct	0.554	3.442	UKC2	13-Mar	0.624	5.420
EL43	09-Aug	0.580	3.797	UKD1	09-Mar	0.631	4.039
ES11	15-Mar	0.640	4876.296	UKD3	09-Mar	0.620	5.866
ES12	18-Mar	0.632	4225.524	UKD4	13-Mar	0.631	5.712
ES13	20-Mar	0.635	3846.428	UKD6	15-Mar	0.592	5.117
ES21	07-Mar	0.635	5876.978	UKD7	12-Mar	0.598	5.689
ES22	16-Mar	0.644	6582.146	UKE1	18-Mar	0.635	4.117
ES23	11-Mar	0.648	2601.451	UKE2	15-Mar	0.626	4.362
ES24	07-Mar	0.639	1332.486	UKE3	08-Mar	0.627	5.025
ES30	07-Mar	0.587	73177.142	UKE4	12-Mar	0.622	5.105
ES41	13-Mar	0.642	21223.621	UKF1	06-Mar	0.631	4.893
ES42	11-Mar	0.637	20920.870	UKF2	10-Mar	0.627	5.393
ES43	15-Mar	0.645	7275.205	UKF3	16-Mar	0.639	4.339
ES51	09-Mar	0.625	22908.766	UKG1	09-Mar	0.631	4.532
ES52	12-Mar	0.633	13245.641	UKG2	10-Mar	0.631	4.823
ES53	19-Mar	0.583	3553.364	UKG3	06-Mar	0.623	6.405
ES61	13-Mar	0.630	11181.436	UKH1	10-Mar	0.628	4.955
ES62	22-Mar	0.649	3989.206	UKH2	08-Mar	0.622	4.615
ES63	04-Apr	0.637	107.564	UKH3	10-Mar	0.627	5.537
ES64	03-Sep	0.639	73.033	UKI1	01-Mar	0.593	6.144
ES70	16-Mar	0.590	3136.775	UKI2	04-Mar	0.612	12.268
FI19	05-Mar	0.620	5986.344	UKJ1	05-Mar	0.612	5.290
FI1B	26-Feb	0.653	5548.468	UKJ2	07-Mar	0.617	5.405
FI1C	06-Mar	0.666	3868.857	UKJ3	06-Mar	0.617	4.509
FI1D	03-Mar	0.669	3197.378	UKJ4	11-Mar	0.627	5.676
FI20	21-Mar	0.661	334.259	UKK1	09-Mar	0.621	4.940
FR10	18-Mar	0.630	84443.478	UKK2	17-Mar	0.630	3.947
FR21	19-Mar	0.660	5846.306	UKK3	14-Mar	0.630	3.853
FR22	18-Mar	0.659	12914.460	UKK4	11-Mar	0.628	3.426
FR23	19-Mar	0.656	3153.497	UKL1	10-Mar	0.630	6.153
FR24	22-Mar	0.651	7177.193	UKL2	09-Mar	0.628	6.211
FR25	23-Mar	0.653	2811.046	UKM2	09-Mar	0.624	4.763
FR26	18-Mar	0.656	6448.034	UKM3	08-Mar	0.625	5.131
FR30	18-Mar	0.648	7110.021	UKM5	19-Jul	0.614	1.513
FR41	18-Mar	0.656	24535.396	UKM6	13-Mar	0.634	3.505
FR42	18-Mar	0.650	34338.163	UKN0	15-Mar	0.630	4.439
FR43	18-Mar	0.658	7869.257				
FR51	21-Mar	0.650	6747.541				
FR52	18-Mar	0.645	3074.450				
FR53	21-Mar	0.657	2696.476				

**Fig. 3:**
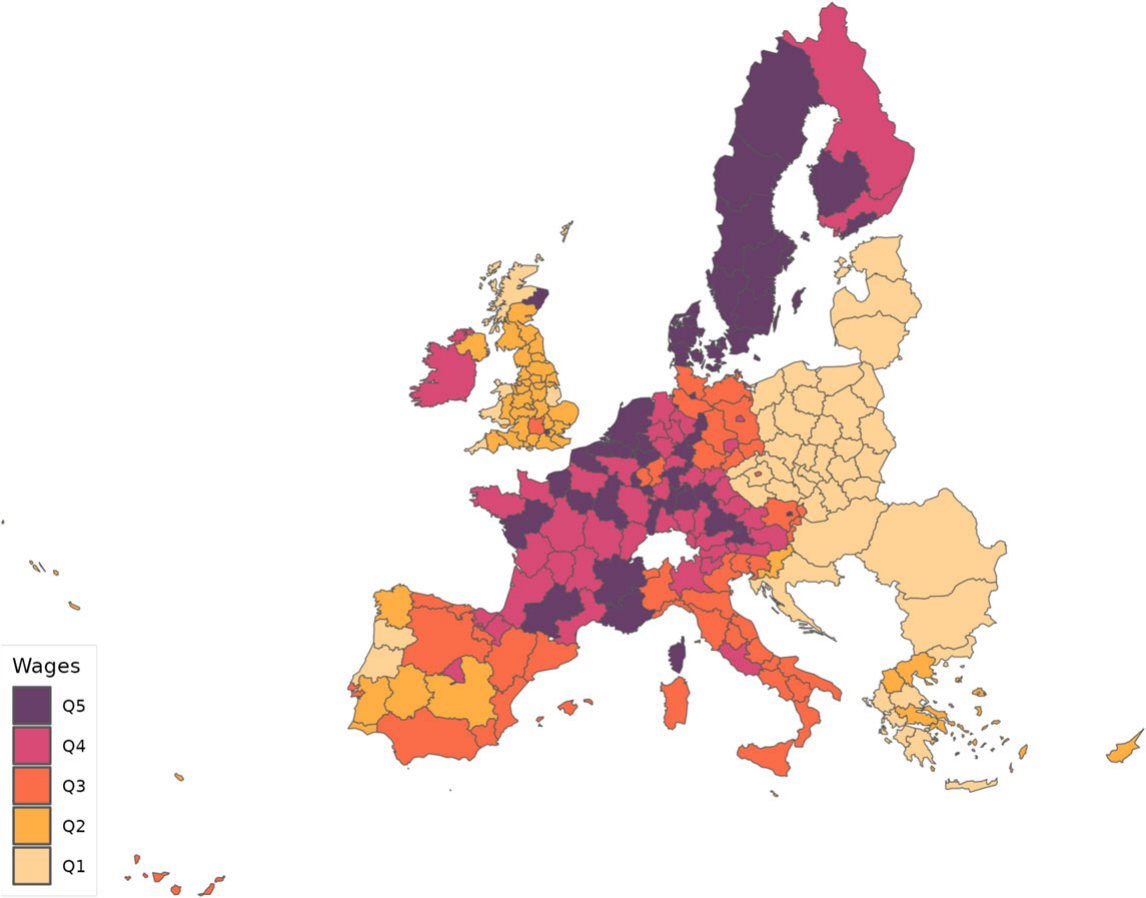
Wages

**Fig. 4:**
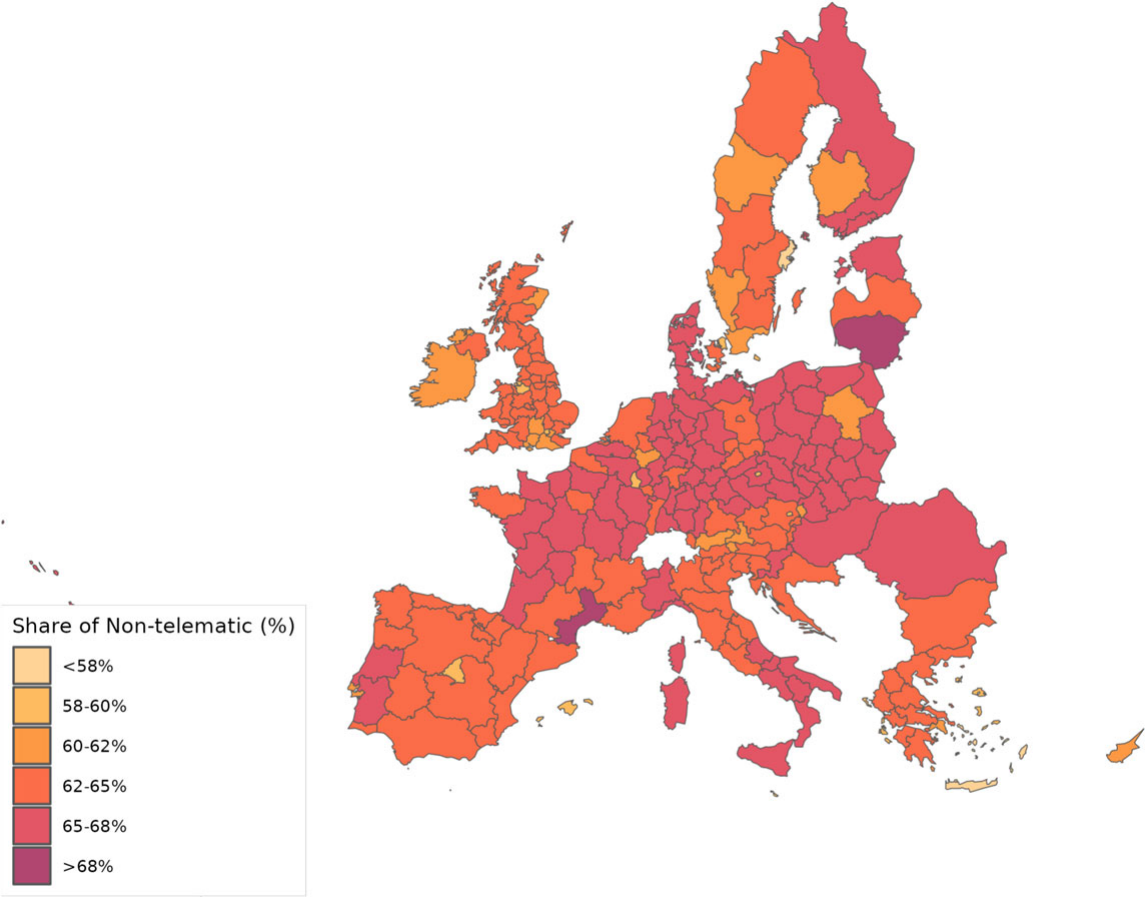
Share of non-telematic workers

Wages, $w_{g}$, are calculated as total compensation of employees divided by the employment figures. Total compensation of employees for the EU27 group (EU28 minus the United Kingdom) comes from the Eurostat regional accounts data, whereas for the UK, we get them from the gross annual pay for all employee jobs reported by Annual Survey of Hours and Earnings. For ROW, compensation of employees are directly taken from Thiessen (2020). Lump-sum taxes $\tau _{g}$ are calibrated so as to reproduce the observed total expenditures on final products by region and sector ($X_{g}^{j}$) provided by Thiessen (2020). Figures 3 and 4 show the distribution of wages and the share of non-telematic workers, respectively, across the regions considered.

Subsidies for intermediate goods ($s_{g}^{j}$) and final good products/materials (${\mathfrak {s}}_{g}^{j}$) are equalized to zero. Bilateral ad valorem tariffs for intermediate and final goods, denoted by $\tau _{gi}^{j}$ and $T_{gi}^{j}$, respectively, are zero among EU members. The only tariffs different from zero are those related to ROW. We assign values to the different industries using information from Eurostat (2017) on average import tariffs imposed by the EU28 to other countries in 2013 and WITS - UNCTAD TRAINS information.

Finally, since the mechanism we emphasize involves trade through the transportation of goods and tourism, it is essential to account for the anti-COVID-19 policies that restricted the movement of people (i.e., mobility restrictions). As we show in Sect. 6.1, the parameter $\rho _{g}$ already reflects the impact of anti-COVID policies. However, we calibrate $\rho _{g}$ excluding the geographic component. As a result, $\rho _{g}$ primarily captures the effect of anti-COVID policies on the social component. To more comprehensively account for the influence of mobility restrictions via the geographic component, we consider their impact on the amount of labor available for production, sectoral consumption expenditure shares, and trade costs.

**Fig. 5:**
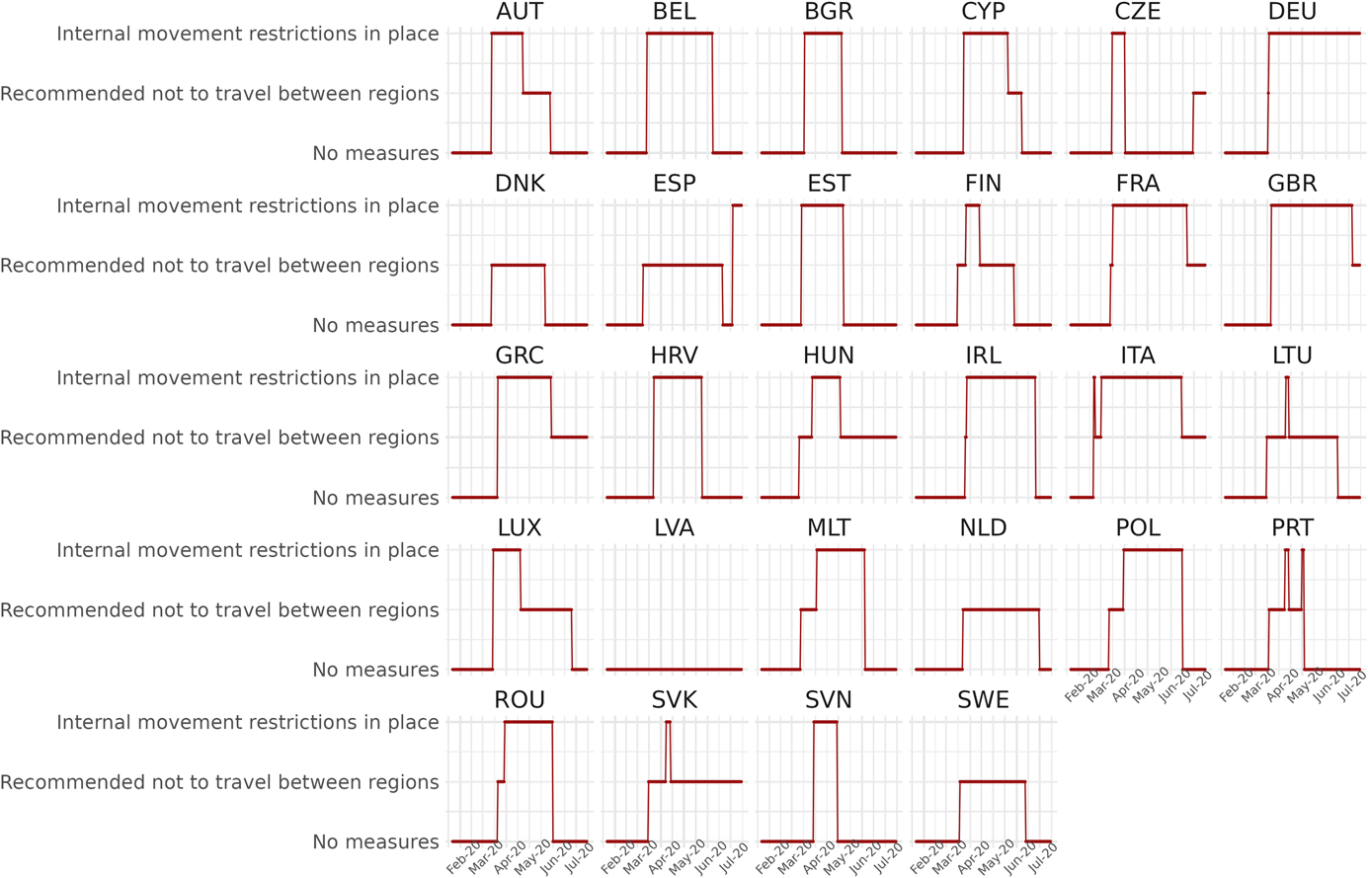
Internal movement restrictions

**Fig. 6:**
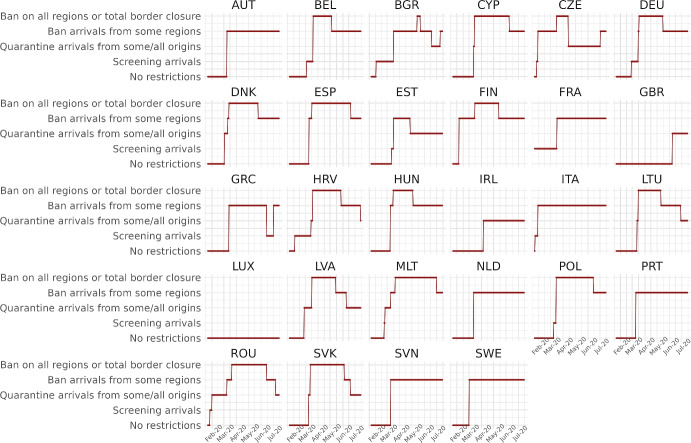
International movement restrictions

Figures 5 and 6 plot the internal and international restrictions to the movement of people, respectively, imposed by the countries in our sample during the first wave of the COVID-19 pandemic. These data are sourced from Hale et al. (2021)—the Oxford COVID-19 Government Response Tracker (OxCGRT). Hale et al. (2021) classifies the restrictions on internal movement within countries in three categories: (a1) no measures; (a2) recommended not to travel between regions; and (a3) internal movement restrictions in place. In our analysis, we consider internal movement of people to be restricted in a certain location on a particular day if its national government implemented either (a2) or (a3).

Importantly, internal mobility restrictions were closely linked to productive lockdowns because the affected economic activities were those that heavily relied on physical presence and the transportation of goods and people. Consequently, we will use these two terms interchangeably. Moreover, we will assume that when internal mobility restrictions are in place, the sectors impacted by productive lockdown are shut down and cannot sell their products.

In turn, Hale et al. (2021) classifies restrictions on international movement into five categories: (b1) no restrictions; (b2) screening arrivals; (b3) quarantine arrivals from some/all origins; (b4) ban arrivals from some regions; and (b5) ban on all regions or total border closure. We consider international movement restrictions to be in effect for a country on a particular day when either (b3), (b4) or (b5) is implemented. Under these conditions, consumers and firms from foreign countries are unable to purchase domestic products from sectors disrupted by productive lockdowns.

Figures 5 and 6 indicate that the strength of the restrictions and their duration significantly vary across nations. For example, regarding internal movement, Denmark (DNK), Latvia (LVA), the Netherlands (NLD), and Sweden (SWE) never imposed strong restrictions, while Italy (ITA) imposed severe restrictions most of the time. Likewise, Greece (GRC), the Netherlands, Portugal (PRT), Slovenia (SVN), and Sweden imposed a total border closure during long periods of time, whereas Great Britain (GBR) never imposed any ban on international arrivals.

In several countries, industries that were fully closed for significant portions of the pandemic include hotels, restaurants, and accommodation; estate and travel agencies; and leisure and recreation services. Therefore, given the level of aggregation in our dataset, we consider that, within our industry classification, the sectors most affected by the mobility restrictions correspond to category G_I—wholesale and retail trade, transport, accommodation and food service activities—and category R_U—arts, entertainment and recreation; other service activities; activities of household; and extra-territorial organizations and bodies. These two categories represent sectors 5 and 10, respectively, in our simulations.

While sectors 5 and 10 encompass the industries most impacted by the productive lockdown, they contain as well other sectors that were less severely affected.^16^ Nevertheless, the fraction of workers employed by categories G_I and R_U combined ranges from 0.23 to 0.37 in our sample of regions, aligning closely with the lower and upper bounds found by Fana et al. (2020) for the share of workers most affected by lockdown policies—in particular, those employed in mostly non-essential and closed activities—in a set of EU and UK economies. This provides confidence that our approach reasonably captures the proportion of the economy affected by anti-COVID policies.

Consequently, we assume that when the internal movement of people is restricted, labor availability, consumption expenditure shares, and trade costs are impacted. Specifically, we set the consumption expenditure shares of sectors 5 and 10 to *zero* (i.e., $\alpha ^{5}_{g}=\alpha ^{10}_{g}=0$), reflecting consumers’ inability to purchase goods from these sectors. Simultaneously, we proportionally increase the consumption expenditure shares of other sectors so that $\sum _{j=1}^{10} \alpha ^{j}_{g}=1$ continues to hold. In turn, the total labor force available in the region on that day ($L_{g}$) is reduced by the amount allocated in our model to sectors 5 and 10 based on intermediate goods expenditures derived from the Rhomolo-MRIO Tables for 2013, as these workers are sent home due to the restrictions.

Additionally, the rich trade structure of our model allows us to account for the effect of mobility restrictions by adjusting the bilateral trade cost parameters. When internal movement restrictions are imposed in region *g*, sales from the affected sectors in region *g* to all regions are not possible. To model this, we multiply parameters $d_{ig}^{j}$ and ${\mathfrak {d}}_{ig}^{j}$, for $j=5,10$ and $i=1,...,G$, by a large factor of $10^{10}$ relative to their benchmark values to sufficiently reduce trade from these two sectors in region *g* to every destination *i*.

Similarly, to incorporate international movement restrictions into the geographic component, we assume that when such restrictions are implemented in location *g* on a particular day, the iceberg cost parameters $d_{gi}^{j}$ and ${\mathfrak {d}}_{gi}^{j}$, for $j=5,10$ and $i=1,...,G$, are multiplied by a factor of $10^{10}$ relative to their benchmark values. This adjustement sufficiently reduces trade from every origin *i* to sectors 5 and 10 in region *g*.

## Results

We focus on the first wave of the COVID-19 pandemic, and more specifically, in the period that goes from February 25 to July 15, 2020. We start by performing an external validation exercise for the calibrated values of the parameter $\rho _g$. Subsequently, we compare the fatalities caused by the pandemic in the UK and the European Union, and assess how well the model reproduces them. Finally, we present results from the policy counterfactual simulations.

### External validity

Parameter $\rho _{g}$ is one of the crucial elements for our results. We argue that its variations proxy the evolution of the anti-COVID policy. However, there are aspects that the model does not consider and $\rho _{g}$ might also be capturing. To understand better what $\rho _g$ is capturing, we first correlate the initial value of $\rho _g$ with a potential determinant such as differences in population density. The first column in Table 10 shows the estimates of regressing the initial $\rho _g$ with the log of the population density of the regions after controlling for latitude and longitude. We find that the coefficient is positive and statistically significant, which suggests that denser regions tend to have a larger initial $\rho _g$. If we control for initial deaths (column (2) in Table 10) and the share of non-telematic workers (column (3)), the coefficient for the log of the density still remains positive, statistically significant, and very similar in magnitude.

**Table 10:** Density and initial $\rho _{g}$

	(1)	(2)	(3)
Log of density	0.018***	0.018***	0.016***
	(0.004)	(0.005)	(0.005)
Initial deaths		0.000	0.000
		(0.000)	(0.000)
Share of non-telematic workers			$-$0.004
			(0.003)
Num.Obs	210	210	210
$R^{2}$	0.210	0.212	0.219

*$$p< 0.1$$, **$$ p < 0.05$$, ***$$ p < 0.01$$. Robust standard errors in parentheses. All regressions control for latitude and longitude. The dependent variable is the initial $$\rho _{g}$$ across regions

Secondly, we need to show whether our calibrated $\rho _g$ in fact reflects features of anti-COVID policy differences across European regions. To do so, we regress the values of $\rho _g$ for each region and time period on three different indices that reflect anti-COVID-19 responses. These indices are obtained from the Oxford COVID-19 Government Response Tracker Database (Hale et al. 2021). Since policy decisions were primarily made at the country level, the Oxford COVID-19 Government Response Tracker Database only provides indicators at the national level. Thus, we regress the calibrated $\rho _g$ for each region over time on each index, adding region, country and date fixed effects. These fixed effects absorb unobserved constant characteristics at the region and country level, and control for common time trends.

The three indices we consider are the containment and health index, the average stringency index, and the government response index. The three of them capture policies that tried to prevent the spread of COVID-19, but each index captures different aspects. The containment and health index measures policies related to containment and closure policies and health system policies. The average stringency index contains all measures related to containment and closure policies as well as public information campaigns. Finally, the government response index is a composite of all aggregates (containment and closure policies, economic policies, health system policies, and vaccination policies).

Table 11 provides the results. The estimated coefficients are negative and statistically significant for the containment and health index, as well as for the government response index. The average stringency index again shows the expected negative coefficient, but it is not statistically significant. This could be because during the first wave of the pandemic, most of the policies implemented were related to containment and closure, which receive less weight in an average index that includes multiple other policies.

**Table 11:** Stringency index and $\rho _{g,t}$

	(1)	(2)	(3)
Containment and Health Index	$-$0.035***		
	(0.011)		
Average Stringency Index		$-$0.006	
		(0.009)	
Government Response Index			$-$0.031**
			(0.012)
Num.Obs	32518	32518	32518
$R^{2}$	0.433	0.432	0.433
FE: Region	X	X	X
FE: Country	X	X	X
FE: Date	X	X	X

*$p<0.1$, **$p< 0.05$, ***$p < 0.01$. Newey–West standard errors in parentheses. All regressions include region, country, and date fixed effects. The dependent variable is the calibrated $\rho _{g}$ for each region over time. Average Stringency Index, Containment and Health Index, and Government Response Index are obtained from The Oxford Covid-19 Government Response Tracker (OxCGRT). The regressions use daily variation across countries

Taken together, these results suggest that $\rho _{g}$ indeed reflects anti-COVID policy responses across European regions. In the following subsection, we will assess how well the calibrated $\rho _g$ reproduces the fatality data.

### The COVID-19 fatalities

**Fig. 7:**
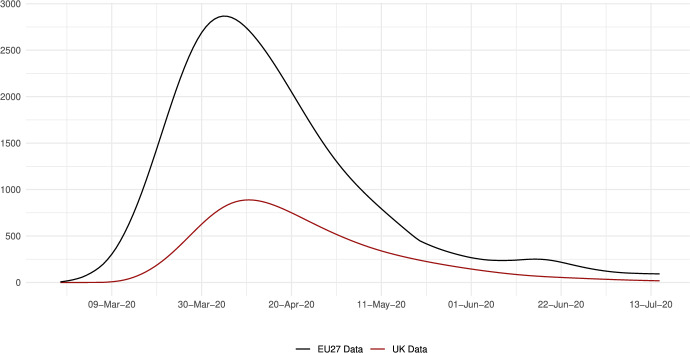
Total daily deaths in the EU27 and the UK

Figure 7 provides the total daily number of deaths in the European Union (EU27) and in the UK. This number, in our smoothed time series, reached a maximum value of 2,867 in the EU27 on April 4, and 887 in the UK on April 11. That is, the pandemic in the UK evolved with a one-week lag compared to the European Union.

**Fig. 8:**
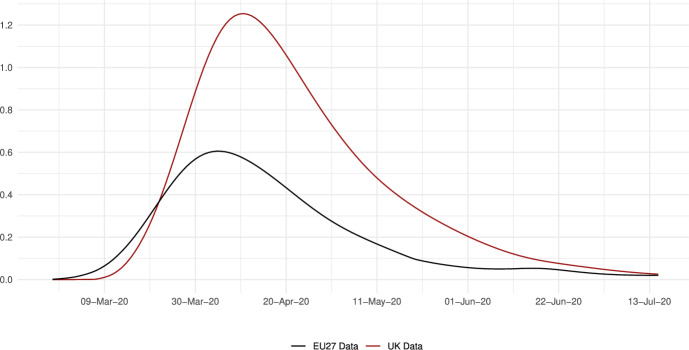
Daily deaths per 100,000 inhabitants in the EU27 and the UK

**Fig. 9:**
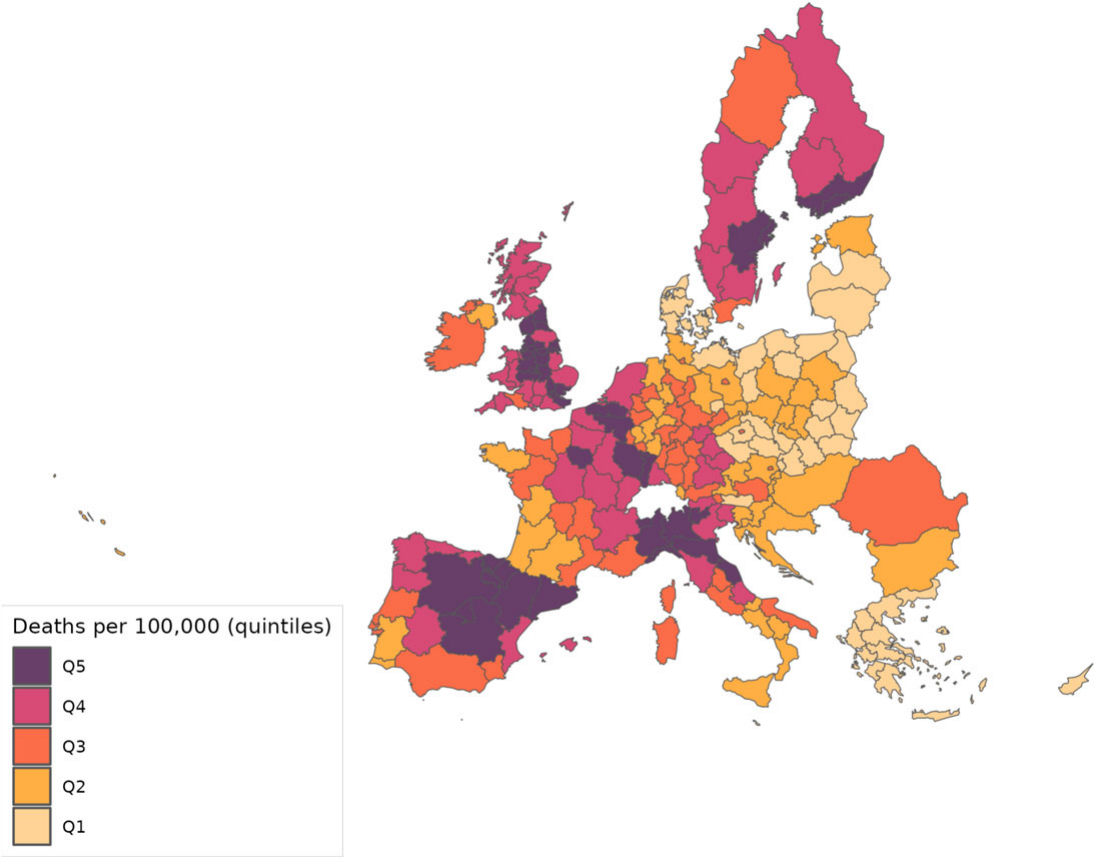
Cumulative deaths per 100,000 inhabitants across regions (July 15, 2020)

Nevertheless, even if the level of death events were larger in continental Europe, the incidence of the disease was actually larger in the UK. We can observe this fact in Fig. 8, which reports the number of deaths per 100,000 inhabitants. In the UK, this ratio reached 1.25, whereas in the EU27, its maximum was a bit less than half that number; in particular, it was 0.61. The map in Fig. 9 shows the cumulative deaths per 100,000 inhabitants up to July 15 by regions. This map shows that most of the regions in the UK were in the fourth or fifth quintiles of the death distribution, similar to northern Italy, and northern Spain.

**Fig. 10:**
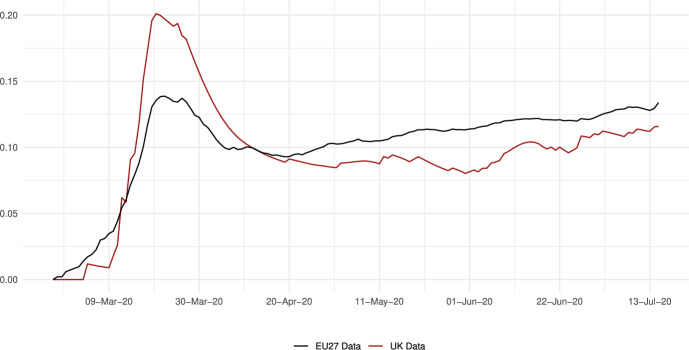
Average daily $\rho _{g}$ in the EU27 and the UK

Figure 10 presents the average value of the parameter $\rho _{g}$ across NUTS2 regions. It is important to remember that this parameter is calibrated as a residual, meaning that its values reflect both the disease ecology and the impact of pandemic-fighting policies. From Fig. 10, we can observe that the probability of infection reached higher values in the UK than in the European Union. The maximum, in particular, was 0.20 on March 21 for the former economy and 0.14 on March 22 for the latter. However, we can also see that the reduction was faster and deeper in the UK than in the EU27. Consequently, policies seem to have been more successful in the UK, maintaining after April 16 a gap in favor of the UK of about 2 percentage points.

**Fig. 11:**
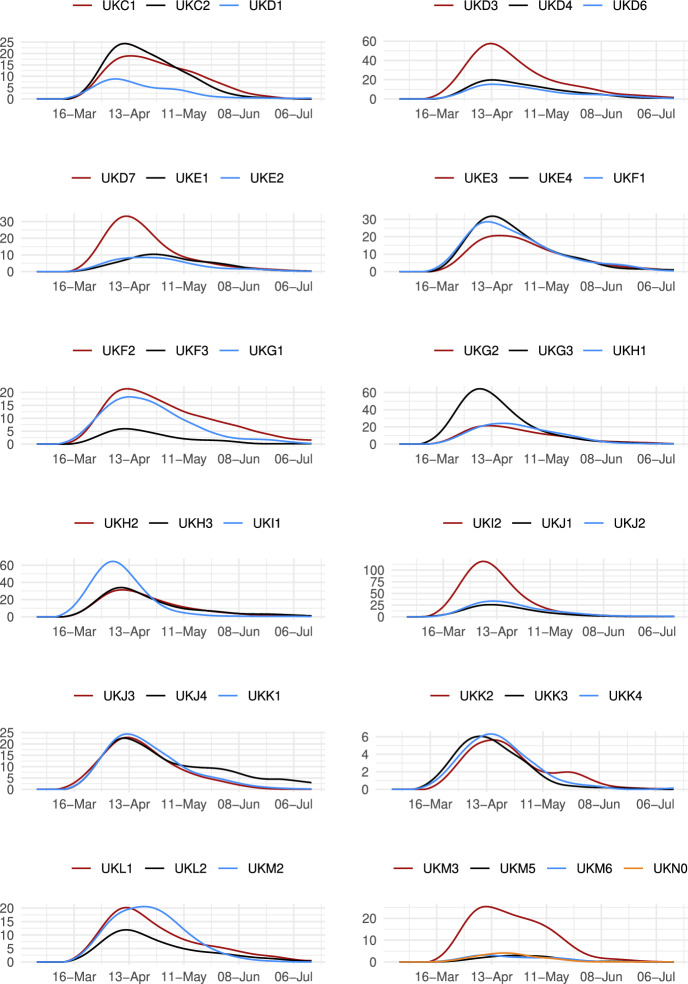
Total daily deaths in the UK NUTS2 regions

Let us now have a more disaggregated view of the UK death data. Figure 11 plots the number of deaths in each of the 37 NUTS2 regions in the UK. The largest number of daily cases was achieved in Inner London-East (UKI2), Greater Manchester (UKD3) and West Midlands (UKG3) with 118, 64 and 57 deaths in one day, respectively. The lowest daily numbers, on the other hand, took place in North Eastern Scotland (UKM5), Highlands and Islands (UKM6) and Northern Ireland (UKN0) with 3, 3 and 4 cases, respectively.

Even though the number of deaths and their relative magnitude per 100,000 inhabitants show a high correlation of 0.561, they do not correlate perfectly. In the second column of results in Table 12, we see that the largest volumes of deaths per 100,000 inhabitants are found in Greater Manchester (UKD3), Cheshire (UKD6), Trees Valley and Durham (UKC1) and West Midlands (UKG3), with rates equal to 93, 90, 87 and 87, respectively. Conversely, the lowest rates are observed in Northern Ireland (UKNO), Dorset and Somerset (UKK2) and Devon (UKK4), where these rates were 6, 19 and 22, respectively.

**Table 12:** Results

	Deaths during first wave		Predicted deaths		Predicted deaths, no restrictions			Predicted deaths with $\rho$ constant		
Code	Total	per 100k	Total	Geo. (%)	Total	Increase (%)	Lives saved	Total	Increase (%)	Lives saved
EU27	133063	28	105801	10.0	107111	1.2	0.06	4519392	4171.6	201
UK	40672	57	30521	9.6	30571	0.2	0.07	1244497	3977.5	1713
UKC1	1037	87	735	9.9	737	0.2	0.14	19544	2559.4	1575
UKC2	1095	76	825	12.7	827	0.3	0.14	22385	2614.1	1491
UKD1	382	77	322	8.7	323	0.0	0.02	6266	1843.1	1192
UKD3	2587	93	1656	9.8	1660	0.3	0.18	63764	3751.7	2226
UKD4	1016	68	761	9.4	762	0.1	0.07	22757	2891.5	1479
UKD6	829	90	509	9.1	505	$-$0.9	$-$0.51	21596	4139.0	2281
UKD7	1283	83	929	9.9	920	$-$1.0	$-$0.60	29037	3025.5	1823
UKE1	546	59	407	11.6	409	0.4	0.17	9983	2350.8	1031
UKE2	464	57	351	11.4	353	0.3	0.14	13933	3864.9	1660
UKE3	1120	81	786	8.6	788	0.2	0.10	24544	3021.0	1710
UKE4	1440	63	1049	11.2	1052	0.2	0.11	46507	4332.6	1976
UKF1	1416	65	976	10.6	977	0.2	0.07	49436	4967.7	2215
UKF2	1250	69	861	8.6	863	0.2	0.08	35559	4027.9	1914
UKF3	262	35	236	10.5	237	0.4	0.12	6493	2651.2	836
UKG1	942	70	651	11.6	651	0.1	0.04	25706	3851.6	1873
UKG2	1121	69	814	9.4	815	0.1	0.04	29792	3559.2	1796
UKG3	2496	87	1735	13.4	1737	0.1	0.06	63339	3550.8	2136
UKH1	1248	50	994	10.2	996	0.1	0.05	41316	4054.8	1617
UKH2	1414	77	1049	9.6	1051	0.1	0.08	27559	2526.9	1439
UKH3	1443	80	990	11.2	992	0.2	0.08	38012	3738.2	2041
UKI1	2063	64	1884	8.7	1891	0.4	0.21	57750	2964.6	1742
UKI2	4155	79	2848	19.5	2849	0.0	0.02	116349	3985.2	2155
UKJ1	1138	48	708	15.7	710	0.3	0.09	63970	8940.0	2652
UKJ2	1544	54	1143	13.5	1144	0.1	0.06	50789	4345.3	1729
UKJ3	1001	51	770	11.8	772	0.3	0.12	31254	3959.3	1544
UKJ4	1254	69	877	7.8	879	0.1	0.07	31519	3492.0	1679
UKK1	1088	44	858	15.8	859	0.1	0.04	41465	4730.1	1641
UKK2	256	19	269	11.1	271	0.8	0.15	12059	4385.8	892
UKK3	247	44	245	8.5	245	0.3	0.15	7414	2931.9	1279
UKK4	262	22	259	16.7	261	0.8	0.17	15164	5755.0	1263
UKL1	975	50	775	9.8	776	0.1	0.05	27118	3398.1	1343
UKL2	576	50	461	9.6	462	0.0	0.01	14975	3145.0	1253
UKM2	1002	24	946	16.9	952	0.6	0.14	53927	5600.0	1269
UKM3	1268	27	1232	10.6	1237	0.4	0.10	82722	6613.1	1729
UKM5	139	28	148	7.8	148	0.3	0.10	4301	2808.2	845
UKM6	150	32	144	9.4	145	0.6	0.20	5097	3431.2	1055
UKN0	163	6	315	14.1	318	0.9	0.11	31096	9773.1	1113

**Fig. 12:**
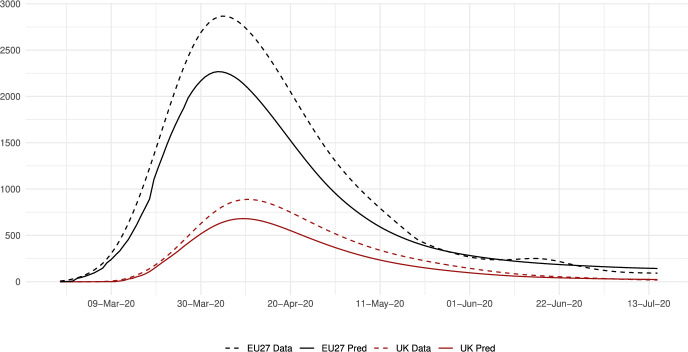
Daily deaths: data versus predictions

Finally in this subsection, we analyze how well the model matches the fatality data. Figure 12 shows that the model predictions in the benchmark scenario follow well the aggregate trend and its changes in the UK and the European Union. Nevertheless, they tend to underestimate the number of deaths. Comparing columns one and three in Table 12, we can see that this results in an error in the predicted total number of deaths of 20.9% and 23.4% for the European Union and the UK, respectively. This is partly due to the method followed to calibrate the parameter $\rho _{g}$, which does not consider the geographic component of the infection (see Appendix B for details).

**Fig. 13:**
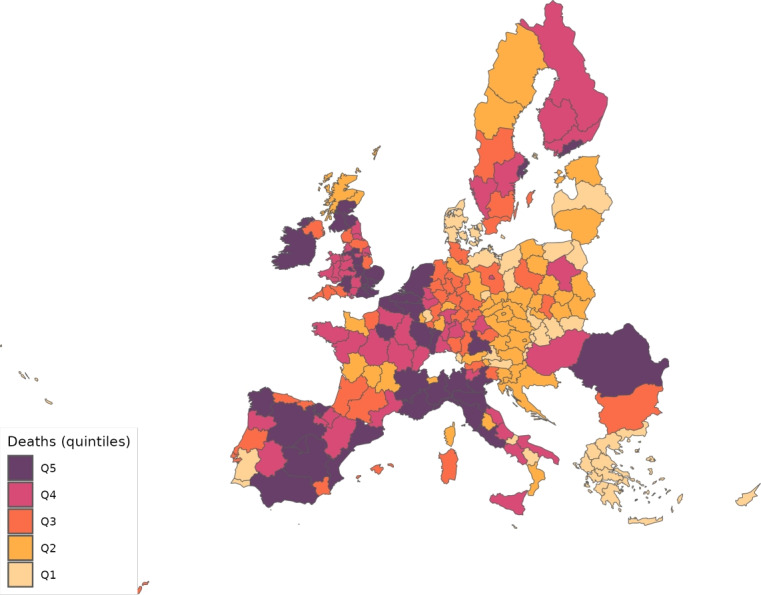
Predicted deaths

Looking now across regions, Fig. 13 shows the distribution of predicted deaths, which can be compared to the actual distribution shown in Fig. 9. Overall, the model fit is good.

### Policy counterfactuals

Our next task is assessing how anti-COVID policy has influenced the number of lives saved. Firstly, we ask: what would have been the cost for the economy in terms of deaths if no policy had been implemented? Secondly, we assess the importance of the geographic component, and the policy cross-effects between the UK and the European Union that it generates. Thirdly, we ask: how many lives could have been saved if all regions had enjoyed the disease ecology and policies implemented in the most successful areas?

#### No-policy scenario

In our model, the implemented policy measures are represented by the evolution of the parameter $\rho _{g}$ and the adjustments to $\alpha ^{j}_{g}$, $L_g$, $d_{ig}^{j}$ and ${\mathfrak {d}}_{ig}^{j}$ in response to mobility restrictions. Unlike $\alpha ^{j}_{g}$, $L_g$, $d_{ig}^{j}$ and ${\mathfrak {d}}_{ig}^{j}$, determining the value of $\rho _{g}$ in the absence of policy measures is not immediate. At the regional level, the parameter $\rho _{g}$ reaches it largest values at the beginning of the infection in the corresponding area, and then goes down due to the policy actions implemented.^17^ However, in general, governments did not react immediately to the first COVID-19 infection cases. The average reaction time varied from a few days to a couple of weeks. Therefore, in order to assess how many additional deaths would have occurred if no policy had been implemented, we keep the parameter $\rho _{g}$ constant at its average over the first ten days during which region *g* reports fatalities. This approach should provide a value of $\rho _{g}$ not significantly affected by anti-COVID policy. Additionally, averaging over 10 days reduces concerns about measurement error.

We assess the impact of changes in $\rho _{g}$ and the adjustments due to the mobility restrictions through two separate exercises. Table 12 in the columns labeled as “Predicted deaths, no restrictions” presents the results of eliminating the adjustments to $\alpha ^{j}_{g}$, $L_g$, $d_{ig}^{j}$ and ${\mathfrak {d}}_{ig}^{j}$, while maintaining the calibrated evolution of $\rho _{g}$. We see that, in this scenario, due to changes in trade patterns, deaths in the EU would have totaled 107,111 instead of the predicted 105,801, and 30,571 instead of 30,571 in the UK, representing an increase of 1.2% and 0.2%, respectively. These figures translate to lives saved of 0.06 and 0.07 per 100,000 inhabitants in the EU27 and the UK, respectively, which are relatively low numbers.

In turn, the columns labeled “Predicted deaths with $\rho$ constant” in Table 12 present the results when $\rho _{g}$ remains unchanged, while $\alpha ^{j}_{g}$, $L_g$, $d_{ig}^{j}$ and ${\mathfrak {d}}_{ig}^{j}$ are adjusted to account for mobility restrictions. Without the policy response of $\rho _{g}$, deaths would have totaled 4,519,392 in the EU and 1,244,497 in the UK, representing an increase of 4,172% and 3,978%, respectively, compared to their benchmark values. In terms of the lives saved per 100,000 inhabitants, the average for the EU27 and the UK equals 202 and 1718, respectively. The impact is now substantial and notably stronger for the UK.

These findings suggest that the primary effect of policy operated through the social component. The impact of mobility restriction on workplace infection, captured by the geographic component, seems to be relatively minor.^18^ Therefore, in the remaining policy counterfactuals, we will focus exclusively on the effects of changes in $\rho$.

**Fig. 14:**
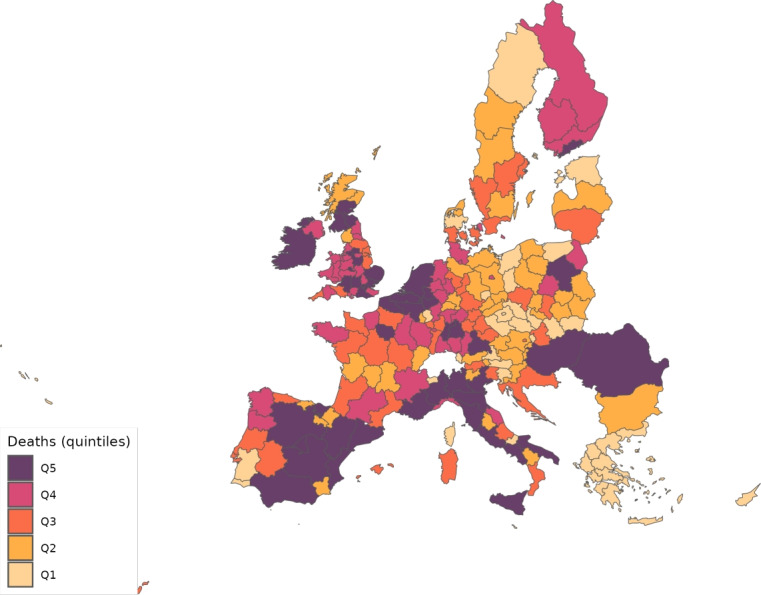
Predicted deaths with $\rho _g$ constant

Figure 14 shows the distribution of predicted deaths across NUTS2 regions when $\rho _g$ remains constant, which can be compared to Fig. 13. We see that the effect is not homogeneous across Europe. For example, some regions in southern Italy and near Madrid in Spain move to upper quintiles of the distribution, whereas most regions surrounding Paris and in southern Sweden move to lower quintiles. This highlights that if anti-COVID policies had not been implemented, the distribution of disease incidence across European regions would have been very different.

Going back to Table 12, there is a relatively high correlation of 0.667 between the number of deaths in the data and the lives saved by policies across NUTS2 regions. For example, focusing on the UK, the largest effect is found in Berkshire, Buckinghamshire and Oxfordshire (UKJ1) where 2652 lives per 100,000 inhabitants were saved by the policy measures. Other areas where more than 2000 lives per 100,000 inhabitants were saved include Cheshire (UKD6), Derbyshire and Nottinghamshire (UKF1), Greater Manchester (UKD3), Inner London-East (UKI2), West Midlands (UKG3) and Essex (UKH3). The smallest impact, in turn, is found in Lincolnshire (UKF3), North Eastern Scotland (UKM5) and Dorset and Somerset (UKK2), where the lives saved are 836, 845 and 892 per 100,000 inhabitants, respectively.

#### Trade and policy cross-effects

In this paper, we are particularly interested in measuring the impact of economic links on the pandemic. Let us start by examining the weight of trade with different locations in each of the UK regions. Table 13 indicates that the largest share in trade is UK based, with intra-region and cross-UK-region trade accounting for between $83.0\%$ and $96.2\%$ of total trade. The dominance of either intra-region or inter-region trade varies widely across regions. For example, Cheshire (UKD6) shows the largest reliance on domestic trade, with $59.6\%$ occurring within the region and $25.4\%$ with flows to other UK areas. In contrast, Lincolnshire (UKF3) relies less on intra-region flows, with only $28.7\%$, while its inter-regional trade with the rest of the UK accounts for $66.1\%$ of total trade.

**Table 13:** Intra- and inter-regional trade for the UK NUTS2 regions

Region code	Region name	Domestic	Rest of UK	EU27	ROW
UKC1	Tees Valley and Durham	0.308	0.605	0.068	0.019
UKC2	Northumberland and Tyne and Wear	0.376	0.534	0.077	0.014
UKD1	Cumbria	0.356	0.579	0.060	0.005
UKD3	Greater Manchester	0.408	0.535	0.044	0.013
UKD4	Lancashire	0.305	0.638	0.050	0.007
UKD6	Cheshire	0.596	0.254	0.043	0.107
UKD7	Merseyside	0.562	0.339	0.061	0.038
UKE1	East Yorkshire and Northern Lincolnshire	0.380	0.565	0.049	0.006
UKE2	North Yorkshire	0.347	0.604	0.042	0.006
UKE3	South Yorkshire	0.308	0.617	0.071	0.004
UKE4	West Yorkshire	0.382	0.559	0.051	0.007
UKF1	Derbyshire and Nottinghamshire	0.333	0.613	0.044	0.011
UKF2	Leicestershire, Rutland and Northamptonshire	0.392	0.552	0.047	0.010
UKF3	Lincolnshire	0.287	0.660	0.047	0.005
UKG1	Herefordshire, Worcestershire and Warwickshire	0.358	0.588	0.044	0.010
UKG2	Shropshire and Staffordshire	0.340	0.622	0.034	0.004
UKG3	West Midlands	0.385	0.529	0.061	0.024
UKH1	East Anglia	0.413	0.506	0.047	0.034
UKH2	Bedfordshire and Hertfordshire	0.354	0.545	0.071	0.029
UKH3	Essex	0.310	0.593	0.071	0.026
UKI1	Inner London-West	0.452	0.388	0.079	0.081
UKI2	Inner London-East	0.334	0.497	0.075	0.094
UKJ1	Berkshire, Buckinghamshire and Oxfordshire	0.451	0.449	0.062	0.038
UKJ2	Surrey, East and West Sussex	0.357	0.504	0.070	0.069
UKJ3	Hampshire and Isle of Wight	0.429	0.503	0.040	0.027
UKJ4	Kent	0.345	0.566	0.056	0.033
UKK1	Gloucestershire, Wiltshire and Bristol/Bath area	0.396	0.529	0.041	0.033
UKK2	Dorset and Somerset	0.304	0.635	0.039	0.022
UKK3	Cornwall and Isles of Scilly	0.359	0.586	0.045	0.010
UKK4	Devon	0.346	0.594	0.038	0.022
UKL1	West Wales and The Valleys	0.303	0.621	0.046	0.029
UKL2	East Wales	0.331	0.627	0.035	0.007
UKM2	Eastern Scotland	0.401	0.533	0.032	0.034
UKM3	South Western Scotland	0.430	0.507	0.032	0.030
UKM5	North Eastern Scotland	0.616	0.343	0.035	0.006
UKM6	Highlands and Islands	0.398	0.545	0.047	0.010
UKN0	Northern Ireland (UK)	0.426	0.493	0.048	0.033

Trade flows with the European Union also vary significantly across UK regions. Inner London-East and West (UKI1 and UKI2) show the largest shares of $7.6\%$ and $7.9\%$, respectively, while Eastern Scotland (UKM2) has the lowest share at $3.2\%$.

These results suggest that trade across regions may have had an important effect on the spread of the disease. A first assessment of this hypothesis is provided by the fourth column of results in Table 12. It gives the percentage contribution of the Geographic component in equation (46) to the generation of infected individuals, and therefore, to the number of fatalities. Recall that the geographic component collects the impact of all economic activity. Its weight in total deaths averages $10.0\%$ in the European Union and $9.6\%$ in the UK. Across UK regions, it reaches the highest values of $19.5\%$ in Inner London-East (UKI2), $16.9\%$ for Eastern Scotland (UKM2) and $16.7\%$ for Devon (UKK4). The smallest values are found in Kent (UKJ4) and North Eastern Scotland (UKM5) both with $7.8\%$.

**Fig. 15:**
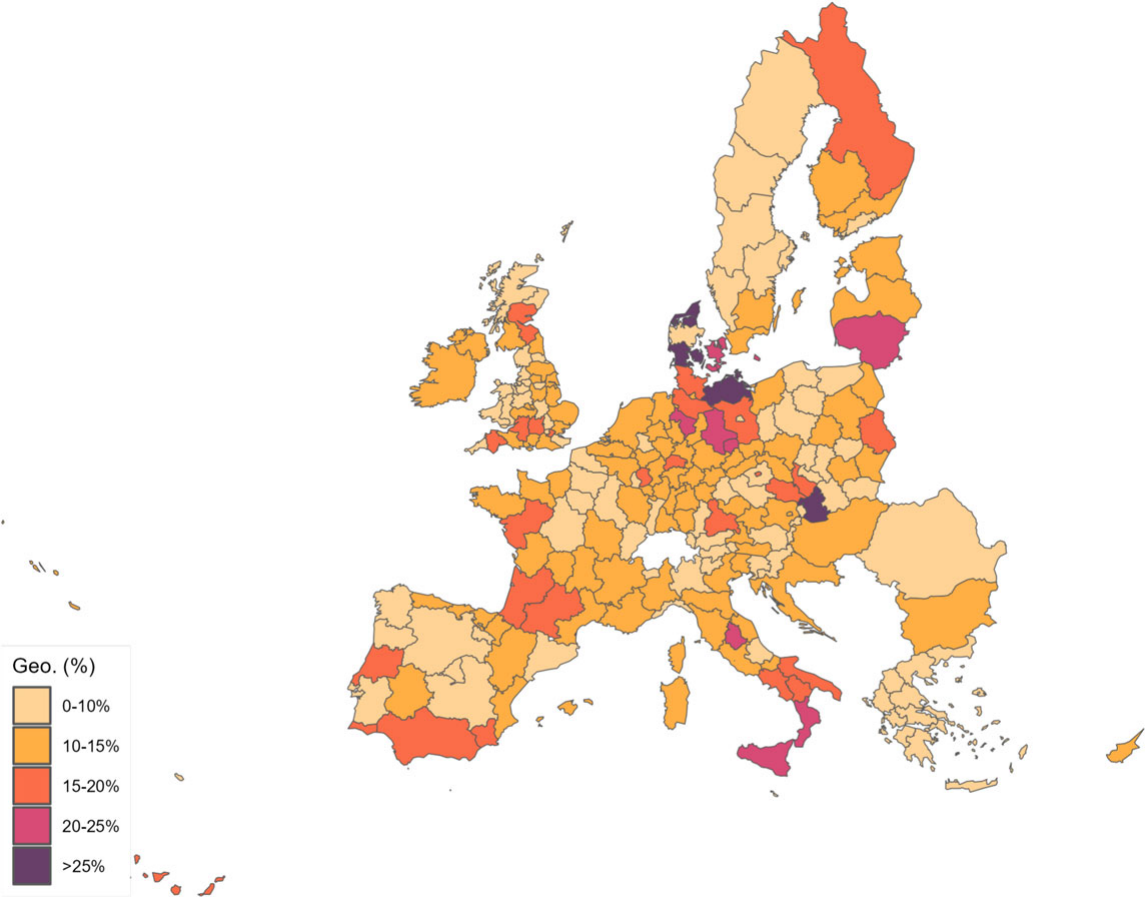
Geographic component

The distribution of the geographic component across all regions that compose our sample can be seen in Fig. 15. The regions that seem most affected by the geographic component are in Denmark, northern Germany, southern Italy, and southern Spain. While these areas are not the most heavily affected by the pandemic, it is important to note that the geographic component depends not only on trade flows, but also on the disease-transmission probability in other areas.

**Fig. 16:**
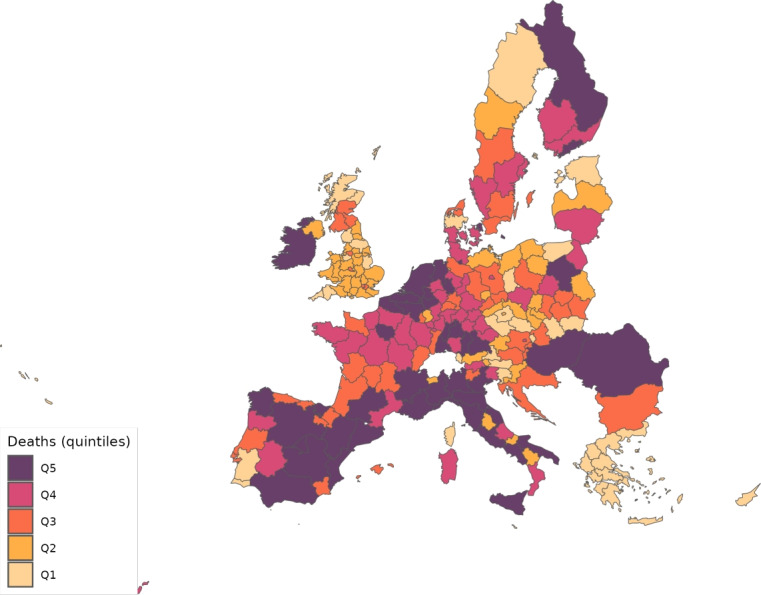
Counterfactual—predicted deaths with $\rho _g$ constant (EU27)

One type of effect that runs fully through the geographic component is the impact of a policy implemented in one region on the prevalence of the disease in a different region. In the next set of experiments, we examine the policy cross-effects between the EU27 and the UK. We first consider the impact of maintaining $\rho _{g}$ constant in the EU27 but allowing it to vary in the UK. As before, this constant value equals the region-specific average over the first ten days of virus incidence. This will give us an idea of the impact of the applied European Union anti-COVID policies on UK prevalence. The first three columns in Table 14 provide the results of this exercise, and Fig. 16 plots the distribution across regions.

**Table 14:** Additional results with $\rho$ constant

		Predicted deaths with $\rho$ constant in EU27			Predicted deaths with $\rho$ constant in UK		
Code	Name	Total	Increase (%)	Lives saved	Total	Increase (%)	Lives saved
EU27	European Union	4459274	4115	198	157508	49	2
UK	United Kingdom	55919	83	36	1230709	3932	1694
UKC1	Tees Valley and Durham	1169	59	36	19275	2523	1552
UKC2	Northumberland and Tyne and Wear	1349	64	36	22011	2569	1465
UKD1	Cumbria	625	94	61	6092	1789	1157
UKD3	Greater Manchester	2220	34	20	63595	3741	2220
UKD4	Lancashire	1203	58	30	22475	2854	1460
UKD6	Cheshire	706	39	21	21541	4128	2275
UKD7	Merseyside	1376	48	29	28834	3004	1810
UKE1	East Yorkshire and Northern Lincolnshire	799	96	42	9563	2248	985
UKE2	North Yorkshire	675	92	39	13746	3812	1637
UKE3	South Yorkshire	1210	54	31	24318	2992	1694
UKE4	West Yorkshire	1573	50	23	46285	4311	1966
UKF1	Derbyshire and Nottinghamshire	1611	65	29	49254	4949	2207
UKF2	Leicestershire, Rutland and Northamptonshire	1485	72	34	35307	3999	1900
UKF3	Lincolnshire	634	169	53	6171	2515	793
UKG1	Herefordshire, Worcestershire and Warwickshire	1108	70	34	25508	3821	1858
UKG2	Shropshire and Staffordshire	1274	56	28	29585	3534	1783
UKG3	West Midlands	2406	39	23	63111	3538	2128
UKH1	East Anglia	2120	113	45	40751	3998	1595
UKH2	Bedfordshire and Hertfordshire	1718	64	36	27154	2488	1417
UKH3	Essex	1933	95	52	37713	3708	2025
UKI1	Inner London-West	2839	51	30	57462	2949	1733
UKI2	Inner London-East	4276	50	27	115866	3968	2146
UKJ1	Berkshire, Buckinghamshire and Oxfordshire	1326	87	26	63843	8922	2647
UKJ2	Surrey, East and West Sussex	2115	85	34	50294	4302	1712
UKJ3	Hampshire and Isle of Wight	1468	91	35	30858	3908	1524
UKJ4	Kent	1769	102	49	31155	3451	1659
UKK1	Gloucestershire, Wiltshire and Bristol/Bath area	1423	66	23	41054	4682	1624
UKK2	Dorset and Somerset	986	267	54	11505	4179	850
UKK3	Cornwall and Isles of Scilly	618	153	67	7217	2851	1244
UKK4	Devon	757	192	42	14772	5604	1229
UKL1	West Wales and The Valleys	1494	93	37	26629	3335	1319
UKL2	East Wales	854	85	34	14688	3083	1228
UKM2	Eastern Scotland	2336	147	33	52623	5462	1237
UKM3	South Western Scotland	3532	187	49	81768	6536	1709
UKM5	North Eastern Scotland	407	175	53	4081	2659	800
UKM6	Highlands and Islands	517	258	79	4809	3232	994
UKN0	Northern Ireland (UK)	2012	539	61	29794	9360	1066

Without the policies implemented in the EU27, Table 14 indicates that the number of deaths in the UK would have been 83% larger. The lives saved in the UK by these policies amount to 25,398, or 36 per 100,000 inhabitants.

By region, Highlands and Islands (UKM6) benefited the most, with lived saved per 100,000 inhabitants equal to 79. Following closely, Cornwall and Isles of Scilly (UKK3), Cumbria (UKD1), Northern Ireland (UKN0), North Eastern Scotland (UKM5) and Lincolnshire (UKF3) each saved more than 50 lives. The areas that benefited the least were Greater Manchester (UKD3), West Yorkshire (UKE4), Gloucestershire, Wiltshire and Bristol/Bath area (UKK1) and West Midlands (UKG3), where the EU27 policies saved fewer than 25 lives. Nevertheless, as Fig. 16 shows, the UK regions would have suffered much less compared to the EU, since most of the regions would remain in the first or second quintiles of the distribution. However, areas of Inner London would still remain in the third quintile of the distribution.

**Fig. 17:**
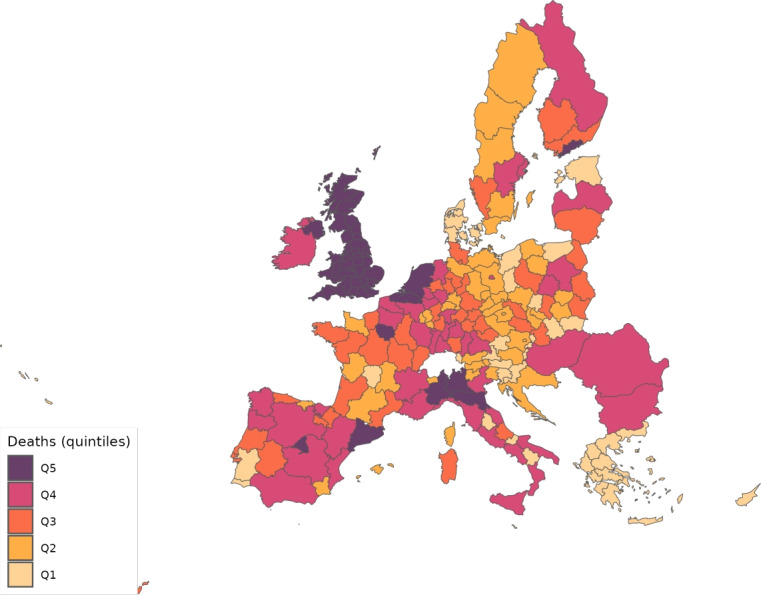
Counterfactual—predicted deaths with $\rho _g$ constant (UK)

In our second cross-effects exercise, we focus on the opposite scenario: we assume that $\rho _{g}$ changes in EU27 regions but remains constant in UK regions. The last three columns in Table 14 provides the results, and Fig. 17 plots the distribution across regions. They imply that UK anti-COVID measures saved 51,706 lives in the European Union, which represents 2 lives per 100,000 inhabitants. As expected, the disease would have put all regions in the UK in the upper quintiles of the distribution. Note, however, that European capitals and highly connected regions would still fall in the upper quintiles.

Using predictions from this last exercise, we can also analyze the impact of the UK anti-COVID policy on UK regions. This impact is much larger than for the EU27. Specifically, they saved a total of 1,200,188 lives or 1,694 per 100,000 inhabitants. Berkshire, Buckinghamshire and Oxfordshire (UKJ1) were the most benefitted, with 2,647 lives saved per 100,000 inhabitants. It was followed by Cheshire (UKD6), Greater Manchester (UKD3), Derbyshire and Nottinghamshire (UKF1), Inner London-East (UKI2), West Midlands (UKG3) and Essex (UKH3). All of them with more than 2,000 lives saved by the fight against COVID-19 in the UK during the first wave. At the bottom of this ranking, we have Lincolnshire (UKF3), North Eastern Scotland (UKM5) and Dorset and Somerset (UKK2) with 793, 800 and 850 lives saved per 100,000 inhabitants, respectively.

Interestingly, comparing results from the last two experiments, we find that the correlation across UK regions between the lives saved by EU27 and by UK policies is significantly positive, equal to 0.66. The same correlation across EU27 regions is also substantial, equal to 0.38. These positive correlations underscore the importance of considering trade links for the effective design of infectious disease containment policies.

#### Best performing regions

In our last counterfactual, we consider how the pandemic might have evolved if all regions had followed the policies of the best performing regions. However, there is no single clear metric for determining the *“best performer.”* One possibility is the region that most effectively reduces the disease-transmission probability, $\rho _{g}$. Another is the region that achieves the greatest reduction in the number of deaths. We will combine these two metrics to find the best performer and impose the evolution of $\rho _g$ implied by the best performing region on all European regions. Since anti-COVID policy measures were adjusted based on the reported number of daily deaths, with some places removing restrictions earlier than others, we will identify the best performers in both the first two months of the analyzed period (days 1 to 61) and the second part (days 62 to 142).^19^

We start by searching for the areas that most rapidly reduced the disease-transmission probability, $\rho _g$, following its first maximum, which typically occurs on the first day with reported deaths. To compute the implied decrease, we use the ratio of the average $\rho _g$ for the first 5 days of the 2-month period to the average $\rho _g$ for the last 5 days, in order to decrease potential measurement errors. After that, we simulate the model using the implied daily reductions in $\rho _g$ from each of the top 10 areas identified by this ratio. We apply the same daily rate of decline in $\rho _g$ to all regions, starting from their calibrated region-specific value in the day when deaths from COVID-19 were first reported. Thus, the initial levels of $\rho _g$ remain unchanged, and only the speed of the decrease is altered. Finally, we calculate which reduction in $\rho _g$ results in the most lives saved.

When we focus on the first two months, Northumberland and Tyne and Wear (UKC2), a fourth-quintile-density UK region (see Fig. 1), ranks first according to this ratio, reducing $\rho _g$ in 61 days to 11.5% of its initial value. It is followed by the Netherlands (NL), which reduced it to 12.9% of the initial value. Within the ten best performers, we find five UK regions, further emphasizing that the UK was more effective in the fight against COVID that other European countries. However, within the top 10 regions, we find Oberbayern (DE21), a fourth-quintile-density region in Germany. The evolution of $\rho _g$ in Oberbayern, which decreased to 18.0% of the initial value, is actually the one that results in the most lives saved in Europe in our simulations, thus representing our chosen most effective region in the fight against COVID-19.

When we focus on the period from day 62 to 142, interestingly, the ten areas that performed better in the first 61 days no longer appear among the top 10 performers. Additionally, we find now only three UK regions in this top 10, suggesting that the UK may have lifted restrictions too early. Nevertheless, South Yorkshire (UKE3), a top-quintile-density UK region, is the best performer, with $\rho _g$ on day 142 at 35.9% of its value on day 62 after its peak. It is followed by Toscana (ITI1) in Italy, with a reduction to 36.1%. South Yorkshire (UKE3) performs the best in terms of the ratio and also the implied prevented deaths.

**Fig. 18:**
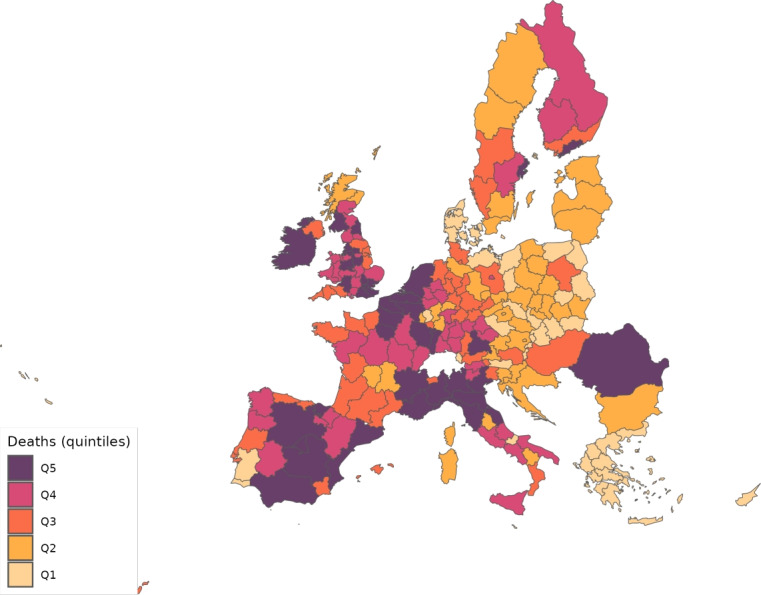
Counterfactual—predicted deaths with best regions

Therefore, we apply the daily reduction ratio implied by Oberbayern (DE21) from day 1 to day 61 of reported deaths, and the daily reduction ratio implied by South Yorkshire (UKE3) from day 62 to the end of the first wave, to the $\rho _g$ of each European region in our sample. Results are contained in Table 15 and Fig. 18. Table 15 indicates that the European Union implemented the policies followed by the best performers, and 37% of deaths could have been saved, which amount to a total of 38,883 saved lives.^20^ For the UK, the percentage reduction of deaths would have been slightly higher, a 42%, due to the larger incidence of the disease, and the total number of lives saved would equal 12,752.

**Table 15:** Results with best performing regions

Code	Region name	Total deaths	Lives saved	Decrease in deaths (%)
EU27	European Union	66919	38883	37
UK	United Kingdom	17769	12752	42
UKC1	Tees Valley and Durham	446	289	39
UKC2	Northumberland and Tyne and Wear	578	247	30
UKD1	Cumbria	215	107	33
UKD3	Greater Manchester	795	860	52
UKD4	Lancashire	474	286	38
UKD6	Cheshire	218	291	57
UKD7	Merseyside	679	250	27
UKE1	East Yorkshire and Northern Lincolnshire	151	257	63
UKE2	North Yorkshire	169	183	52
UKE3	South Yorkshire	425	362	46
UKE4	West Yorkshire	667	382	36
UKF1	Derbyshire and Nottinghamshire	536	439	45
UKF2	Leicestershire, Rutland and Northamptonshire	421	440	51
UKF3	Lincolnshire	156	80	34
UKG1	Herefordshire, Worcestershire and Warwickshire	303	347	53
UKG2	Shropshire and Staffordshire	484	330	41
UKG3	West Midlands	1116	619	36
UKH1	East Anglia	446	549	55
UKH2	Bedfordshire and Hertfordshire	719	330	31
UKH3	Essex	686	305	31
UKI1	Inner London-West	1254	630	33
UKI2	Inner London-East	2090	758	27
UKJ1	Berkshire, Buckinghamshire and Oxfordshire	294	413	58
UKJ2	Surrey, East and West Sussex	625	517	45
UKJ3	Hampshire and Isle of Wight	362	408	53
UKJ4	Kent	483	394	45
UKK1	Gloucestershire, Wiltshire and Bristol/Bath area	482	376	44
UKK2	Dorset and Somerset	144	125	47
UKK3	Cornwall and Isles of Scilly	127	118	48
UKK4	Devon	131	128	49
UKL1	West Wales and The Valleys	436	340	44
UKL2	East Wales	265	197	43
UKM2	Eastern Scotland	467	479	51
UKM3	South Western Scotland	670	562	46
UKM5	North Eastern Scotland	59	89	60
UKM6	Highlands and Islands	81	63	44
UKN0	Northern Ireland (UK)	115	200	63

Across UK regions, Northern Ireland (UKN0), East Yorkshire and Northern Lincolnshire (UKE1) and North Eastern Scotland (UKM5) would have experienced the most lives saved, with 63%, 63% and 60% fewer deaths, respectively. In contrast, Northumberland and Tyne and Wear (UKC2), Merseyside (UKD7) and Inner London-East (UKI2), with reductions to 30%, 27% and 27%, respectively, would have experienced the smallest percentage of lives saved.

## Conclusion

We have built a spatial model of trade with supply-chain links across NUTS2 European regions to try to understand the effect of economic links and policies in the spread of the COVID-19 pandemic during the first wave, which goes from February 25 to July 15, 2020. We have primarily focused on this effect within the UK in comparison with the rest of NUTS2 regions in the EU.

During that period, the incidence of the disease was larger in the UK than in the European Union. However, we find that the efforts to reduce infection rates were more successful in the UK than in the European Union. More importantly, without the policy reaction in Europe, the number of deaths during the first wave of the pandemic would have been about 4,410,000 larger in the European Union and about 1,210,000 higher in the UK.

In terms of the lives saved per 100,000 inhabitants, the average for the EU27 and the UK equals 201 and 1,713, respectively. On average, the largest gains were in areas where the volume of deaths was higher, like Berkshire, Buckinghamshire and Oxfordshire, Cheshire, Greater Manchester, Inner London-East, West Midlands and Essex. However, these regions still remain in the upper quintiles of the distribution of deaths.

Our analysis reveals that, on average, the percentage contribution of the geographic component, which reflects the impact of economic activity, accounts for $10\%$ of COVID-19 fatalities. Hence, while social interaction represents the primary channel through which policy impacted disease transmission, the importance of economic activity on the deaths caused by the pandemic is also significant. This channel is particularly important for regions with substantial economic interactions with other regions, even if they do not have high internal infection rates.

Another interesting finding we report is that the number of deaths in the UK in the absence of anti-COVID-19 measures in the European Union would have been 83% larger; specifically, they saved about 36 lives per 100,000 inhabitants in the UK. In turn, UK anti-COVID-19 measures saved 51,706 lives in the European Union, which represents 2 lives per 100,000 inhabitants. This asymmetric difference is in part due to the asymmetric trade intensity across regions.

Finally, we have identified Oberbayern in Germany as the single most effective region in the fight against COVID-19 during the first two months after deaths due to COVID were reported. However, due to policy adjustments that occurred throughout the pandemic, South Yorkshire in the UK was the most effective region at keeping the disease-transmission probability low and further reducing it after these initial two months. Furthermore, if all European regions had applied the anti-COVID policies implemented by these two best performers, 37% of deaths could have been prevented in the European Union and 42% in the UK, amounting to a total of 51,635 fewer deaths in Europe.

## The basic reproduction number in our SVIRCF model

Following Heffernan et al. (2005), we can write the equation for infected individuals in matrix form as:

53 $$\begin{aligned} {\textbf{I}}^{\prime }= &  \left( {\mathbb {I}} + {\textbf{F}} - {\textbf{D}}\right) {\textbf{I}}; \end{aligned}$$

where ${\mathbb {I}}$ is the identity matrix, ${\textbf{I}}^{\prime }$ is the vector of infections in each location at time $t+1$, and ${\textbf{F}}$ and ${\textbf{D}}$ are defined as

$$\begin{aligned} {\textbf{F}}= &  \left( {\begin{smallmatrix} (1-\kappa )\rho _{1}\frac{S_{1}}{N_{1}} + \kappa \Lambda _{1}\rho _{1}{\tilde{X}}_{11}\frac{S_{1}}{N_{1}} & \cdots & \kappa \Lambda _{g}\rho _{g}{\tilde{X}}_{g1}\frac{S_{1}}{N_{g}} & \cdots & \kappa \Lambda _{G}\rho _{G}{\tilde{X}}_{G1}\frac{S_{1}}{N_{G}}\\ \vdots & \ddots & \cdots & \cdots & \vdots \\ \kappa \Lambda _{1}\rho _{1}{\tilde{X}}_{1g}\frac{S_{g}}{N_{1}} & \cdots & (1-\kappa )\rho _{g}\frac{S_{g}}{N_{g}}+\kappa \Lambda _{g}\rho _{g}{\tilde{X}}_{gg}\frac{S_{g}}{N_{g}} & \cdots & \kappa \Lambda _{G}\rho _{G}{\tilde{X}}_{Gg}\frac{S_{g}}{N_{G}}\\ \vdots & \cdots & \cdots & \ddots & \vdots \\ \kappa \Lambda _{1}\rho _{1}{\tilde{X}}_{1G}\frac{S_{G}}{N_{1}} & \cdots & \kappa \Lambda _{g}\rho _{g}{\tilde{X}}_{gG}\frac{S_{G}}{N_{g}} & \cdots & (1-\kappa )\rho _{G}\frac{S_{G}}{N_{G}}+\kappa \Lambda _{G}\rho _{G}{\tilde{X}}_{GG}\frac{S_{G}}{N_{G}} \end{smallmatrix}} \right) \\ {\textbf{D}}= &  \begin{pmatrix} \varphi & \cdots & 0 & \cdots & 0\\ \vdots & \ddots & \cdots & \ddots & \vdots \\ 0 & \cdots & \varphi & \cdots & 0\\ \vdots & \ddots & \cdots & \ddots & \vdots \\ 0 & \cdots & 0 & \cdots & \varphi \end{pmatrix} \end{aligned}$$

For the two region case, these matrices are given by

$$\begin{aligned} {\textbf{F}}= &  \left( {\begin{smallmatrix} (1-\kappa )\rho _{1}\frac{S_{1}}{N_{1}}+\kappa \Lambda _{1}\rho _{1}{\tilde{X}}_{11}\frac{S_{1}}{N_{1}} & \kappa \Lambda _{2}\rho _{2}{\tilde{X}}_{21}\frac{S_{1}}{N_{2}} \\ \kappa \Lambda _{1}\rho _{1}{\tilde{X}}_{12}\frac{S_{2}}{N_{1}} & (1-\kappa )\rho _{2}\frac{S_{2}}{N_{2}}+\kappa \Lambda _{2}\rho _{2}{\tilde{X}}_{22} \frac{S_{2}}{N_{2}} \end{smallmatrix}}\right) \\ {\textbf{D}}= &  \begin{pmatrix} \varphi & 0\\ 0 & \varphi \end{pmatrix} \end{aligned}$$

Let us focus on the simplest case of two regions for which the components of ${\tilde{X}}_{gi}$ do not change over time, neither the parameters regarding the disease ecology. In addition, assume that $S_{m,t}=N_{m,t}$, and there is no vaccine available. Then, we have that the basic reproduction number ${\mathcal {R}}_{0}$ is given by the largest eigenvalue of matrix ${\textbf{B}}={\textbf{F}}{\textbf{D}}^{-1}$. Matrix ${\textbf{B}}$ is given by

$$\begin{aligned} {\textbf{B}} = \begin{pmatrix} \displaystyle \frac{{\tilde{X}}_{11}\kappa \rho _1\Lambda _1+\rho _1\left( 1 - \kappa \right) }{\varphi } & \displaystyle \frac{{\tilde{X}}_{21}\kappa \rho _2\Lambda _2}{\varphi } \\ \displaystyle \frac{{\tilde{X}}_{12}\kappa \rho _1\Lambda _1}{\varphi } & \displaystyle \frac{{\tilde{X}}_{22}\kappa \rho _2\Lambda _2+\rho _2\left( 1 - \kappa \right) }{\varphi }. \end{pmatrix} \end{aligned}$$

Furthermore, let us assume that $\rho _1 = \rho _2 = \rho$ and $\Lambda _1 = \Lambda _2 = \Lambda$,^21^ then the basic reproduction number is given by

$$\begin{aligned} {\mathcal {R}}_{0}=\frac{\kappa \rho \Lambda \sqrt{{\tilde{X}}_{11}^{2}-2{\tilde{X}}_{11}{\tilde{X}}_{22}+4{\tilde{X}}_{12}{\tilde{X}}_{21}+{\tilde{X}}_{22}^{2}}}{2\varphi } + \frac{\rho \left( {\tilde{X}}_{11}\kappa \Lambda +{\tilde{X}}_{22} \kappa \Lambda - 2\kappa + 2\right) }{2\varphi } \end{aligned}$$

which increases with trade integration, since the partial derivatives are increasing in the trade share with the opposite region.

$$\begin{aligned} \frac{\partial {\mathcal {R}}_{0}}{\partial {\tilde{X}}_{12}}&= \frac{\kappa \rho \Lambda {\tilde{X}}_{21}}{\varphi \sqrt{{\tilde{X}}_{11}^{2}-2{\tilde{X}}_{11}{\tilde{X}}_{22}+4{\tilde{X}}_{12}{\tilde{X}}_{21} + {\tilde{X}}_{22}^{2}}}> 0 \\ \frac{\partial {\mathcal {R}}_{0}}{\partial {\tilde{X}}_{21}}&= \frac{\kappa \rho \Lambda {\tilde{X}}_{12}}{\varphi \sqrt{{\tilde{X}}_{11}^{2}-2{\tilde{X}}_{11}{\tilde{X}}_{22}+4{\tilde{X}}_{12}{\tilde{X}}_{21}+{\tilde{X}}_{22}^{2}}} > 0 \end{aligned}$$

## Parameters for the evolution of the disease

In order to calibrate $\{\rho _{gt}\}_{g=1}^{G}$, we follow the method in Fernández-Villaverde and Jones (2022) and recover the parameter from deaths numbers. In addition, to ameliorate possible mismeasurement problems, like for example underreporting during weekends, we first smooth those daily deaths series using a moving average of seven days and then a Hodrick–Prescott filter with smoothing parameter 850.

This calibration method is applied to our case as follows. Let us add a time index (*t*) to the different variables for mathematical convenience. Additionally, let us take the convention that $Z_{t}$ provides the value of an arbitrary variable *Z* at the end of period *t*, and that $\Delta Z_{t+1}=Z_{t+1}-Z_{t}$.^22^ Define also $f_{gt+1}\equiv \Delta F_{gt+1}$, that is, the (smoothed) number of people that died on day $t+1$ in region *g*. For the initial waves of the pandemic, in which there was no vaccine available, we assume $\lambda _{g}=0$ for all regions.

From Eq. (48e), we can solve for $R_{gt}$ in terms of daily deaths as

54 $$\begin{aligned} R_{gt}=\frac{f_{gt+1}}{\delta \xi }, \end{aligned}$$

which then implies

55 $$\begin{aligned} \Delta R_{gt+1}=\frac{\Delta f_{gt+2}}{\delta \xi }. \end{aligned}$$

Combining Eqs. (48c) and (55), we can express infected individuals today as a function of future daily fatalities:

56 $$\begin{aligned} I_{gt}=\frac{1}{\delta \varphi }\left( \frac{\Delta f_{gt+2}}{\xi } + f_{gt+1}\right) \end{aligned}$$

which implies

57 $$\begin{aligned} \Delta I_{gt+1}=\frac{1}{\delta \varphi }\left( \frac{\Delta f_{gt+3} - \Delta f_{gt+2}}{\xi }+\Delta f_{gt+2}\right) . \end{aligned}$$

Using the ratio of (57)–(56), the growth rate of the infected cases can be obtained as:

58 $$\begin{aligned} \frac{\Delta I_{gt+1}}{I_{gt}}=\frac{1/\xi (\Delta f_{gt+3} - \Delta f_{gt+2}) + \Delta f_{gt+2}}{1/\xi \Delta f_{gt+2}+f_{gt+1}}. \end{aligned}$$

Next, equation (45), letting $G_{gt}(I_{it})$ denote the geographic component in equation (46), delivers

$$\begin{aligned} (1-\kappa )\rho _{gt}+\frac{\kappa G_{gt}(I_{it})N_{gt}}{I_{gt}} = \frac{N_{gt}}{S_{gt}}\left( \frac{\Delta I_{gt+1}}{I_{gt}}+\varphi \right) . \end{aligned}$$

We can substitute (56) and (58) in previous expression to get

59 $$\begin{aligned} &  (1-\kappa )\rho _{gt}+\kappa G_{gt}(I_{it})\frac{\delta \varphi N_{gt}}{\left( \frac{\Delta f_{gt+2}}{\xi }+f_{gt+1}\right) } \nonumber \\ &  \quad =\frac{N_{gt}}{S_{gt}}\left( \frac{1/\xi (\Delta f_{gt+3}-\Delta f_{gt+2})+\Delta f_{gt+2}}{1/\xi \Delta f_{gt+2}+f_{gt+1}} + \varphi \right) . \end{aligned}$$

To get an expression for the evolution of the susceptible as a function of the fatalities, we can use (48a), (46), and (56) to obtain the law of motion for this variable as:

60 $$\begin{aligned} &  S_{gt+1}=S_{gt}\Bigg \{1-\lambda _{gt}-(1-\kappa )\frac{\rho _{gt}}{\delta \varphi N_{gt}}\left( \frac{\Delta f_{gt+2}}{\xi }+f_{gt+1}\right) \nonumber \\ &  \quad + \kappa \left( \sum _{i\in G}{\tilde{X}}_{ig}\Lambda _i \rho _{it} \frac{1}{\delta \varphi N_{it}} \left( \frac{\Delta f_{it+2}}{\xi } + f_{it+1} \right) \right) \Bigg \}+\alpha ^{C}C_{gt}+\alpha ^{V}V_{gt}. \end{aligned}$$

Note we also need to include the law of motion for vaccinated and recovered individuals which from (48b) and substituting equation (54) into (48d) yields

61 $$\begin{aligned} V_{gt+1}=(1-\alpha ^{V})V_{gt}+\lambda _{gt}S_{gt} \end{aligned}$$

62 $$\begin{aligned} C_{gt+1}=(1-\alpha ^{C})C_{gt}+\frac{1-\delta }{\delta }f_{gt+1} \end{aligned}$$

Finally, we need initial values for $\{I_{g0},S_{g0},N_{g0}\}_{g=1}^{G}$. For the stock of fatalities, recovered and vaccinated, this value is zero, that is, $F_{g0}=C_{g0}=V_{g0}=0$. Knowing the number of fatalities in the next two periods, we then obtain $I_{g0}$ and $R_{g0}$ from (56) and (54); and the number of susceptible is directly obtained from (2) taking $N_{gt} = N_{g0}$ for all *t* from the sources reported in Table 5.

In principle, knowing those numbers, and taking the daily deaths and fraction of vaccinated $\{f_{gt},\lambda _{gt}\}_{g=1,t=1}^{G,{\mathfrak {T}}}$ from the data, we could end up with a system of $4\times G$ equations, given by (59) to (62), and $4\times G$ unknowns, $\{\rho _{gt},S_{gt+1},C_{gt+1},V_{gt+1}\}_{g=1}^{G}$. However, the large number of zero deaths encountered in many periods make the system indeterminate many times when the geographical component is considered. The solution that we have adopted to solve this problem is assuming in the calibration of $\rho _{g}$ that $\kappa =0$. In this way, the system for each region simplifies and becomes independent of other areas. Hence, for each period $t\in [1,{\mathfrak {T}}]$ and region $g\in [1,G]$, we first recover $\rho _{gt}$ from (59) and then $\{S_{gt+1},C_{gt+1},V_{gt+1}\}_{g=1}^{G}$ from the other three equations.

## Footnotes

1 Compared to the sectoral structure presented by Caliendo and Parro (2015), the main difference with ours is that we consider that final consumption products can be cross-regional borders. The reason is that some of them, like tourism, can be important for the propagation of the virus and are tradable.

2 Wilson (2010) surveys the literature on the links between geography and infectious diseases and notes that socioeconomic conditions, public health infrastructure, urban versus rural environments, density and mobility of the population are important factors explaining the types and abundance. In the context of COVID-19 across the USA, Desmet and Wacziarg (2021) document spatial factors such as population density and distance to major airports that can explain the spatial variation in COVID-19 severity.

3 Several papers have highlighted the significance of infectious diseases in developing countries. In one such contribution, Chakraborty et al. (2010) introduce rational disease behavior in a general equilibrium framework, focusing on the effects of the burden of malaria and the HIV infection on economic development. They show that these diseases can create economic growth traps. In developed nations, similar evidence arises. For instance, focusing on European pandemics going back to the 14th century, Jordà et al. (2022) find important long-run economic consequences even after 40 years.

4 In the case of COVID-19, virus transmission through transportation primarily occurs from one human to another, as the main mode of transmission is through respiratory means. However, although limited, there has been some evidence suggesting the potential for COVID-19 transmission from products to humans, particularly from frozen foods. For example, Liu et al. (2020) found COVID-19 particles in imported frozen products close to entering the distribution network in Qingdao, China.

5 Another difference in our model is that deaths represent a labor supply shock. However, its impact is minor given the relatively low proportion of deaths attributed to COVID-19 compared to the total population. Importantly, our main focus is not on how production is directly affected by the pandemic, but rather on understanding the impact of trade links and disease containment policies on the spread of the COVID-19 pandemic. More significant labor supply shocks stemming from the pandemic include lockdowns (which we also consider as a consequence of mobility restrictions) and social distancing policies; see Brodeur et al. (2021), Section 5.1, for a review of the literature.

6 Resolving cases are infected individuals that cannot infect other people. Fernández-Villaverde and Jones (2022) suggest that distinguishing between infection and recovery periods matters for the model to fit the data well with biologically sensible parameters.

7 The assumption of a unitary elasticity of substitution in consumption might seem restrictive at first. However, it is worth pointing out that consumption in our framework denotes consumption of gross output, that is, final consumption expenditure. Herrendorf et al. (2013) estimate an elasticity of substitution in the range of 0.85–0.89, but also show that an elasticity of 1 can fit aggregate consumption shares as good as a CES. As the number of sector increases, our assumption of a unitary elasticity becomes more credible.

8 In order to derive (9), notice that the indirect utiity functions for working ($W_{g}^{L}$) and non-working ($W_{g}^{NL}$) individuals are, respectively,

$$\begin{aligned} W_{g}^{L}=\frac{1}{P_{g}}\left( w_{g}-t_{g}+\frac{{\mathcal {F}}_{g}+{\tilde{D}}_{g}}{N_{g}}-\tau _{g}\right) \ \text {and\ }\ W_{g}^{NL}=\frac{1}{P_{g}}\left( s_{m}+\frac{{\mathcal {F}}_{g}+{\tilde{D}}_{g}}{N_{g}}-\tau _{g}\right) . \end{aligned}$$

Defining $N_{g}W_{g}=L_{g}W_{g}^{L}+(N_{g}-L_{g})W_{g}^{NL}$ as total welfare in a location, $W_{g}$ is given by

$$\begin{aligned} W_{g}=\frac{L_{g}}{N_{g}}W_{g}^{L}+\left( 1-\frac{L_{g}}{N_{g}}\right) W_{g}^{NL}, \end{aligned}$$

which, substituting each of the indirect utility functions, and recalling that $s_{m}=w_{g}-t_{g}=$ $w_{g}L_{g}/N_{g}$ and that $l_{g}$ represents the fraction of working individuals in a location *g*, we get Eq. (9).

9 Costinot and Rodríguez-Clare (2014) named this approach exact hat-algebra.

10 We assume that the geographic component only operates while working. This can be seen as a restrictive assumption, as tourists, for example, can also spread the disease while walking in the streets. For simplicity, we abstract from these considerations. As a result, the contribution of the trade component may be underestimated.

11 It can be shown that the basic reproduction number of the disease, ${\mathcal {R}}_{0}$, increases in our setup with the level of trade integration between two regions, represented by ${\tilde{X}}_{ig}$; see Appendix A for the details. The basic reproduction number ${\mathcal {R}}_{0}$ represents the average number of secondary infections produced by a typical case of infection in a population where everyone is susceptible.

12 To better realize that $\kappa$ and ${\tilde{X}}_{ig}$ serve as weighting factors, notice that if, for example, $\rho _i = \rho$, $I_{i}/N_{i} = I/N$ and $\Lambda _{i} = 1$ for all *i*, then Eq. (46) implies that $\Phi _{g} = \rho I/N$.

13 Due to the large number of observations, these and other parameter and variable values are not reported in the paper. They are available from the authors upon request.

14 For the calibration of the disease parameters and initial values, ROW was assumed to be composed by China, the USA, and Switzerland. This means that for both the EU27 and the UK, we consider at least 70% of the trade volumes with other areas.

15 This problem could be partially solved through singular value decomposition and applying a least-squares method. However, the gap between predicted and actual deaths was always significantly worse when using this alternative procedure.

16 The impact of the productive lockdown during the pandemic varies substantially across industries and locations. As Fana et al. (2020) argues, countries primarily regulated these lockdowns by distinguishing between essential and not essential activities. Activities identified as essential, such as health, food, security, education, and administrative services, generally continued to operate. In contrast, non-essential activities faced varying degrees of closure during lockdown periods.

17 Recall that Fig. 10 depicts average values of the parameter $\rho _{g}$ across NUTS2 regions. Its continuous rise over the first month is a consequence of a sequence of additional regions being affected by the pandemic.

18 While its impact on policy seems limited, Sect. 6.3.2 demonstrates that the geographic component made a substantial contribution to the overall spread of the pandemic.

19 142 is the total number of days in our sample.

20 Under the policies implemented by the best performer, the three European regions that would have saved the most lives are all Polish. Lódzkie (PL11), Lubelskie (PL31), and Podlaskie (PL34) would have achieved life-saving rates exceeding 86% of the lives lost to COVID-19. These numbers, however, are not displayed in Table 15.
